# Mediterranean G6PD variant mitigates expression of DNA methyltransferases and right heart pressure in experimental model of pulmonary hypertension

**DOI:** 10.1016/j.jbc.2022.102691

**Published:** 2022-11-11

**Authors:** Christina Jacob, Atsushi Kitagawa, Christina Signoretti, Monika Dzieciatkowska, Angelo D’Alessandro, Aaditya Gupte, Shakib Hossain, Catherine A. D’Addario, Rakhee Gupte, Sachin A. Gupte

**Affiliations:** 1 Department of Pharmacology, New York Medical College, Valhalla, New York, USA; 2 Department of Biochemistry & Molecular Genetics, School of Medicine, University of Colorado Denver - Anschutz Medical Campus, Aurora, Colorado, USA

**Keywords:** experimental, rats, pulmonary arterial hypertension, epigenetics, histone, methylation, acetylation, DNA, methyltransferases, hypoxia, normoxia, Sugen5416, VEGF blocker, AB, ammonium bicarbonate, G6PD, glucose-6-phosphate dehydrogenase, MeDIP, methylated DNA immunoprecipitation, MI, methylation index, MS, mass spectrometry, MS-PCR, methylation-specific PCR, PH, pulmonary hypertension, qPCR, quantitative PCR, RT-PCR, real-time PCR, RVDP, RV end diastolic pressure, RVSP, RV systolic pressure, SMC, smooth muscle cell

## Abstract

DNA methylation potentially contributes to the pathogenesis of pulmonary hypertension (PH). However, the role of DNA methyltransferases (DNMTs: 1, 3a, and 3b), the epigenetic writers, in modulating DNA methylation observed in PH remains elusive. Our objective was to determine DNMT activity and expression in the lungs of experimental rat models of PH. Because the activity of DNMTs is metabolically driven, another objective was to determine the role of glucose-6-phosphate dehydrogenase (G6PD) in regulating DNMT expression and activity in the lungs of novel loss-of-function Mediterranean G6PD variant (G6PD^S188F^) rats. As outlined for modeling PH, rats injected with sugen5416 (SU) were placed in a hypoxia (Hx) chamber set at 10% oxygen for 3 weeks and then returned to normoxia (Nx) for 5 weeks (SU/Hx/Nx). Rats kept in atmospheric oxygen and treated with SU were used as controls. We assessed the activity and expression of DNMTs in the lungs of rats exposed to SU/Hx/Nx. WT rats exposed to SU/Hx/Nx developed hypertension and exhibited increased DNMT activity and *Dnmt1* and *Dnmt3b* expression. In G6PD^S188F^ rats, which developed less of a SU/Hx/Nx-induced increase in right ventricle pressure and hypertrophy than WT rats, we observed a diminished increase in expression and activity of DNMTs, DNA hypomethylation, increased histone acetylation and methylation, and increased expression of genes encoding NOS3 and SOD2—vascular-protective proteins. Collectively, increased DNMTs contribute to reduced expression of protective genes and to the pathogenesis of SU/Hx/Nx-induced experimental PH. Notably, G6PD regulates the expression of DNMTs and protective proteins in the lungs of hypertensive rats.

DNA methylation is regulated by DNA methyltransferase (DNMT; 1, 3a, and 3b) and DNA demethylase or ten-eleven translocation (TET; 1, 2, and 3) methylcytosine dioxygenases (1). Recently, we and others reported that differential DNA methylation and a reduction in TET2 expression may be key to the pathogenesis of pulmonary hypertension (PH) in mice and humans (2, 3). In those studies, our laboratory found that DNA methylation–dependent transcription of PH-related genes and *Tet2* expression is suppressed in the lungs of hypoxic mice (2). Moreover, we observed that inhibiting glucose-6-phosphate dehydrogenase (G6PD) activity increases pulmonary *Tet2* expression in hypoxic mice and reduces hypoxia-induced PH (2). However, two questions arise from those studies: (1) does the DNMT expression and activity increase or decrease in PH and (2) is G6PD involved in regulating the expression and activity of DNA methylation writers in experimental models of PH?

While loss-of-function mutations in *TET2* are associated with diverse blood cell malignancies in humans (4), loss of a single *TET* enzyme is not sufficient to efficiently promote malignancy (5). We, therefore, hypothesized that alteration of DNMT-catalyzed DNA methylation to modify gene expression must occur in PH. To test that idea, we assessed *Dnmt* (1, 3a, and 3b) expression in lungs of rat exposed to sugen5614/hypoxia/normoxia (SU/Hx/Nx), a model of experimental PH (6). In addition, to determine whether *Dnmt* expression is driven by G6PD, we performed experiments in a novel rat model harboring the loss-of-function Mediterranean *G6pd* variant (G6PD^S188F^), which we recently generated (7), and in their WT littermates. G6PD deficiency is a common enzymopathy in humans, and there are conflicting reports regarding the effects of G6PD deficiency on the progression and severity of PH. Some studies suggest the Mediterranean G6PD^S188F^ variant makes no contribution to sickle cell anemia–associated PH, while others suggest the frequency of G6PD mutation is higher among idiopathic PH patients than the general population (8, 9, 10). We, therefore, used Mediterranean G6PD^S188F^ variant rats to assess the role of G6PD in the progression and severity of SU/Hx/Nx-induced experimental PH.

Our findings suggest that increased DNMT-catalyzed DNA methylation potentially contributes to the development and progression of PH pathology. What is more, they further suggest that G6PD contributes to the increase in DNMT expression and activity, as well as modify expression of linker histone1H1 and increase histone acetylation/methylation, resulting in the higher expression of genes encoding endothelial nitric oxide synthase 3 (NOS3), mitochondrial superoxide dismutase 2 (SOD2), and other vascular biology-related proteins in the lungs of rats with SU/Hx/Nx-induced experimental PH.

## Results

### SU/Hx/Nx augments folate pathway protein expression in lungs of WT but not G6PD^S188F^ rats

**Figure 1:**
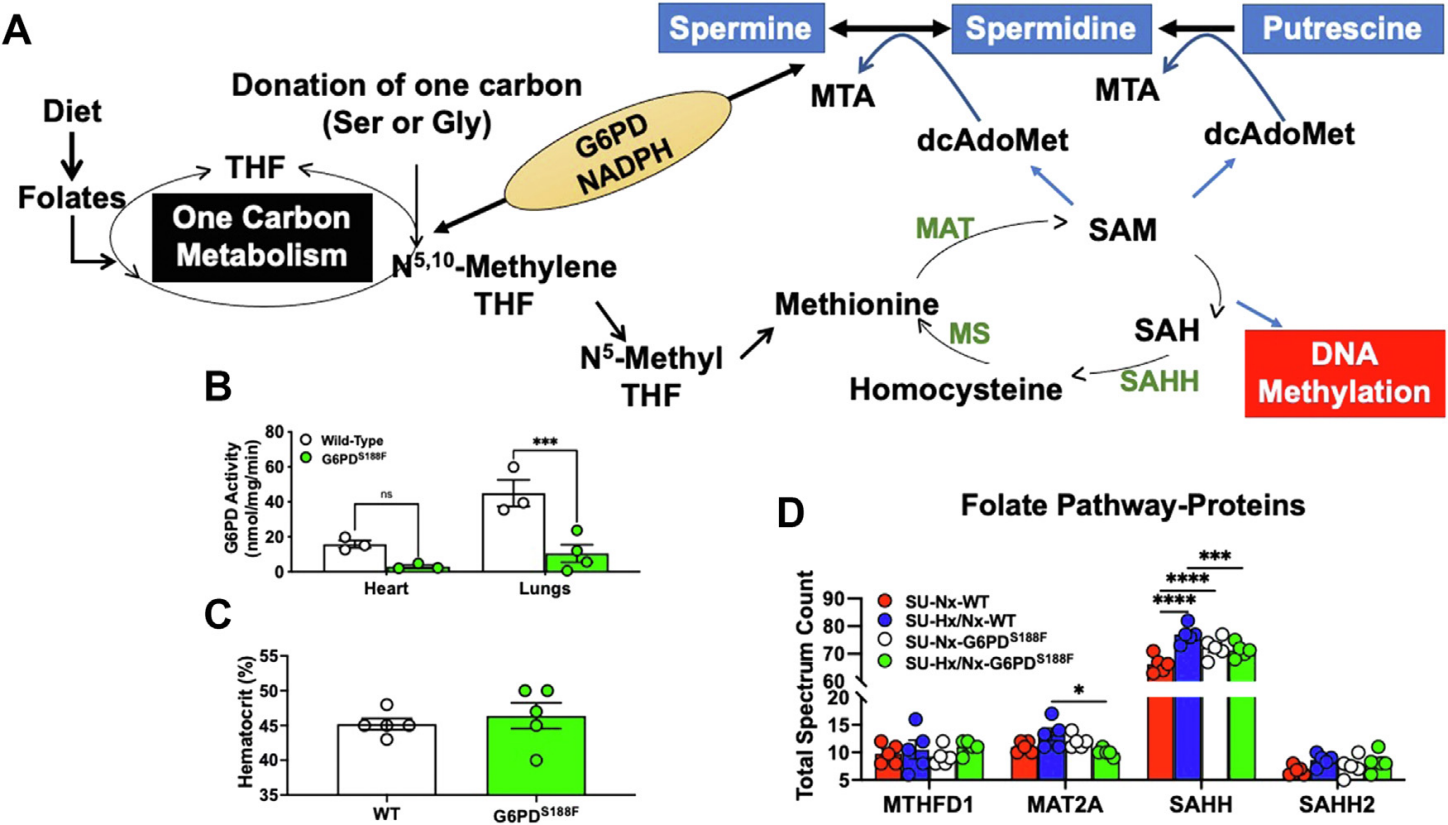
**Elevated pulmonary folate pathway proteins in SU/Hx/Nx WT but not G6PD**^**S188F**^**rats**. *A*, a schematic illustration connecting G6PD-derived NADPH-dependent one-carbon metabolism and polyamine pathway to DNA methylation. *B*, G6PD activity in the heart and lungs of Mediterranean G6PD variant (G6PD^S188F^) rats is considerably lower (<70–80%) than in WT rats. *C*, hematocrit is not different between G6PD^S188F^ and WT rats. Rats receiving sugen5416 (SU) were kept in normoxia (21% O_2_) or exposed to hypoxia (10% O_2_) for 3 weeks followed by normoxia (21% O_2_) for 5 weeks. Expression levels of one carbon metabolism/folate pathway proteins methionine adenosyl transferase 2A (MAT2A), S-Adenosyl-L-Homocysteine Hydrolase (SAHH), and S-Adenosyl-L-Homocysteine Hydrolase-2 (SAHH2) in lungs were determined by Western blotting and by quantitative proteomics. *D*, quantitative proteomics revealed MAT2A, SAHH, and SAHH2 are increased or showed increasing trend in SU/Hx/Nx than SU/Nx in WT but not in G6PD^S188F^ rats. N (number of rats) = 5 in each group. Comparisons among all four groups were made using two-way ANOVA and between two groups using Student’s *t* test. ∗∗*p* < 0.005 and ∗∗∗∗*p* < 0.0001. G6PD, glucose-6-phosphate dehydrogenase.

**Figure 2:**
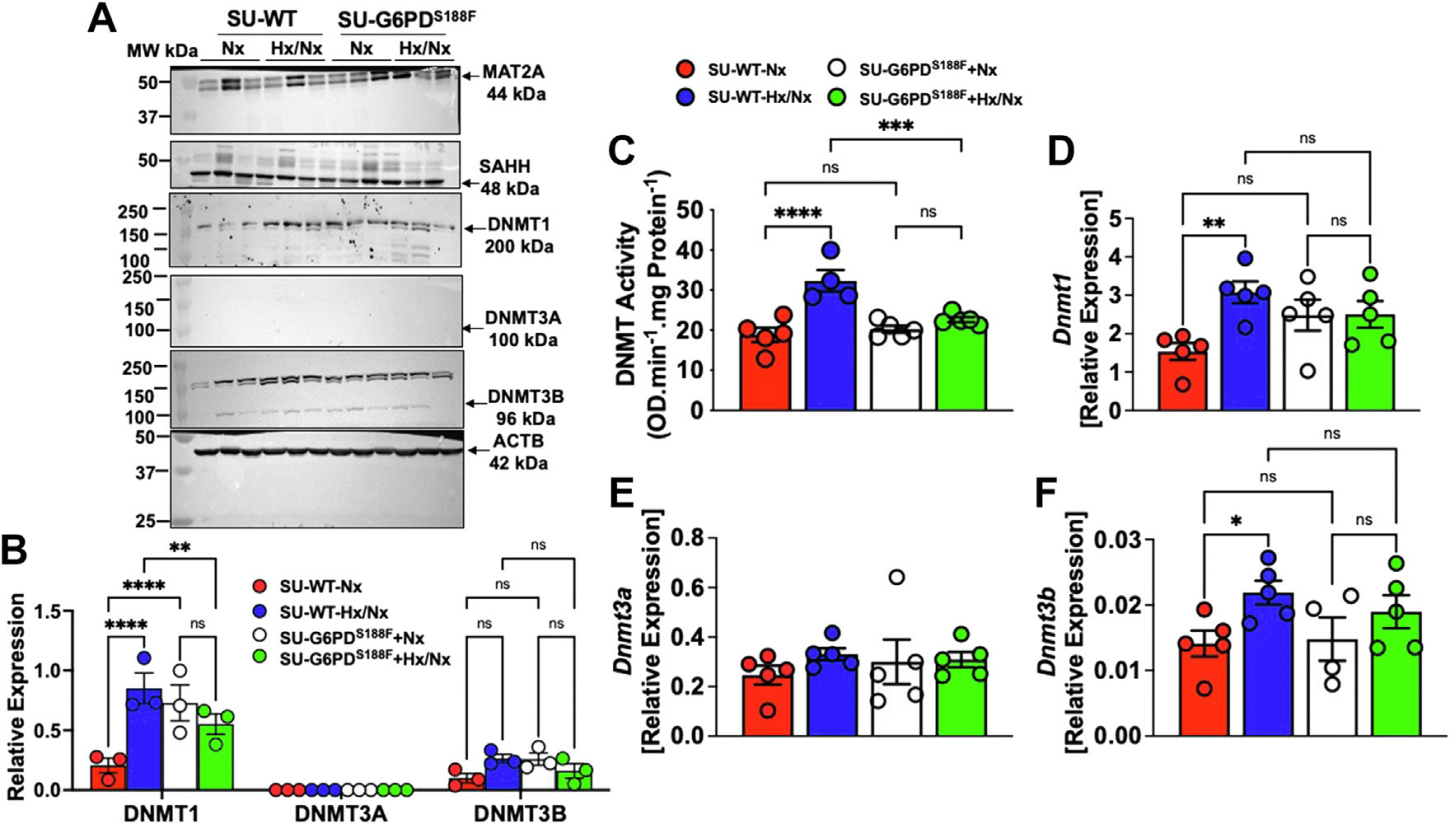
**Elevated pulmonary DNMT expression and activity in SU/Hx/Nx WT but not G6PD**^**S188F**^**rats**. Rats receiving sugen5416 (SU) were kept in normoxia (21% O_2_) or exposed to hypoxia (10% O_2_) for 3 weeks followed by normoxia (21% O_2_) for 5 weeks. *A*, Western blot of three separate experiments show no differences in expression of MAT2A or SAHH but higher expression of DNMT1 and DNMT3B between WT and G6PD^S188F^ rats exposed to SU/Hx/Nx and SU/Nx. *B*, summary results showing higher expression of DNMT1 and DNMT3B in lungs of rats exposed to SU/Hx/Nx and SU/Nx. DNMT3A was not detected. *C*, pulmonary DNMT activity was higher in lungs of WT, but not G6PD^S188F^, rats that received SU (20 mg/kg; I.P.) kept in hypoxia + normoxia (Hx; *blue bar*) than in normoxia (Nx; *red bar*). N (number of rats) = 5 in each group. *D*–*F*, RT-PCR analysis showing that pulmonary expression of *Dnmt1*, *Dnmt3a*, and *Dnmt3b* in WT and G6PD^S188F^ rats with SU exposed to hypoxia changes as compared to their respective normoxia control. Pulmonary expression of *Dnmt1* and *Dnmt3b*, but not *Dnmt3a*, was higher in WT but not in G6PD^S188F^ rats with SU kept in hypoxia + normoxia (Hx; *blue bar*) than in normoxia (Nx; *red bar*). Comparisons among all four groups were made using two-way ANOVA. ∗*p* < 0.05; ∗∗*p* < 0.005; ∗∗∗*p* < 0.0005; and ∗∗∗∗*p* < 0.0001. G6PD, glucose-6-phosphate dehydrogenase; RT-PCR, real-time PCR.

As illustrated (Fig. 1*A*), G6PD-NADPH–dependent one-carbon metabolism and polyamine pathway metabolites (s-adenosylmethionine and spermine, respectively) are methyl group donors and regulators of DNA methylation (11, 12) and are elevated in lungs/fibroblasts/smooth muscle cells (SMCs) of PH patients (2, 13); we sought to determine the role of G6PD in modulating expression of enzymes of folate pathway and activity of DNMTs in lungs of SU/Hx/Nx rats. To accomplish that goal, we used a recently developed loss-of-function Mediterranean G6PD variant (G6PD^S188F^) rat (7). As expected, G6PD activity was 70% to 80% lower in the heart and lungs of G6PD^S188F^ than WT rats (Fig. 1*B*). Next, because G6PD deficiency causes hemolytic anemia, we compared hematocrit levels between G6PD^S188F^ and WT rats and found no difference in the hematocrit between both genotypes (Fig. 1*C*). Further, we did not observe hemolysis in G6PD^S188F^ rats and the hematocrit between SU/Hx/Nx G6PD^S188F^ (49 ± 1%) and WT (49 ± 1%) rats was not different. Interestingly, quantitative mass spectrometry (MS) proteomics results revealed enzymes that convert s-adenosyl-l-homocysteine to homocysteine (SAHH) increased and methionine to s-adenosylmethionine (MAT2A) and s-adenosyl-l-homocysteine to homocysteine (SAHH2) tend to increase lungs of SU/Hx/Nx WT rats as compared to SU/Nx WT rats (Fig. 1*D*). In contrast, MAT2A and SAHH decreased in SU/Hx/Nx G6PD^S188F^ rats as compared to WT rats. While we did not find differences in the expression levels of enzymes in the one-carbon and folate pathway by Western blotting (Fig. 2*A*).

### SU/Hx/Nx increases total DNMT activity and *Dnmt1* and *Dnmt3b* expression in lungs of WT rats

Next, we measured DNMT expression and activity in the lungs of rats exposed to SU/Hx/Nx, SU/Nx, hypoxia (Hx; 10% O_2_), and normoxia (Nx: 21% O_2_). SU/Hx/Nx increased DNMT1 and DNMT3B expression (Fig. 2, *A* and *B*). DNMT3A was not detectable. Although pulmonary DNMT activity was similar in hypoxic and normoxic rats, it was significantly higher in SU/Hx/Nx than SU/Nx rats (Fig. 2*C*). While Hx increased pulmonary expression of *Dnmt3a* (Nx: 0.20 ± 0.04 and Hx: 0.54 ± 0.20; *p* < 0.05) and *Dnmt3b* (Nx: 0.009 ± 0.002 and Hx: 0.026 ± 0.007; *p* < 0.05) but not *Dnmt1* (Nx: 1.68 ± 0.63 and Hx: 2.79 ± 1.20; NS), SU/Hx/Nx increased expression of *Dnmt1* and *Dnmt3b* (Fig. 2, *D* and *F*), as compared to respective SU/Nx control, in rats. SU/Hx/Nx did not significantly affect *Dnmt3a* expression in rats (Fig. 2*E*).

### SU/Hx/Nx does not increase DNMT activity and *Dnmt1* and *Dnmt3b* expression in lungs of G6PD^S188F^ rats

Since we found G6PD^S188F^ variant prevented SU/Hx/Nx-elicited expression of protein involved in one carbon metabolism or folate pathway (Fig. 1*D*) and correspondingly pulmonary DNMT activity did not increase in SU/Hx/Nx (22.7 ± 0.63 OD/min/mg Protein) as compared with normoxic (20.2 ± 0.88 OD/min/mg Protein) G6PD^S188F^ rats (Fig. 2*C*), we measured expression of genes encoding DNMTs in the lungs of SU/Hx/Nx and SU/Nx G6PD^S188F^ rats. Interestingly, there was no difference in pulmonary expression of *Dnmt1* (Fig. 2*D*), *Dnmt3a* (Fig. 2*E*), and *Dnmt3b* (Fig. 2*F*) in SU/Hx/Nx as compared to SU/Nx G6PD^S188F^ rats. Consistently, expression of DNMT1 and DNMT3B did not increase in SU/Hx/Nx G6PD^S188F^ rats (Fig. 2, *A* and *B*).

### SU/Hx/Nx increases expression DNMT1 and DNMT3B in occluded arteries and alveoli of WT rats

**Figure 3:**
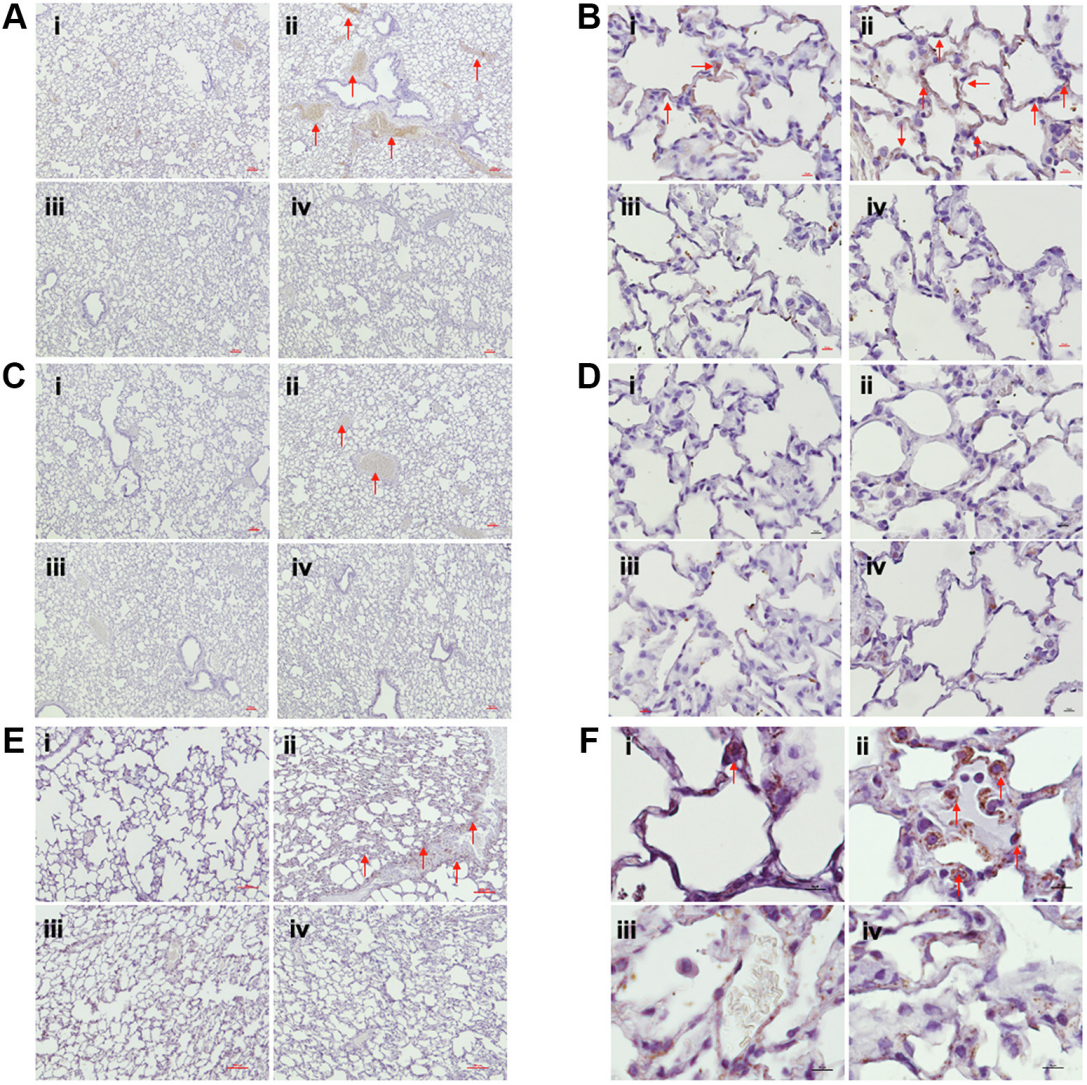
**DNMTs increased in arteries and alveoli of SU/Hx/Nx WT but not G6PD**^**S188F**^**rats**. *A*–*F*, a representative micrograph (4× and 40× magnification; 4×: scale bar is 100 μm and 40×: scale bar is 10 μm) of immunohistochemistry results from four different experiment show staining of different DNMTs in lungs of SU/Nx-WT and SU/Hx/Nx-WT (i and ii) and SU/Hx/Nx--G6PD^S188F^ (iii and iv) rats. (A-i and ii) DNMT1 and (E-i and ii) DNMT3B expression (*brown staining*) increased in SU/Hx/Nx-WT as compared with SU/Nx-WT rat arteries and alveoli indicated by *red arrows*. (A-iii and iv) DNMT1 and (E-iii and iv) DNMT3B show less intense staining in SU/Hx/Nx-G6PD^S188F^ and SU/Nx-G6PD^S188F^ rat arteries and alveoli. (C- and D-iii and iv). G6PD, glucose-6-phosphate dehydrogenase.

Next, we performed immunohistochemistry to determine expression of DNMTs in lungs of WT and G6PD^S188F^ rats. Immunohistochemical assay revealed DNMT1 expression (brown staining) increased in and around the arteries (Fig. 3, *A*–*I* and -ii) and in more alveoli (Fig. 3, *B*–*I* and -ii) of SU/Hx/Nx rats as compared to SU/Nx-WT rats. While we do not see intense brown staining for DNMT3A (Fig. 3, *C* and *D*), which corroborated the Western blot results (Fig. 2, *A* and *B*), we found increased brown staining for DNMT3B around the arteries (Fig. 3, *E*–*I* and -ii) and in alveoli (Fig. 3, *F*–*I* and -ii) of SU/Hx/Nx-WT rats as compared to SU/Nx-WT rats. To this regard, we found less intense staining areas for all DNMTs around arteries (Fig. 3, *A*–*C*-, and *E*-iii and -iv) and alveoli (Fig. 3, *B*–*D*-, and *F*-iii and -iv) of SU/Hx/Nx-G6PD^S188F^ rats as compared to SU/Nx-G6PD^S188F^ rats.

### SU/Hx/Nx increases methylation of the *Sod2*, *Abra*, *and Serpine1* promoter in the lungs of WT but not G6PD^S188F^ rats

**Figure 4:**
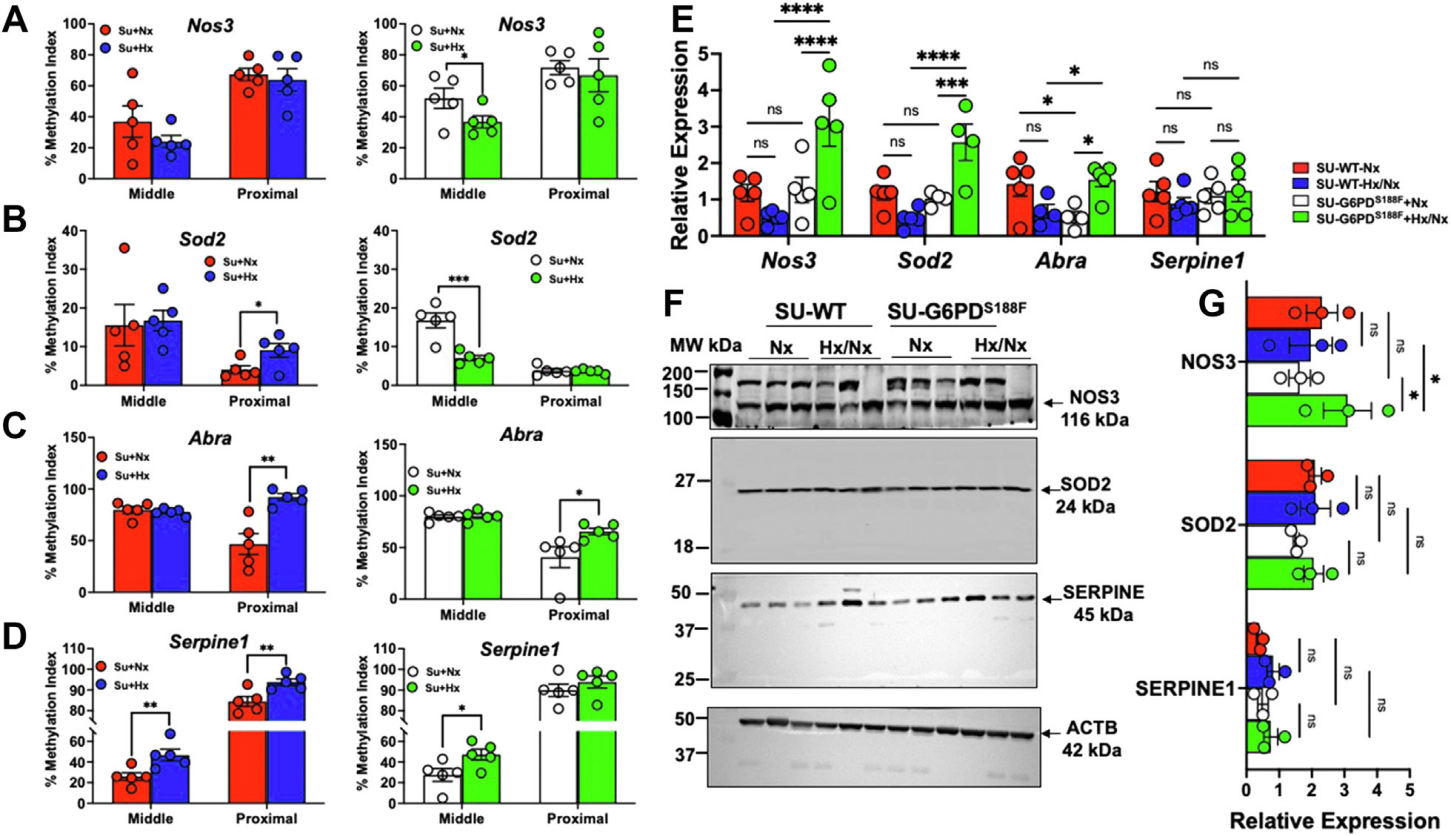
**Methylation and expression of the *Nos3*, *Sod2*, *Abra*, and *Serpine1* in lungs of SU/Hx/Nx WT and G6PD**^**S188F**^**rats**. Cumulative results showing that the methylation indexes for CpG islands in the promoter (middle promoter and proximal promoter) of the (*A*) *Nos3*, (*B*) *Sod2*, (*C*) *Abra*, and (*D*) *Serpine1* in the lungs from SU/Hx/Nx than normoxic WT (left panels; SU/Nx: *red bars* and SU/Hx/Nx: *blue bars*) and G6PD^S188F^ (right panels; SU/Nx: *white bars* and SU/Hx/Nx: *green bars*) rats. The methylation index increased in SU/Hx/Nx WT but not G6PD^S188F^ rats. Primers were designed to CpG regions in the middle and proximal to the transcription start site in each gene. *E*, RT-PCR results showing that pulmonary expression of *Nos3*, *Sod2*, *Abra*, and *Serpine1* is lower in SU/Hx/Nx than normoxic WT but not G6PD^S188F^ rats. A representative Western blot of three separate experiments (*F*) and summary results (*G*) showing expression of NOS3, SOD2, and SERPINE1 in the lungs of rats that received sugen5416 (SU) kept in hypoxia + normoxia than in normoxia. N (number of rats) = 5 in each group (panels *A*–*D* and *E*). Comparisons among all four groups were made using two-way ANOVA and between two groups using Student’s *t* test. ∗*p* < 0.05; ∗∗*p* < 0.005; and #*p* < 0.05. G6PD, glucose-6-phosphate dehydrogenase; RT-PCR, real-time PCR.

Because DNMT1 and DNMT3B expression and activity was higher in the lungs of SU/Hx/Nx than SU/Nx WT rats, we performed methylated DNA immunoprecipitation (MeDIP)-PCR to determine differential methylation status of the methylation-sensitive noncoding H19 gene imprinting center region 1 (H19ICR1) sequence in G6PD^S188F^ *versus* WT rat genome. We found enriched methylated H19ICR gene region in lungs of SU/Hx/Nx (1.57 ± 0.27) as compared with SU/Nx (1.08 ± 0.15) WT rats but not G6PD^S188F^ rats (SU/Hx/Nx: 1.13 ± 0.11 and Su/Nx: 1.20 ± 0.01). Next, we performed methylation-specific PCR (MS-PCR) using primers designed (Table 1) to amplify methylated and unmethylated CpG islands in the middle and proximal regions of the *Nos3*, *Sod2*, *Abra*, and *Serpine1* promoters in the lungs of SU/Hx/Nx *versus* SU/Nx rats (Fig. 4). We selected *Nos3* and *Sod2* promoters because products of these genes are implicated in the pathogenesis of pulmonary hypertension (14, 15) and *Abra* and *Serpine1* promoters because proteins encoded by these genes are involved in vascular smooth muscle biology and thrombosis (16, 17) and we recently observed that expression of *Serpine1* and *Abra* was altered in aortas from rats fed a high-fat diet (18). We found that the methylation index (MI) was higher in the middle and proximal regions of the *Sod2*, *Abra*, and *Serpine1* promoters in SU/Hx/Nx than SU/Nx WT rats (Fig. 4, *B*–D). Very interestingly, the MI decreased in the middle and proximal region of *Nos3*, *Sod2*, *Abra*, and *Serpine1* promoters in SU/Hx/Nx G6PD^S188F^ rats (Fig. 4, *A*–*D*).

**Table 1:** MS-PCR primers for middle and proximal regions of the gene promoter

Primer Name	Forward	Reverse
*Serpine1*-MM	5′-TATCGCGTACGTAATTAAAAC-3′	5′-AAAATATATACCGCCGTACC-3′
*Serpine1*-MU	5′-ATGTAAGAAGAAAGTGGGGT-3′	5′-TCCAACTCACAAAAATCAAC-3′
*Serpine1*-PM	5′-CGTTAAGGGTTTAGACGAT-3′	5′-CCCAATAACTTACTAAAACACG-3′
*Serpine1*-PU	5′-GTTAAGGGTTTAGATGATTGAT-3′	5′-TCCCAATAACTTACTAAAACACA-3′
*Abra*-MM	5′-TTAGAGCGTTGGTTTAGTAAGC-3′	5′-TTTCCGAAACTAAAAACCGA-3′
*Abra* -MU	5′-TGGTTAGAGTGTTGGTTTAGTAAGT-3′	5′-CAAAACTAAAAACCAAACCCA -3′
*Abra* -PM	5′-GTTTGTGACGGTGGAAATTAT-3′	5′-AAACAAAACACCGTCAAAAC-3′
*Abra* -PU	5′-GTGTTTGTGATGGTGGAAATTA -3′	5′-AAAACACCATCAAAACCATAAC-3′
*Nos3*-MM	5′-AGATTGTTTCGGTTTGTTGTAT-3′	5′-TACAACCTCGACCGATCCT-3′
*Nos3*-MU	5′-GAAGATTGTTTTGGTTTGTTGTA-3′	5′-AACCTCAACCAATCCTCCTC-3′
*Nos3*-PM	5′-GCGTATTAGTTGGCGTTTTA-3′	5′-CCGTATCAATTCAACCATCA-3′
*Nos3*-PU	5′-TTGTGTAGTGTATTAGTTGGTGTTT-3′	5′-CCCCATATCAATTCAACCAT-3′
*Sod2*-MM	5′-TAAGGCGGTTCGAGAAGAG-3′	5′-GCGAACTTACCACGACCACG-3′
*Sod2*-MU	5′-GGTTAAGGTGGTTTGAGAAGA-3′	5′-CACAAACTTACCACAACCACAC-3′
*Sod2*-PM	5′-AAGTTGGGTCGTTCGTGTC-3′	5′-AACCGCGCGTCTACTCTAC-3′
*Sod2*-PU	5′-GTTGTTTGTGTTGTGGTTTTT-3′	5′-ACCACACATCTACTCTACAACATC-3′

MM= methylated target in middle region of the promoter; MU= unmethylated target in middle region of the promoter; PM = methylated target in proximal region of the promoter; PU= unmethylated target in proximal region of the promoter.

### SU/Hx/Nx decreases expression of genes encoding NOS3, SOD2, and ABRA in the lungs of WT but not G6PD^S188F^ rats

Further, we wanted to determine whether expression of these genes was affected by the elevated DNMT activity seen in the lungs of SU/Hx/Nx rats. Using real-time PCR (RT-PCR), we found that levels of pulmonary *Nos3*, *Sod2*, *Abra*, and *Serpine1* expression were decreased in SU/Hx/Nx compared to SU/Nx WT rats (Fig. 4*E*). In contrast, expression of *Nos3* and *Sod2* was augmented in SU/Hx/Nx compared to SU/Nx G6PD^S188F^ rats (Fig. 4*E*). Next, we performed Western blotting for the expression of NOS3, SOD2, and SERPINE1. As shown in representative blots and summary results, the expression of NOS3 and SOD2 showed an increasing trend in SU/Hx/Nx as compared to SU/Nx G6PD^S188F^ rats (Fig. 4, *F* and *G*), while expression of SERPINE1 showed increasing trend in SU/Hx/Nx as compared with SU/Nx WT and G6PD^S188F^ rats (Fig. 4, *F* and *G*). Interestingly, expression of NOS3 increased in lungs of SU/Hx/Nx-G6PD^S188F^ rats as compared to SU/Hx/Nx-WT rats (Fig. 4*G*). Due to the lack of a specific antibody against rat ABRA, we performed Westerns with antibodies against human and mouse species, but ABRA signals were undetectable (data not shown).

### Proteomic analysis reveals modification of the pulmonary expression of proteins in SU/Hx/Nx rats

Because DNMT expression and activity increased in SU/Hx/Nx-WT rats, we speculated this will alter or modify expression of several other genes and proteins, in addition to NOS3 and SOD2, in the lungs. Using quantitative MS, we detected pulmonary expression of 2300 proteins in rat lungs (Table S1). Pulmonary expression of proteins involved in glycolysis (ALDOA and IDH3B), Ca^2+^ handling (ITPR2), Gq receptor signaling (ARRB1), solute transporter (SLC44A2), and the complement cascade protein (C9) was higher in SU/Hx/Nx than SU/Nx WT rats but not G6PD^S188F^ rats (Table 2). By contrast, pulmonary expression of proteins involved in the mitochondrial respiratory chain (ACCA2 and ATP5F1A), PKA signaling (PRKAR1A), Ca^2+^ handling (SERCA2/ATP2A2 and CACNA2D1), inhibition of neutrophil elastase and cathepsin G (SERPINA1 and SERINA3K), differentiation of SMC phenotype (CSRP1), and gene transcription (NPM1 and SART1) were all lower in SU/Hx/Nx than SU/Nx WT rats but not G6PD^S188F^ rats (Table 2). Proteomic data were deposited into MassIVE (ftp://MSV000089037@massive.ucsd.edu or ftp://massive.ucsd.edu/MSV000089037/).

**Table 2:** Total spectrum count of identified proteins in the rat lungs

Accession Number	*Alternate ID*	G6PD^S188F^ + Su	G6PD^S188F^ + Su + Hx	WT + Su	WT + Su+ Hx
Mitochondrial Proteins					
THIM_RAT	Acaa2	24.2 ± 3.9	16.0 ± 3.2	23.3 ± 1.8	14.5 ± 1.6^a^
ATPA_RAT	Atp5f1a	72.0 ± 0.4	73.3 ± 5.1	75.5 ± 3.1	65.8 ± 1.6^a^
M2OM_RAT	Slc25a11	5.8 ± 0.4	8.0 ± 0.5^b^	6.0 ± 0.6	9.8±0.5^a^
Glycolysis Proteins					
ALDOA_RAT	Aldoa	63.0 ± 2.2	67.8 ± 2.3	65.0 ± 1.7	72.0 ± 3.2^a^
IDH3B_RAT	Idh3B	23.5 ± 1.3	23.5 ± 2.1	21.5 ± 2.6	28.8 ± 1.9^a^
PPP Proteins					
G6PD_RAT	G6pdx	17.3 ± 1.0^a^	21.3 ± 1.7^c^	29.5 ± 1.0	33.8 ± 2.6
TKT_RAT	Tkt	96.3 ± 1.5^a^	102.3 ± 7.3	107.3 ± 4.0	108.5 ± 2.8
Cell Signaling Proteins					
KAP0_RAT	Prkar1a	23.8 ± 2.4	22.3 ± 1.4	25.5 ± 2.5	17.3 ± 1.1^a^
ARRB1_RAT	Arrb1	17.8 ± 1.1	20.5 ± 0.6	18.8 ± 1.7	25.8 ± 2.8^a^
PDE5A_RAT	Pde5a	25.0 ± 2.0^a^	25.0 ± 1.2	30.5 ± 1.2	26.0 ± 3.6
Calcium Handling Proteins					
AT2A2_RAT	Atp2a2	42.0 ± 6.1	45.3 ± 7.0	47.3 ± 6.1	34.3 ± 2.0^a^
CA2D1_RAT	Cacna2d1	10.0 ± 1.7	7.3 ± 1.5	15.3 ± 2.1	10.5 ± 0.9^a^
ITPR2_RAT	Itpr2	4.3 ± 2.5	6.8 ± 2.7	3.5 ± 2.1	9.3 ± 1.8^a^
Serine Proteases					
A1AT_RAT	Serpina1	81.5 ± 8.9	76.8 ± 5.2^c^	94.0 ± 8.2	62.8 ± 4.0^a^
SPA3K_RAT	Serpina3k	107.0 ± 9.3	120.0 ± 7.9^c^	121.0 ± 10.5	74.5 ± 8.6^a^
Complement Proteins					
CO9_RAT	C9	35.3 ± 1.9	37.8 ± 4.1	33.3 ± 0.5	45.8 ± 3.1^a^
SMC Proteins					
CSRP1_RAT	Csrp1	50.0 ± 2.2	54.3 ± 2.1	64.8 ± 3.3	46.0 ± 3.7^a^
Nuclear and Transcription Proteins					
NPM_RAT	Npm1	21.0 ± 2.3	19.5 ± 1.3	24.0 ± 0.7	17.8 ± 1.3^a^
SNUT1_RAT	Sart1	5.0 ± 2.0	2.5 ± 1.5	8.5 ± 2.4	2.3 ± 2.3^a^
Other Proteins					
DHB11_RAT	Hsd17b11	6.5 ± 0.6	6.5 ± 0.6^c^	7.5 ± 1.5	11.5 ± 1.5^a^
RTN3_RAT	Rtn3	15.0 ± 0.9	17.3 ± 1.5	12.8 ± 1.1	20.5 ± 1.3^a^
SFTPA_RAT	Sftpa1	21.8 ± 2.6	25.3 ± 1.7	16.5 ± 0.6	24.5 ± 1.5^a^
SFXN3_RAT	Sfxn3	12.0 ± 2.2	10.3 ± 0.9^c^	10.5 ± 1.4	6.5 ± 1.0^a^
CTL2_RAT	Slc44a2	30.0 ± 1.4	32.3 ± 1.1^c^	28.0 ± 0.9	40.0 ± 2.3^a^
VWA5A_RAT	Vwa5a	17.3 ± 2.1	16.0 ± 2.3	22.3 ± 2.0	15.3 ± 2.1^a^
ST1A1_RAT	Sult1a1	15.8 ± 1.5	16.3 ± 2.3^c^	16.8 ± 1.3	22.8 ± 1.0^a^

Mean ± SEM; N = 4 in each group.

a *p* < 0.05 *versus* WT+Su.

b *p* < 0.05 *versus* G6PD^S188F^+Su.

c *p* < 0.05 *versus* WT+Su+Hx.

### SU/Hx/Nx alters linker histone1H1 expression in G6PD^S188F^ rats

**Figure 5:**
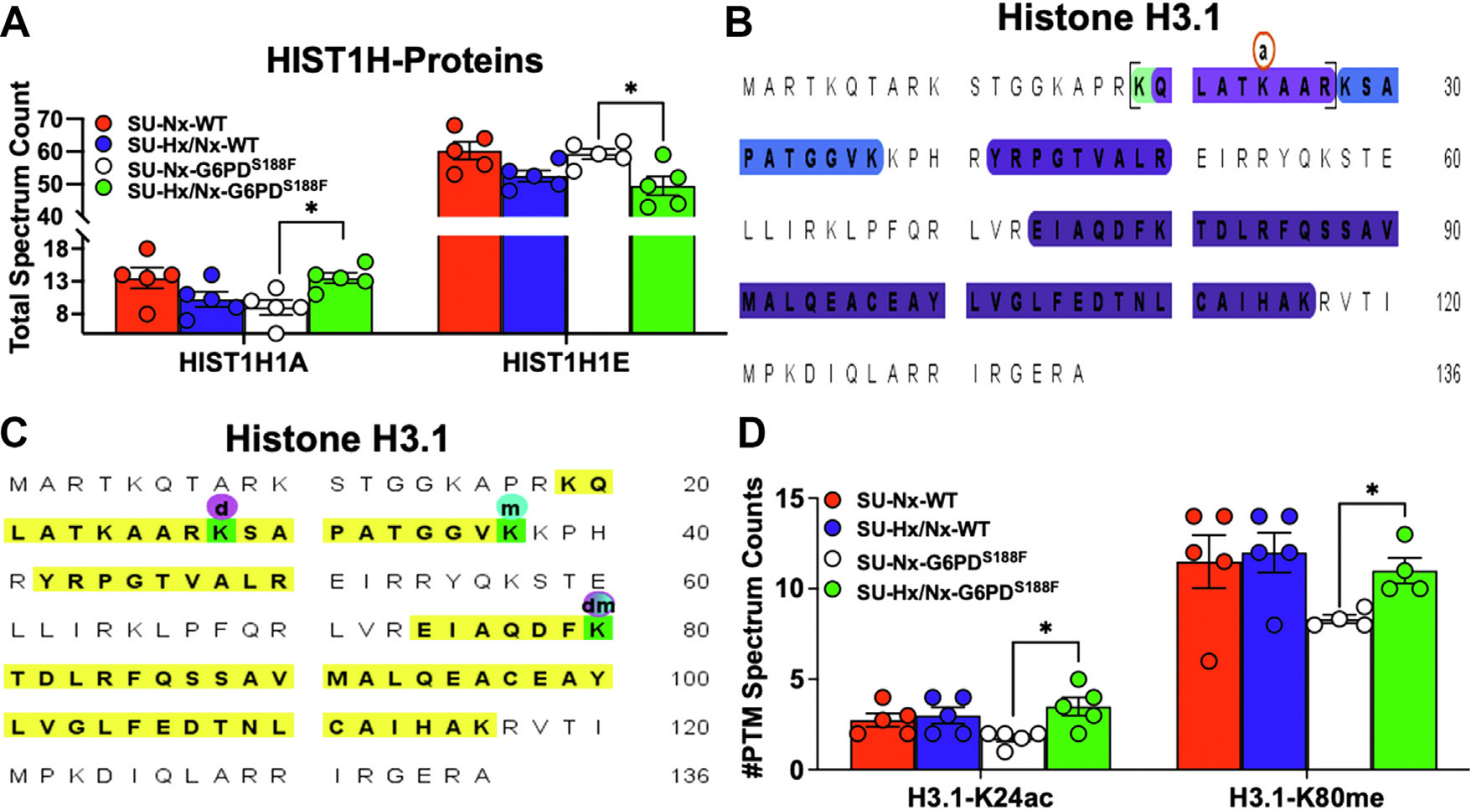
**Linker histone1H1 and acetylation and methylation of histone3 was increased SU/Hx/Nx G6PD**^**S188F**^**rats**. Mass spectrometry–based proteomic analysis shows (*A*) linker histone1H1 and (*B* and *C*) acetylation and methylation of Lys residues in H3 variants, H3.1, in rat lungs. Cumulative analysis of the (*A*) total spectrum count showing higher histone1H1A and lower histone1H1E and (*D*) posttranslation modification (PTM) spectrum counts showing higher H3.1-K24ac and H3.1-K80me levels, in the lungs of SU/Hx/Nx versus normoxic G6PD^S188F^ but not WT rats. N (number of rats) = 5 in each group. Comparisons between two groups were made using Student’s *t* test. ∗*p* < 0.05. G6PD, glucose-6-phosphate dehydrogenase.

Next, in quantitative MS-proteomic analysis (Table S1), we found expression of linker histone1H1A (H1.1) and histone1H1E (H1.4), which binds DNMT1 and DNMT3B and reduces methylation of genes (19), respectively, increased and decreased, in SU/Hx/Nx than SU/Nx G6PD^S188F^ rats (Fig. 5*A*). Histone1H1A and histone1H1E showed decreasing trends in SU/Hx/Nx than SU/Nx WT rats (Fig. 5*A*).

### Histone acetylation and methylation increases in lungs of SU/Hx/Nx and normoxic G6PD^S188F^ rats but not WT rats

Because it was recently proposed that posttranslation histone modification (acetylation and methylation) contributes to gene regulation and to the pathogenesis of pulmonary arterial hypertension (20), we speculated that histone modifications occur in the lungs of SU/Hx/Nx rats. Using MS, we detected acetylation and methylation of H3 variant (H3.1) at K24 and K80 (Fig. 5, *B* and *C*) and Tables S2 and S3. Interestingly, posttranslation modification spectrum counts of H3.1-K24ac and H3.1-K80me were higher (*p* < 0.05) in the lungs of SU/Hx/Nx compared to SU/Nx G6PD^S188F^ but not WT rats (Fig. 5*D*). While there was a decreasing trend in posttranslation modification spectrum counts between SU/Nx G6PD^S188F^ and WT rats (Fig. 5*D*). Thus, acetylation and methylation of histones was increased in SU/Hx/Nx G6PD^S188F^ rats.

### SU/Hx/Nx increases right ventricle (RV) pressure and hypertrophy in WT but not G6PD^S188F^ rats

**Figure 6:**
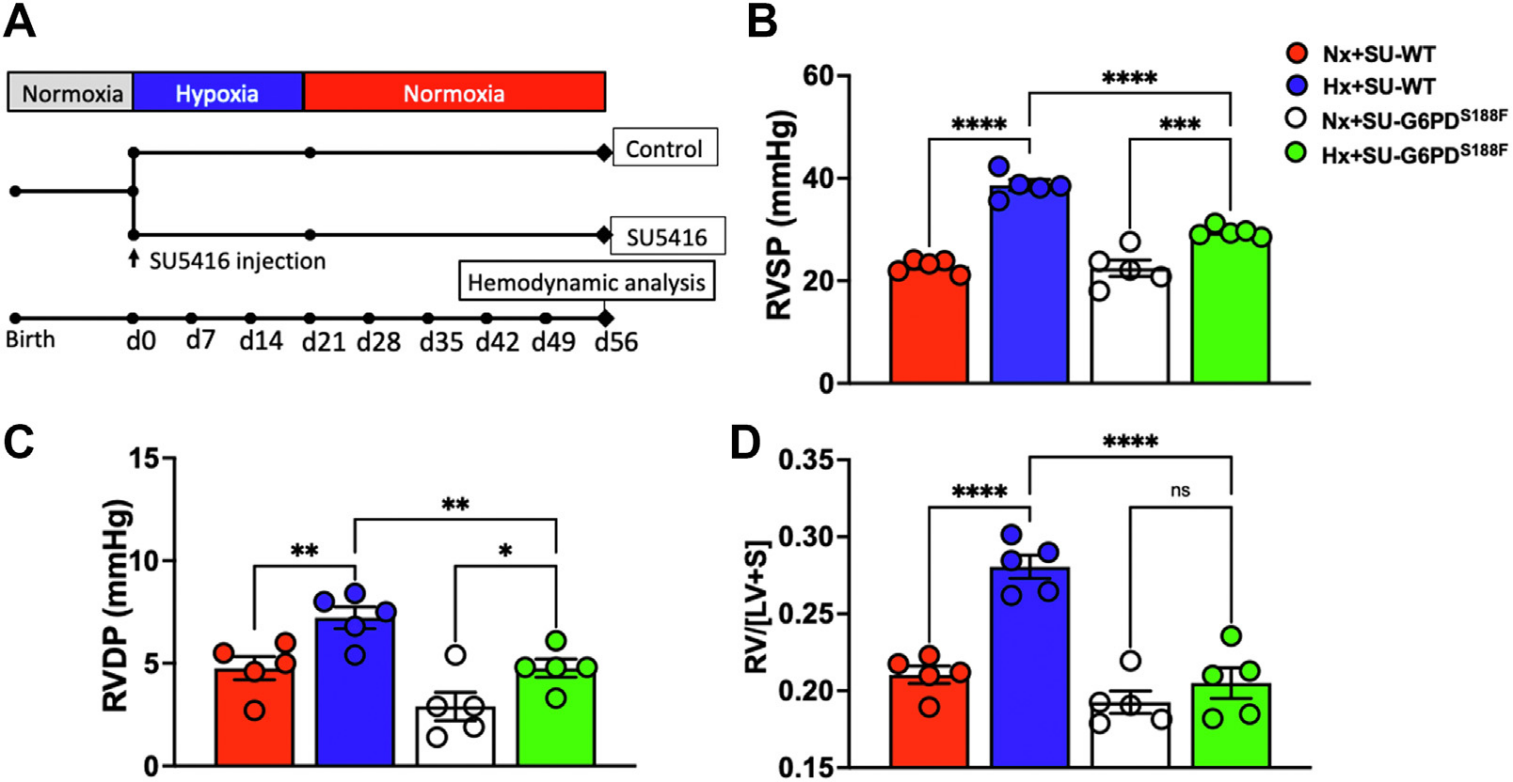
**Experimental schema and hemodynamic measurements in WTand Mediterranean G6PD**^**S188F**^**variant rats**. *A*, schematic showing that WT and G6PD^S188F^ rats injected with sugen5416 (20 mg/kg subcutaneously) were kept in normobaric hypoxia (10% O_2_) for 3 weeks (21 days) and then in normoxia (21% O_2_) for 5 weeks (35 days). Rats injected with sugen5416 (20 mg/kg subcutaneously) and kept under normoxia for 8 weeks (56 days) were used as controls. At the end of day 56, *right ventricular pressure* was measured through catheterization. *B*–*D*, *right ventricular* pressure (RVSP and RVDP) and hypertrophy in SU/Hx/Nx WT and G6PD^S188F^ rats. N (number of rats) = 5 in each group. Comparisons among all four groups were made using two-way ANOVA. ∗*p* < 0.05; ∗∗*p* < 0.005; ∗∗∗*p* < 0.0005; and ∗∗∗∗*p* < 0.0001. G6PD, glucose-6-phosphate dehydrogenase; RVDP, RV end diastolic pressure; RVSP, RV systolic pressure.

As in previous studies (6, 21), exposing WT rats to SU/Hx/Nx, as illustrated in the schematic in Figure 6*A*, increased RV systolic pressure (RVSP) by 69.9% (*p* < 0.05; Fig. 6*B*), RV end diastolic pressure (RVDP) by 52.1% (*p* < 0.05; Fig. 6*C*), and RV hypertrophy by 32.2% (*p* < 0.05; Fig. 6*D*). Next, we determined whether G6PD contributes to increase RV pressure and hypertrophy. In G6PD^S188F^ rats, SU/Hx/Nx raised RVSP by 25.1% (*p* < 0.05). Intriguingly, RVSP increased (44.8%) more in WT than G6PD^S188F^ rats. Moreover, while SU/Hx/Nx did not increase RVDP (Fig. 6*C*) or RV hypertrophy (Fig. 6*D*) as compared to SU/Nx in G6PD^S188F^ rats, it significantly increased (*p* < 0.05) in WT rats. The effects of SU/Hx/Nx on RVDP and RV hypertrophy were significantly greater in WT than G6PD^S188F^ rats.

## Discussion

Evidence that alteration of DNA and histone methylation or acetylation (epigenetic modifications) associate with the development of PH has started to accumulate (22). It has been proposed, for example, that altered methylation and acetylation marks on histones produce a persistent inflammatory phenotype in fibroblasts and an apoptosis-resistant and hyperproliferative phenotype of endothelial cells and SMCs from hypertensive patients and animal models (20). However, detailed information regarding the role of DNA methylation and aberrant gene/protein expression on the development and progression of PH remains to be determined.

Because methyltransferases and demethylases regulate the level of DNA and histone methylation and can thus alter gene transcription (1), we investigated DNMT expression and total activity in the lungs of SU/Hx/Nx rats, an experimental model of PH. While *Dnmt1*/DNMT1 and *Dnmt3b*/DNMT3B expression either increased or showed increasing trend in SU/Hx/Nx-WT rats, *Dnmt3a* and *Dnmt3b* expression was upregulated in Hx-WT rats. Consistently, methylation of H19ICR sequence, determined by MeDIP that linearly measures methylated cytosines (23), increased in lungs of SU/Hx/Nx-WT rats but not G6PD^S188F^ rats. Because DNMT1 and DNMT3B expression tend to increase in arteries and alveoli, we anticipate total differential methylation occurred in the lungs (including arteries, airway, and alveoli) contributed to the pathogenesis of pulmonary disease. Further, since expression of all three DNMT mRNA and total DNMT activity increases in leukocytes and in extracellular vesicles of scleroderma-associated and idiopathic PH patients (24), we propose that release of increased arterial and alveolar DNMT1 and DNMT3B in extracellular vesicles perhaps has paracrine-like action. One recent study suggests DNMT3B protein increases in lungs of congenital heart disease–associated PH patients and rats exposed to monocrotaline or Hx, and knockdown of *Dnmt3b* in rats exacerbates monocrotaline- and Hx-induced PH (25).

It is now well established that increased DNMT-catalyzed methylation of CpG islands within gene promoter silences expression of those genes. Altered DNA methylation in the lungs of hypoxic mice was previously reported to reduce expression of several genes encoding proteins implicated in the pathogenesis of PH (2). Other important gene (for example, *Sod2* and *Nos3*) promoters are hypermethylated in both adult and neonatal PH (14, 15). Consistently, *Sod2*, *Abra*, and *Serpine1* gene promoters were hypermethylated in SU/Hx/Nx-WT rats. Along the line of DNA methylation results, we found that expression of [1] *Nos3*, which encodes the endothelial nitric oxide synthase that generates vasodilatory nitric oxide (26), [2] *Sod2*, which encodes mitochondrial superoxide dismutase that scavenges superoxide anion produced in the respiratory chain (27), [3] *Abra*, which encodes actin-binding Rho-activating protein that regulates vascular smooth muscle phenotype (16), and [4] *Serpine1*, which encodes the prothrombotic protein PAI-1 (17), either decreased or showed decreasing trends in SU/Hx/Nx-WT rats. Conversely, NOS3 and SOD2 mRNA and protein either increased or showed increasing trend in SU/Hx/Nx-G6PD^S188F^ variant but not WT rats. DNA hypermethylation contributes to the development of vascular diseases (28, 29) and to right heart failure in PH (30). Our results suggest increased DNMT1, a maintenance methyltransferase, and DNMT3B, which elicits *de novo* genome-wide methylation, expression and activity methylates and suppresses genes encoding protective as well as malevolent proteins that, respectively, contribute to the pathogenesis of experimental PH and perhaps simultaneously reduce the severity of PH as seen before (25).

DNMT activity is driven by metabolites of one-carbon metabolism and the polyamine pathway (4, 12, 31). Each of those pathways is reprogrammed in various cell types residing in the pulmonary artery and lungs of PH patients and animal models (13). Further, polyamine pathway metabolite has been associated with the development of monocrotaline-induced PH (32). Because these metabolic pathways are subject to G6PD/NADPH-dependent regulation (2, 7, 33) and expression of SAHH/SAHH2 and MAT2A is increased or showed increasing trend in WT but not in G6PD^S188F^ variant rats, we assessed the activity of DNMT in the lungs of normoxic and SU/Hx/Nx rats. While there were no differences in the pulmonary DNMT activity in normoxic WT and G6PD^S188F^ rats, DNMT activity was higher in the lungs of SU/Hx/Nx than normoxic WT rats but not normoxic G6PD^S188F^ rats. This may be attributable to there being less dimethylglycine and spermine, metabolites of one carbon and polyamine pathway, which produces substrate required for DNMT activity and DNA methylation, in the vascular tissue of G6PD^S188F^ than WT rats (7) or pulmonary *Dnmt1*/DNMT1 and *Dnmt3b*/DNMT3B expression does not differ between SU/Hx/Nx and normoxic G6PD^S188F^ rats. Although the mechanism by which G6PD regulates expression of *Dnmts* remains unclear, our results demonstrate that G6PD, which regulates one-carbon metabolism and polyamine pathway (2, 33), controls the activity of metabolically driven DNMT enzymes. Undoubtedly, the loss-of-function G6PD^S188F^ variant prevented SU/Hx/Nx-induced increases in *Dnmt* expression/activity and consequentially hypomethylated and, at least partially, contributed to the increased expression of *Nos3* and *Sod2* genes and decreased methylation of *Abra* and *Serpine1* genes, in SU/Hx/Nx-G6PD^S188F^ rats as compared with SU/Hx/Nx-WT rats.

Linker histone1H1A, which is found to be more prevalent in euchromatin regions, and histone1H1E, which is abundant in heterochromatin regions, of the nucleus increased and decreased, respectively, in SU/Hx/Nx-G6PD^S188F^ rats. Since histone1H1E binds DNMT1 and DNMT3B and silencing of histone1H1E reduces DNA methylation (19), we suggest decreased histone1H1E expression likely contributed to DNA hypomethylation and increased transcription of *Nos3* and *Sod2* in SU/Hx/Nx-G6PD^S188F^ rats. Chromatin modifications (histone acetylation and methylation) are emerging contributors to gene regulation that persistently activate cell phenotypes in PH (20). Expression of histone deacetylases, which remove acetylation from histones, appears to increase in PH (34). We detected more acetylation of H3.1-K24 and methylation of H3.1-K80 in the lungs of SU/Hx/Nx-G6PD^S188F^ rats. This indicates that, in addition to mediating modification of DNA methylation, decreased G6PD activity played a role in increasing histone acetylation and methylation, which opens the chromatin structure and activates transcription. Along those lines, we recently found inhibition of G6PD activity or silencing *G6pd* expression reduces HDAC5 expression and HDAC activity in vascular tissue (18). Since methylation at Lys-80 is linked to gene activation (35, 36), we suggest along with DNA hypomethylation, increased H3.1-K80me in the lungs of G6PD^S188F^ rats, at least partially, contributed to prevent SU/Hx/Nx-induced decrease of proteins in the mitochondrial respiratory chain, cAMP-PKA–related signaling, and SERCA pump. Additionally, several proteins that were upregulated (*e*.*g*., ARRB1 and ITPR2) in SU/Hx/Nx as compared with normoxic WT rats remained unchanged in SU/Hx/Nx as compared with normoxic G6PD^S188F^ rats. Our proteomic approach confirmed proteins that are known to increase (*e*.*g*., glycolytic pathway proteins and complement proteins) or decrease (*e*.*g*., mitochondrial function proteins and SMC phenotype proteins) in PH and additionally identified changes that were hitherto unknown in the expression of proteins (cell signaling, serine proteases, RNA transcription and translation, and other pathways) in lungs of SU/Hx/Nx WT but not G6PD^S188F^ rats. Changes in the expression of those proteins from the control levels likely contribute to the pulmonary artery contraction and remodeling associated with PH (37, 38, 39, 40). What’s more, SU/Hx/Nx-induced increases in RVSP, RVDP, and RV hypertrophy were less in G6PD^S188F^ than WT rats. We therefore suggest that G6PD^S188F^ through reducing DNA methylation, augmenting histone acetylation and methylation, and a yet unknown mechanism modifies the epigenomes of genome-wide genes to balance expression of genes encoding beneficial and pathogenic proteins and make these rats less susceptible to SU/Hx/Nx-induced experimental PH.

PH is a heterogenous and complex disease. It exhibits a dysregulated metabolic program, thrombosis, inflammation, and fibrosis. PH also exhibits cancer-like physiognomies, and hence, some studies have characterized PH as cancer-like disease (37, 41). One of the hallmarks of cancer is alteration of DNA methylation, resulting in increased expression of oncogenes and/or decreased expression of tumor suppressor genes (42, 43). Increased methylation frequently silences DNA repair genes and miRs involved in cancer biology (44). As in cancer, differential DNA methylation is associated with PH in experimental models and humans (2, 3, 14, 15, 22, 30, 45). Our data, which are consistent with the recent findings (2, 3, 45), suggest DNMT-catalyzed DNA methylation appears to be a critical regulator of genes involved in the development and progression of PH pathology. While previous studies suggest G6PD^S188F^ variant makes no contribution to sickle cell anemia–associated PH, others suggest the frequency of G6PD mutation is higher among idiopathic PH patients than the general population (8, 9, 10). A more recent study shows G6PD-deficient mice spontaneously develop a PH phenotype (46), while these G6PD-deficient mice develop less Hx-induced PH (2). Our novel findings suggest that the loss-of-function Mediterranean G6PD^S188F^ variant, which has 60% to 80% less activity in lungs (Fig. 1*B*) but not protein expression and develop less systemic hypertension (7), antagonize DNMT-catalyzed DNA methylation, and pathogenic gene expression and, in the process, potentially reduce RV pressure overload/PH in SU/Hx/Nx rats.

## Experimental procedures

### Animal models and experimental protocols

All animal experiments were approved by the New York Medical College Animal Care and Use Committee, and all procedures conformed to the guidelines from the NIH Guide for the Care and Use of Laboratory Animals. To determine the expression and activity of DNA methylation writers and erasers in pulmonary hypertension samples, male Sprague–Dawley rats (350–750 g) were randomly divided into normoxia (Nx; 21% O_2_; control), hypoxia (Hx: 10% O_2_; control), sugen5416+normoxia (Nx+SU), and sugen5416+hypoxia+normoxia (SU/Hx/Nx) groups. Rats in the SU+Nx group were placed in an ambient (21% O_2_) environment. SU/Hx/Nx rats received one subcutaneous injection of SU (20 mg/kg) and were then exposed to 3 weeks of Hx (10% O_2_) followed by 5 weeks of Nx (21% O_2_), as described previously (6, 21). SU/Hx/Nx rats are preclinical models of PH (47). In addition, to determine the role of G6PD in the pathogenesis of SU/Hx/Nx-induced experimental PH in rats, we used loss-of-function Mediterranean G6PD^S188F^ variant rats (350–750 g) recently generated in our laboratory and their WT littermates (7). Hemodynamic measurements were made, lungs were harvested, and blood samples were collected. Data analysis was performed in blinded fashion.

### Hemodynamic measurements

All rats were anesthetized through inhalation of isoflurane (isoflurane, USP; 1-chloro-2,2,2-trifluoroethyl difluoro methyl ether; induced at 3% and maintained at 1.5%) and placed on a heated table. Closed-chest cardiac catheterization was performed using an MPVS Ultra Single Segment Pressure-Volume Unit (Millar Instruments) in combination with a cardiac catheter. RVSP and RVDP were measured after catheterization of the right ventricle *via* the right external jugular vein using a Millar Mikro-Tip catheter (Model SPR-671, tip size of 1.4F, Millar Instruments). Once steady-state hemodynamics were achieved, RV ventricular pressure-volume (P-V) loops were recorded and analyzed using LabChart 8 software (ADInstruments).

### Hematocrit measurements

After hemodynamic measurements were completed, blood was collected from the cardiac chambers into a heparinized syringe. The heparinized blood was placed in capillary tubes, and hematocrit (%) was calculated as the length of the erythrocyte layer divided by the length of the entire blood sample.

### Assessment of RV hypertrophy

Following the cardiac catheterization, the animals were euthanized by cervical dislocation, whole hearts were excised, and the RV free wall and left ventricle, including the ventricular septum (S), were separated and weighed independently. Fulton’s index (right ventricle/left ventricle + S ratio) was calculated as an index of RV hypertrophy.

### Measurement of DNMT activity

DNMT activity was measured using kits from Epigentek. DNMT activity was measured in lysates prepared from lungs collected from rats.

### Quantitative RT-PCR

RT-PCR was used to analyze mRNA expression. Briefly, total RNA was extracted from lungs and human leukocytes using a Qiagen miRNEasy kit (catalog no.: # 217004). The quality and concentration of the input RNA were measured on the Synergy HT Take3 Microplate Reader (BioTek) and complementary DNA was prepared using SuperScript IV. VILO Master Mix (catalog no.: # 11756500, Invitrogen) for mRNA. Quantitative PCR (qPCR) was performed in duplicate using TaqMan Fast Advanced Master Mix (catalog no.: # 44-445-57) for mRNA using a Mx3000p Real-Time PCR System (Stratagene). The primers for the qPCR were purchased from Thermo Fisher Scientific/TaqMan. mRNA levels were normalized to internal control *Tuba1a*, and relative mRNA expression was reported.

### MS-PCR

DNA methylation was analyzed using MS-PCR. Briefly, DNA was isolated from lungs (5 μg) using a FitAmp General Tissue Section DNA Isolation Kit (EpiGentek catalog no.: # Kit P-1003), after which the collected DNA was eluted in Tris-EDTA buffer in a total volume of 30 μl. The DNA was then quantified using a fluorescence quantification method, and 150 ng samples of the DNA were used to perform bisulfite conversion using the Methylamp DNA Bisulfite Conversion Kit (EpiGentek catalog no.: # P-1001). The conversion efficiency of bisulfite-treated DNA was determined with RT-PCR using two primer pairs and control DNA. The first primer pair was against bisulfite-converted DNA (β-actin), while the second was against unconverted DNA (GAPDH) in the same bisulfite-treated DNA sample. The DNA was shown to be >98% converted. qPCR was performed in duplicate using 1-μl aliquots of modified DNA and gene-specific primers designed rat genes. Data analysis was performed using qPCR Ct values to calculate the percentage MI for each target region: % MI = [1 – (2^*(Ct M-Ct uM)*^/2^*(Ct M-Ct uM+1)*^*)*] × 100%. The MI measures the degree of methylation at a specific gene site; a higher MI indicates a higher degree of methylation. The MI for fully methylated DNA is defined as 100%. A relative comparison of the samples can be made based on the MI.

### MeDIP

In addition to MS-PCR, we performed MeDIP-PCR to determine total DNA methylation status. We used quick EpiQuik Tissue Methylated DNA immunoprecipitation kit (catalog no.: # P-2020) from EpiGentek followed by PCR, according to the manufacturer’s protocol, and determined methylation of DNA.

### Western blotting

Rat lungs were homogenized, and cells were lysed in lysis buffer (50 mmol/L Tris–HCl pH 7.4, 150 mmol/l NaCl, 0.5% nonidet P-40) containing EDTA-free complete protease and phosphatase inhibitor (Sigma–Aldrich/Roche). Protein levels were measured using Bradford assays (Bio-Rad), after which 35 μg samples were run on SDS-PAGE gels, transferred to nitrocellulose membranes, blocked with either 5% bovine serum albumin or 4% milk, and subsequently incubated with primary and secondary antibodies and detected using SuperSignal West Pico Chemiluminescent Substrate (catalog no.: # 34080, Thermo Fisher Scientific) on iBright. Protein levels were determined through densitometric analysis using ImageJ software (NIH). The following previously validated primary antibodies were used: DNMT1 (Cell Signaling), DNMT3A (Cell Signaling), DNMT3B (Epigentek), MAT2A (Novus Biologicals), SAHH (Santacruz), NOS3 (Novus Biologicals), SOD2 (Novus Biologicals), SERPINE1 (Novus Biologicals), and ABRA (Novus biologicals). ACTB (Cell Signaling) was used as a loading control.

### Immunohistochemistry

The superior and inferior lobe of the left lung was inflated with 1% neutral buffered formalin in 0.5% agarose at 20 cm H_2_O pressure and fixed in 10% neutral buffered formalin as described previously (6). The fixed lung was blocked and embedded in paraffin, after which 5-μm sections were cut and probed for DNMT1 (Novus), DNMT3A (Epigentek), and DNMT3B (Thermo Fisher) by immunohistochemistry and images were acquired on Nikon Eclipse 90-i microscope.

### MS-based proteomics

#### Sample preparation

Frozen samples were powdered in liquid nitrogen using a ceramic mortar and pestle and lyophilized. Aliquots of tissue (approximately 2 mg of each) were homogenized in freshly prepared high-salt buffer (50 mM Tris–HCl, 3 M NaCl, 25 mM EDTA, 0.25% w/v CHAPS, pH 7.5) containing 1× protease inhibitor (Halt Protease Inhibitor, Thermo Scientific) at a concentration of 10 mg/ml. Homogenization was accomplished in a bead beater (Bullet Blender Storm 24, Next Advance, 1 mm glass beads) for 3 min at 4 °C. Samples were then spun for 20 min at 18,000*g* at 4 °C, after which the supernatant removed and stored as *Fraction 1*. A fresh aliquot of high-salt buffer was added to the remaining pellet at 10 mg/ml of the starting weight, vortexed at 4 °C for 15 min, and spun for 15 min. That supernatant was removed and stored as *Fraction 2.* This high-salt extraction was repeated once more to generate *Fraction 3*, after which freshly prepared guanidine extraction buffer (6 M guanidinium chloride adjusted to pH 9.0 with NaOH) was added at 10 mg/ml and vortexed for 1 h at room temperature (RT). The samples were then spun for 15 min, after which the supernatant was removed and stored as *Fraction 4. Fractions 1*, *2*, and *3* were combined, and all fractions were stored at −20 °C until further analysis.

#### Sample digestion

The samples were digested according to the filter-aided sample preparation (FASP) protocol using a 10-kDa molecular weight cutoff filter. In brief, 200-μl samples were mixed in the filter unit with 8 M urea, 0.1 M ammonium bicarbonate (AB) pH 8.0, and centrifuged at 14,000*g* for 15 min. The proteins were reduced with 10 mM DTT for 30 min at RT, centrifuged, and alkylated with 55 mM iodoacetamide for 30 min at RT in the dark. Following centrifugation, samples were washed three times with urea solution and three times with 50 mM AB, pH 8.0. Protein digestion was carried out with sequencing grade modified trypsin (Promega) at 1/50 protease/protein (wt/wt) ratio overnight at 37 °C. Peptides were recovered from the filter using 50 mM AB. Samples were dried in a Speed-Vac and then desalted and concentrated on a Thermo Scientific Pierce C18 Tip.

#### MS

Twenty microliters of each sample were loaded onto individual Evotips for desalting and then washed with 20 μl 0.1% formic acid followed by addition of 100 μl of storage solvent (0.1% formic acid) to keep the Evotips wet until analysis. An Evosep One system (Evosep, Odense, Denmark) was used to separate the peptides on a Pepsep column, (150 μm inner diameter, 15 cm) packed with ReproSil C18 1.9 μm, 120 Å resin. The system was coupled to a timsTOF Pro mass spectrometer (Bruker Daltonics) *via* a nano–electrospray ion source (Captive Spray, Bruker Daltonics). The mass spectrometer was operated in PASEF mode. The ramp time was set to 100 ms and 10 PASEF MS/MS scans per topN acquisition cycle were acquired. MS and MS/MS spectra were recorded from *m/z* 100 to 1700. The ion mobility was scanned from 0.7 to 1.50 Vs/cm^2^. Precursors for data-dependent acquisition were isolated within  ± 1  Th and fragmented with ion mobility-dependent collision energy, which was linearly increased from 20 to 59 eV in positive mode. Low-abundance precursor ions with an intensity above a threshold of 500 counts but below a target value of 20,000 counts were repeatedly scheduled and otherwise dynamically excluded for 0.4 min.

#### Database searching and protein identification

MS/MS spectra were extracted from raw data files and converted into .mgf files using MS Convert (ProteoWizard, Ver. 3.0). Peptide spectral matching was performed with Mascot (Ver. 2.5) against the Uniprot rat database. Mass tolerances were ±15 ppm for parent ions and ± 0.4 Da for fragmented ions. Trypsin specificity was used, allowing for one missed cleavage. Met oxidation, monomethylation and dimethylation (KR), trimethylation (K), protein N-terminal acetylation, acetylation (K), and peptide N-terminal pyroglutamic acid formation were set as variable modifications with Cys carbamidomethylation set as a fixed modification. Scaffold (version 4.8, Proteome Software) was used to validate MS/MS-based peptide and protein identifications. Peptide identifications were accepted, if they could be established at greater than 95.0% probability as specified by the Peptide Prophet algorithm. Protein identifications were accepted if they could be established at greater than 99.0% probability and contained at least two identified unique peptides.

### Statistical analysis

Statistical analysis was performed using GraphPad Prism 9 software (GraphPad Software Inc). Values are presented as mean ± standard error. Statistical comparisons of samples were made for two groups with Student’s *t* test. To make comparisons among more than two groups, two-way ANOVA followed by Sidak’s post hoc test for multiple comparisons was used. Values of *p* < 0.05 were considered significant.

## Data availability

All data are contained within the article and will be available upon written request to the corresponding author.

## Supporting information

This article contains supporting information.

## Conflict of interest

The authors declare that they have no conflicts of interest with the contents of this article.
