# Supplementary material for: Mediterranean G6PD variant mitigates expression of DNA methyltransferases and right heart pressure in experimental model of pulmonary hypertension
Source: J Biol Chem. 2022 Nov 11;298(12):102691. doi: 10.1016/j.jbc.2022.102691 (PMC9731845; doi:10.1016/j.jbc.2022.102691)
Supplement: Supplemental table [file mmc1.docx]

| **Mediterranean G6PD variant mitigates expression of DNA methyltransferases and right heart pressure in experimental model of pulmonary hypertension**  Christina Jacob,^1^ Atsushi Kitagawa,^1^ Monika Dzieciatkowska,^2^ Angelo D’Alessandro,^2,^ Christina Signoretti,^1^ Shakib Hossain,^1^ Aaditya Gupte,^1^ Catherine A. D’Addario,^1^ Rakhee Gupte,^1^ and Sachin A. Gupte^1*^  ^1^Department of Pharmacology, New York Medical College, Valhalla, NY, USA  ^2^Department of Biochemistry & Molecular Genetics, School of Medicine, University of Colorado Denver - Anschutz Medical Campus, Aurora, CO, USA  **Short Title**: DNMTs and G6PD in PH  *Address Correspondence to:  Sachin A. Gupte, MD, PhD  Professor, Department of Pharmacology  New York Medical College  Valhalla, NY 10595  Tel: 914-594-3937  **Table 1: Mass spec results** |
| --- |
|  |
| Protein Grouping Strategy: Experiment-wide grouping with binary peptide-protein weights |
| Peptide Thresholds: 95.0% minimum |
| Protein Thresholds: 99.0% minimum and 2 peptides minimum |
| Peptide FDR: 0.5% (Prophet) |
| Protein FDR: 0.0% (Prophet) |
| GO Annotation Source(s): |
| Pathway Annotation Source(s): |
| Alternate ID Source(s): FASTA:UniProt/Swiss-Prot (UniProtKB), FASTA:UniProt/Swiss-Prot (UniProtKB), FASTA |
|  |

|  |  |  |  |
| --- | --- | --- | --- |
| ***Accession Number*** | ***Alternate ID*** | ***Molecular Weight*** | ***Taxonomy*** |
| ALBU_RAT | Alb | 69 kDa | Rattus norvegicus |
| HBA_RAT | Hba1 | 15 kDa | Rattus norvegicus |
| HBB1_RAT | Hbb | 16 kDa | Rattus norvegicus |
| ACTB_RAT | Actb | 42 kDa | Rattus norvegicus |
| A1I3_RAT | A1i3 | 164 kDa | Rattus norvegicus |
| SPTN1_RAT | Sptan1 | 285 kDa | Rattus norvegicus |
| MYH9_RAT | Myh9 | 226 kDa | Rattus norvegicus |
| A1M_RAT | A1m | 167 kDa | Rattus norvegicus |
| TRFE_RAT | Tf | 76 kDa | Rattus norvegicus |
| CO3_RAT | C3 | 186 kDa | Rattus norvegicus |
| EF1A1_RAT | Eef1a1 | 50 kDa | Rattus norvegicus |
| CLH1_RAT | Cltc | 192 kDa | Rattus norvegicus |
| DYHC1_RAT | Dync1h1 | 532 kDa | Rattus norvegicus |
| ACTA_RAT | Acta2 | 42 kDa | Rattus norvegicus |
| PRDX6_RAT | Prdx6 | 25 kDa | Rattus norvegicus |
| MOES_RAT | Msn | 68 kDa | Rattus norvegicus |
| TBB4B_RAT | Tubb4b | 50 kDa | Rattus norvegicus |
| DPYL2_RAT | Dpysl2 | 62 kDa | Rattus norvegicus |
| EHD2_RAT | Ehd2 | 61 kDa | Rattus norvegicus |
| MYH10_RAT | Myh10 | 229 kDa | Rattus norvegicus |
| TBA1B_RAT | Tuba1b | 50 kDa | Rattus norvegicus |
| MUG1_RAT | Mug1 | 165 kDa | Rattus norvegicus |
| FAS_RAT | Fasn | 273 kDa | Rattus norvegicus |
| G3P_RAT | Gapdh | 36 kDa | Rattus norvegicus |
| SBP1_RAT | Selenbp1 | 53 kDa | Rattus norvegicus |
| MYH11_RAT | Myh11 | 152 kDa | Rattus norvegicus |
| MACF1_RAT | Macf1 | 620 kDa | Rattus norvegicus |
| S14L3_RAT | Sec14l3 | 46 kDa | Rattus norvegicus |
| HS90B_RAT | Hsp90ab1 | 83 kDa | Rattus norvegicus |
| CAH2_RAT | Ca2 | 29 kDa | Rattus norvegicus |
| VIME_RAT | Vim | 54 kDa | Rattus norvegicus |
| VINC_RAT | Vcl | 117 kDa | Rattus norvegicus |
| PLEC_RAT | Plec | 534 kDa | Rattus norvegicus |

|  |
| --- |

| ACTN1_RAT | Actn1 | 103 kDa | Rattus norvegicus |
| --- | --- | --- | --- |
| ACE_RAT | Ace | 151 kDa | Rattus norvegicus |
| EHD1_RAT | Ehd1 | 61 kDa | Rattus norvegicus |
| HSP7C_RAT | Hspa8 | 71 kDa | Rattus norvegicus |
| RAGE_RAT | Ager | 43 kDa | Rattus norvegicus |
| GUAD_RAT | Gda | 51 kDa | Rattus norvegicus |
| CES1D_RAT | Ces1d | 62 kDa | Rattus norvegicus |
| CLIC5_RAT | Clic5 | 28 kDa | Rattus norvegicus |
| GELS_RAT | Gsn | 86 kDa | Rattus norvegicus |
| ANXA3_RAT | Anxa3 | 36 kDa | Rattus norvegicus |
| ATPB_RAT | Atp5f1b | 56 kDa | Rattus norvegicus |
| HEMO_RAT | Hpx | 51 kDa | Rattus norvegicus |
| ANXA2_RAT | Anxa2 | 39 kDa | Rattus norvegicus |
| MYO1C_RAT | Myo1c | 120 kDa | Rattus norvegicus |
| PNPH_RAT | Pnp | 32 kDa | Rattus norvegicus |
| AL1A1_RAT | Aldh1a1 | 54 kDa | Rattus norvegicus |
| ENOA_RAT | Eno1 | 47 kDa | Rattus norvegicus |
| COF1_RAT | Cfl1 | 19 kDa | Rattus norvegicus |
| AK1CA_RAT | Akr1c15 | 37 kDa | Rattus norvegicus |
| ROA2_RAT | Hnrnpa2b1 | 37 kDa | Rattus norvegicus |
| ACTN4_RAT | Actn4 | 105 kDa | Rattus norvegicus |
| ANXA5_RAT | Anxa5 | 36 kDa | Rattus norvegicus |
| ANXA1_RAT | Anxa1 | 39 kDa | Rattus norvegicus |
| IGG2A_RAT | Igg-2a | 35 kDa | Rattus norvegicus |
| ENPL_RAT | Hsp90b1 | 93 kDa | Rattus norvegicus |
| GDIB_RAT | Gdi2 | 51 kDa | Rattus norvegicus |
| CATA_RAT | Cat | 60 kDa | Rattus norvegicus |
| 1433E_RAT | Ywhae | 29 kDa | Rattus norvegicus |
| CERU_RAT | Cp | 121 kDa | Rattus norvegicus |
| ANXA6_RAT | Anxa6 | 76 kDa | Rattus norvegicus |
| DPP4_RAT | Dpp4 | 88 kDa | Rattus norvegicus |
| DEST_RAT | Dstn | 19 kDa | Rattus norvegicus |
| CAN2_RAT | Capn2 | 80 kDa | Rattus norvegicus |
| BCAM_RAT | Bcam | 68 kDa | Rattus norvegicus |
| KPYM_RAT | Pkm | 58 kDa | Rattus norvegicus |
| ITB1_RAT | Itgb1 | 88 kDa | Rattus norvegicus |
| CNDP2_RAT | Cndp2 | 53 kDa | Rattus norvegicus |
| K1C19_RAT | Krt19 | 45 kDa | Rattus norvegicus |
| H4_RAT | Hist1h4b | 11 kDa | Rattus norvegicus |
| MYH6_RAT | Myh6 | 224 kDa | Rattus norvegicus |
| TERA_RAT | Vcp | 89 kDa | Rattus norvegicus |
| AGRIN_RAT | Agrn | 209 kDa | Rattus norvegicus |
| SPA3K_RAT | Serpina3k | 47 kDa | Rattus norvegicus |
| TKT_RAT | Tkt | 68 kDa | Rattus norvegicus |
| TBB5_RAT | Tubb5 | 50 kDa | Rattus norvegicus |
| CAH1_RAT | Ca1 | 28 kDa | Rattus norvegicus |
| H2B1_RAT |  | 14 kDa | Rattus norvegicus |

| PLST_RAT | Pls3 | 71 kDa | Rattus norvegicus |
| --- | --- | --- | --- |
| UBA1_RAT | Uba1 | 118 kDa | Rattus norvegicus |
| CALR_RAT | Calr | 48 kDa | Rattus norvegicus |
| PRDX2_RAT | Prdx2 | 22 kDa | Rattus norvegicus |
| ROA3_RAT | Hnrnpa3 | 40 kDa | Rattus norvegicus |
| KCRB_RAT | Ckb | 43 kDa | Rattus norvegicus |
| CAVN1_RAT | Cavin1 | 44 kDa | Rattus norvegicus |
| ACLY_RAT | Acly | 121 kDa | Rattus norvegicus |
| BIP_RAT | Hspa5 | 72 kDa | Rattus norvegicus |
| DPEP1_RAT | Dpep1 | 46 kDa | Rattus norvegicus |
| EF2_RAT | Eef2 | 95 kDa | Rattus norvegicus |
| CO4_RAT | C4 | 192 kDa | Rattus norvegicus |
| PDIA3_RAT | Pdia3 | 57 kDa | Rattus norvegicus |
| CRP_RAT | Crp | 25 kDa | Rattus norvegicus |
| CALX_RAT | Canx | 67 kDa | Rattus norvegicus |
| ODO1_RAT | Ogdh | 116 kDa | Rattus norvegicus |
| HNRPL_RAT | Hnrnpl | 68 kDa | Rattus norvegicus |
| PPIA_RAT | Ppia | 18 kDa | Rattus norvegicus |
| GNAI2_RAT | Gnai2 | 41 kDa | Rattus norvegicus |
| PROF1_RAT | Pfn1 | 15 kDa | Rattus norvegicus |
| A1AT_RAT | Serpina1 | 46 kDa | Rattus norvegicus |
| CH60_RAT | Hspd1 | 61 kDa | Rattus norvegicus |
| HS90A_RAT | Hsp90aa1 | 85 kDa | Rattus norvegicus |
| HSP74_RAT | Hspa4 | 94 kDa | Rattus norvegicus |
| PLMN_RAT | Plg | 91 kDa | Rattus norvegicus |
| LMNA_RAT | Lmna | 74 kDa | Rattus norvegicus |
| WDR1_RAT | Wdr1 | 66 kDa | Rattus norvegicus |
| ALDH2_RAT | Aldh2 | 56 kDa | Rattus norvegicus |
| GBB1_RAT | Gnb1 | 37 kDa | Rattus norvegicus |
| VTDB_RAT | Gc | 54 kDa | Rattus norvegicus |
| PDIA1_RAT | P4hb | 57 kDa | Rattus norvegicus |
| CP4B1_RAT | Cyp4b1 | 59 kDa | Rattus norvegicus |
| MVP_RAT | Mvp | 96 kDa | Rattus norvegicus |
| G6PI_RAT | Gpi | 63 kDa | Rattus norvegicus |
| GDIR1_RAT | Arhgdia | 23 kDa | Rattus norvegicus |
| TCPB_RAT | Cct2 | 57 kDa | Rattus norvegicus |
| NB5R3_RAT | Cyb5r3 | 34 kDa | Rattus norvegicus |
| AKAP2_RAT | Akap2 | 96 kDa | Rattus norvegicus |
| PYGB_RAT | Pygb | 96 kDa | Rattus norvegicus |
| ACON_RAT | Aco2 | 85 kDa | Rattus norvegicus |
| EZRI_RAT | Ezr | 69 kDa | Rattus norvegicus |
| VAT1_RAT | Vat1 | 43 kDa | Rattus norvegicus |
| MYO1D_RAT | Myo1d | 116 kDa | Rattus norvegicus |
| GDIA_RAT | Gdi1 | 51 kDa | Rattus norvegicus |
| CP2B1_RAT | Cyp2b1 | 56 kDa | Rattus norvegicus |
| K2C8_RAT | Krt8 | 54 kDa | Rattus norvegicus |
| XDH_RAT | Xdh | 146 kDa | Rattus norvegicus |

| KNT2_RAT |  | 48 kDa | Rattus norvegicus |
| --- | --- | --- | --- |
| ALDOA_RAT | Aldoa | 39 kDa | Rattus norvegicus |
| FLNC_RAT | Flnc | 291 kDa | Rattus norvegicus |
| XPP2_RAT | Xpnpep2 | 76 kDa | Rattus norvegicus |
| PGK1_RAT | Pgk1 | 45 kDa | Rattus norvegicus |
| ANXA4_RAT | Anxa4 | 36 kDa | Rattus norvegicus |
| ZO1_RAT | Tjp1 | 197 kDa | Rattus norvegicus |
| APOA1_RAT | Apoa1 | 30 kDa | Rattus norvegicus |
| TPM3_RAT | Tpm3 | 29 kDa | Rattus norvegicus |
| NCPR_RAT | Por | 77 kDa | Rattus norvegicus |
| IDHP_RAT | Idh2 | 51 kDa | Rattus norvegicus |
| ATPA_RAT | Atp5f1a | 60 kDa | Rattus norvegicus |
| TPM2_RAT | Tpm2 | 33 kDa | Rattus norvegicus |
| IMB1_RAT | Kpnb1 | 97 kDa | Rattus norvegicus |
| PTBP1_RAT | Ptbp1 | 59 kDa | Rattus norvegicus |
| SAHH_RAT | Ahcy | 48 kDa | Rattus norvegicus |
| B3AT_RAT | Slc4a1 | 103 kDa | Rattus norvegicus |
| CAV1_RAT | Cav1 | 21 kDa | Rattus norvegicus |
| IGG2B_RAT | Igh-1a | 36 kDa | Rattus norvegicus |
| IF5A1_RAT | Eif5a | 17 kDa | Rattus norvegicus |
| SEPT7_RAT | 7-Sep | 51 kDa | Rattus norvegicus |
| H14_RAT | Hist1h1e | 22 kDa | Rattus norvegicus |
| MPRIP_RAT | Mprip | 117 kDa | Rattus norvegicus |
| CAP1_RAT | Cap1 | 52 kDa | Rattus norvegicus |
| GSTM2_RAT | Gstm2 | 26 kDa | Rattus norvegicus |
| FETUA_RAT | Ahsg | 38 kDa | Rattus norvegicus |
| PRELP_RAT | Prelp | 43 kDa | Rattus norvegicus |
| ESYT1_RAT | Esyt1 | 121 kDa | Rattus norvegicus |
| FIBB_RAT | Fgb | 54 kDa | Rattus norvegicus |
| LDHA_RAT | Ldha | 36 kDa | Rattus norvegicus |
| SPA3L_RAT | Serpina3l | 46 kDa | Rattus norvegicus |
| MYO1B_RAT | Myo1b | 132 kDa | Rattus norvegicus |
| MRLCA_RAT | Rlc-a | 20 kDa | Rattus norvegicus |
| GSTP1_RAT | Gstp1 | 23 kDa | Rattus norvegicus |
| DYN2_RAT | Dnm2 | 98 kDa | Rattus norvegicus |
| THIO_RAT | Txn | 12 kDa | Rattus norvegicus |
| PUR9_RAT | Atic | 64 kDa | Rattus norvegicus |
| TAGL_RAT | Tagln | 23 kDa | Rattus norvegicus |
| ARP3_RAT | Actr3 | 47 kDa | Rattus norvegicus |
| DHE3_RAT | Glud1 | 61 kDa | Rattus norvegicus |
| CAZA1_RAT | Capza1 | 33 kDa | Rattus norvegicus |
| H2A1C_RAT (+1) |  | 14 kDa | Rattus norvegicus |
| SND1_RAT | Snd1 | 102 kDa | Rattus norvegicus |
| THRB_RAT | F2 | 70 kDa | Rattus norvegicus |
| CAND1_RAT | Cand1 | 136 kDa | Rattus norvegicus |
| TAGL2_RAT | Tagln2 | 22 kDa | Rattus norvegicus |
| UBR4_RAT | Ubr4 | 574 kDa | Rattus norvegicus |

| HMGB1_RAT | Hmgb1 | 25 kDa | Rattus norvegicus |
| --- | --- | --- | --- |
| PSME1_RAT | Psme1 | 29 kDa | Rattus norvegicus |
| DC1I2_RAT | Dync1i2 | 71 kDa | Rattus norvegicus |
| AP2A2_RAT | Ap2a2 | 104 kDa | Rattus norvegicus |
| THIL_RAT | Acat1 | 45 kDa | Rattus norvegicus |
| AP2B1_RAT | Ap2b1 | 105 kDa | Rattus norvegicus |
| FIBG_RAT | Fgg | 51 kDa | Rattus norvegicus |
| PGAM1_RAT | Pgam1 | 29 kDa | Rattus norvegicus |
| ROA1_RAT | Hnrnpa1 | 34 kDa | Rattus norvegicus |
| PIGR_RAT | Pigr | 85 kDa | Rattus norvegicus |
| PGS1_RAT | Bgn | 42 kDa | Rattus norvegicus |
| CAPZB_RAT | Capzb | 31 kDa | Rattus norvegicus |
| CSRP1_RAT | Csrp1 | 21 kDa | Rattus norvegicus |
| U520_RAT | Snrnp200 | 245 kDa | Rattus norvegicus |
| KACB_RAT |  | 12 kDa | Rattus norvegicus |
| PEBP1_RAT | Pebp1 | 21 kDa | Rattus norvegicus |
| LEG1_RAT | Lgals1 | 15 kDa | Rattus norvegicus |
| LPP_RAT | Lpp | 68 kDa | Rattus norvegicus |
| RAP1B_RAT | Rap1b | 21 kDa | Rattus norvegicus |
| MYL9_RAT | Myl9 | 20 kDa | Rattus norvegicus |
| AT2B1_RAT | Atp2b1 | 135 kDa | Rattus norvegicus |
| GCYB1_RAT | Gucy1b1 | 70 kDa | Rattus norvegicus |
| HS71A_RAT (+1) | Hspa1a | 70 kDa | Rattus norvegicus |
| AMPN_RAT | Anpep | 109 kDa | Rattus norvegicus |
| TCPE_RAT | Cct5 | 60 kDa | Rattus norvegicus |
| CPNS1_RAT | Capns1 | 29 kDa | Rattus norvegicus |
| PP1B_RAT | Ppp1cb | 37 kDa | Rattus norvegicus |
| AT2A2_RAT | Atp2a2 | 115 kDa | Rattus norvegicus |
| ITIH3_RAT | Itih3 | 99 kDa | Rattus norvegicus |
| RINI_RAT | Rnh1 | 50 kDa | Rattus norvegicus |
| STAT3_RAT | Stat3 | 88 kDa | Rattus norvegicus |
| UGGG1_RAT | Uggt1 | 176 kDa | Rattus norvegicus |
| CSPG4_RAT | Cspg4 | 252 kDa | Rattus norvegicus |
| TCPG_RAT | Cct3 | 61 kDa | Rattus norvegicus |
| COPB_RAT | Copb1 | 107 kDa | Rattus norvegicus |
| MUC18_RAT | Mcam | 71 kDa | Rattus norvegicus |
| HSPB1_RAT | Hspb1 | 23 kDa | Rattus norvegicus |
| PSMD2_RAT | Psmd2 | 100 kDa | Rattus norvegicus |
| 3HIDH_RAT | Hibadh | 35 kDa | Rattus norvegicus |
| AFAM_RAT | Afm | 69 kDa | Rattus norvegicus |
| ILK_RAT | Ilk | 51 kDa | Rattus norvegicus |
| DIAP1_RAT | Diaph1 | 140 kDa | Rattus norvegicus |
| DCTN1_RAT | Dctn1 | 142 kDa | Rattus norvegicus |
| LIS1_RAT | Pafah1b1 | 47 kDa | Rattus norvegicus |
| MYL6_RAT | Myl6 | 17 kDa | Rattus norvegicus |
| MDHM_RAT | Mdh2 | 36 kDa | Rattus norvegicus |
| HNRPU_RAT | Hnrnpu | 88 kDa | Rattus norvegicus |

| DPP3_RAT | Dpp3 | 83 kDa | Rattus norvegicus |
| --- | --- | --- | --- |
| H33_RAT | H3f3b | 15 kDa | Rattus norvegicus |
| NCKP1_RAT | Nckap1 | 129 kDa | Rattus norvegicus |
| MYADM_RAT | Myadm | 35 kDa | Rattus norvegicus |
| PRAX_RAT | Prx | 146 kDa | Rattus norvegicus |
| SERPH_RAT | Serpinh1 | 47 kDa | Rattus norvegicus |
| TCPA_RAT | Tcp1 | 60 kDa | Rattus norvegicus |
| HEM2_RAT | Alad | 36 kDa | Rattus norvegicus |
| 1433Z_RAT | Ywhaz | 28 kDa | Rattus norvegicus |
| ECHA_RAT | Hadha | 83 kDa | Rattus norvegicus |
| HRG_RAT | Hrg | 59 kDa | Rattus norvegicus |
| GSTA3_RAT | Gsta3 | 25 kDa | Rattus norvegicus |
| FKB1A_RAT | Fkbp1a | 12 kDa | Rattus norvegicus |
| PFKAP_RAT | Pfkp | 86 kDa | Rattus norvegicus |
| RIPR2_RAT | Ripor2 | 145 kDa | Rattus norvegicus |
| PRDX1_RAT | Prdx1 | 22 kDa | Rattus norvegicus |
| AMPL_RAT | Lap3 | 56 kDa | Rattus norvegicus |
| TPM4_RAT | Tpm4 | 29 kDa | Rattus norvegicus |
| MCPT1_RAT | Mcpt1 | 29 kDa | Rattus norvegicus |
| GBB2_RAT | Gnb2 | 37 kDa | Rattus norvegicus |
| AOC3_RAT | Aoc3 | 85 kDa | Rattus norvegicus |
| PARK7_RAT | Park7 | 20 kDa | Rattus norvegicus |
| MARCS_RAT | Marcks | 30 kDa | Rattus norvegicus |
| RAB1A_RAT | Rab1A | 23 kDa | Rattus norvegicus |
| PON3_RAT | Pon3 | 39 kDa | Rattus norvegicus |
| HNRPM_RAT | Hnrnpm | 74 kDa | Rattus norvegicus |
| IDHC_RAT | Idh1 | 47 kDa | Rattus norvegicus |
| 1433T_RAT | Ywhaq | 28 kDa | Rattus norvegicus |
| HBB2_RAT |  | 16 kDa | Rattus norvegicus |
| CLIC4_RAT | Clic4 | 29 kDa | Rattus norvegicus |
| COPB2_RAT | Copb2 | 103 kDa | Rattus norvegicus |
| ACTG_RAT | Actg1 | 42 kDa | Rattus norvegicus |
| RPN1_RAT | Rpn1 | 68 kDa | Rattus norvegicus |
| AFAD_RAT | Afdn | 208 kDa | Rattus norvegicus |
| SDHA_RAT | Sdha | 72 kDa | Rattus norvegicus |
| TTHY_RAT | Ttr | 16 kDa | Rattus norvegicus |
| LYN_RAT | Lyn | 59 kDa | Rattus norvegicus |
| FHL1_RAT | Fhl1 | 32 kDa | Rattus norvegicus |
| TPM1_RAT | Tpm1 | 33 kDa | Rattus norvegicus |
| XPO1_RAT | Xpo1 | 123 kDa | Rattus norvegicus |
| APT_RAT | Aprt | 20 kDa | Rattus norvegicus |
| ARF1_RAT | Arf1 | 21 kDa | Rattus norvegicus |
| AK1A1_RAT | Akr1a1 | 37 kDa | Rattus norvegicus |
| EF1G_RAT | Eef1g | 50 kDa | Rattus norvegicus |
| CFAI_RAT | Cfi | 67 kDa | Rattus norvegicus |
| CO9_RAT | C9 | 62 kDa | Rattus norvegicus |
| AL9A1_RAT | Aldh9a1 | 54 kDa | Rattus norvegicus |

| PFKAL_RAT | Pfkl | 85 kDa | Rattus norvegicus |
| --- | --- | --- | --- |
| ACPH_RAT | Apeh | 81 kDa | Rattus norvegicus |
| PTGIS_RAT | Ptgis | 57 kDa | Rattus norvegicus |
| VATB2_RAT | Atp6v1b2 | 57 kDa | Rattus norvegicus |
| PDIA4_RAT | Pdia4 | 73 kDa | Rattus norvegicus |
| SEP11_RAT | 11-Sep | 50 kDa | Rattus norvegicus |
| LA_RAT | Ssb | 48 kDa | Rattus norvegicus |
| ITA1_RAT | Itga1 | 131 kDa | Rattus norvegicus |
| SPTN2_RAT | Sptbn2 | 271 kDa | Rattus norvegicus |
| RET1_RAT | Rbp1 | 16 kDa | Rattus norvegicus |
| RAI14_RAT | Rai14 | 109 kDa | Rattus norvegicus |
| ADT1_RAT | Slc25a4 | 33 kDa | Rattus norvegicus |
| 6PGD_RAT | Pgd | 53 kDa | Rattus norvegicus |
| AT1A1_RAT | Atp1a1 | 113 kDa | Rattus norvegicus |
| HNRH1_RAT | Hnrnph1 | 49 kDa | Rattus norvegicus |
| MMSA_RAT | Aldh6a1 | 58 kDa | Rattus norvegicus |
| EIF3B_RAT | Eif3b | 91 kDa | Rattus norvegicus |
| HYOU1_RAT | Hyou1 | 111 kDa | Rattus norvegicus |
| CTL2_RAT | Slc44a2 | 80 kDa | Rattus norvegicus |
| ADK_RAT | Adk | 40 kDa | Rattus norvegicus |
| ACTZ_RAT | Actr1a | 43 kDa | Rattus norvegicus |
| S10AA_RAT | S100a10 | 11 kDa | Rattus norvegicus |
| FINC_RAT | Fn1 | 273 kDa | Rattus norvegicus |
| PDIA6_RAT | Pdia6 | 48 kDa | Rattus norvegicus |
| CATD_RAT | Ctsd | 45 kDa | Rattus norvegicus |
| ANXA8_RAT | Anxa8 | 37 kDa | Rattus norvegicus |
| CAZA2_RAT | Capza2 | 33 kDa | Rattus norvegicus |
| GRP75_RAT | Hspa9 | 74 kDa | Rattus norvegicus |
| K2C7_RAT | Krt7 | 51 kDa | Rattus norvegicus |
| PSA1_RAT | Psma1 | 30 kDa | Rattus norvegicus |
| HNRPF_RAT | Hnrnpf | 46 kDa | Rattus norvegicus |
| RACK1_RAT | Rack1 | 35 kDa | Rattus norvegicus |
| SEPT2_RAT | 2-Sep | 42 kDa | Rattus norvegicus |
| ES1_RAT |  | 28 kDa | Rattus norvegicus |
| K1C18_RAT | Krt18 | 48 kDa | Rattus norvegicus |
| OTUB1_RAT | Otub1 | 31 kDa | Rattus norvegicus |
| RHOA_RAT | Rhoa | 22 kDa | Rattus norvegicus |
| PPM1F_RAT | Ppm1f | 49 kDa | Rattus norvegicus |
| PSMD1_RAT | Psmd1 | 106 kDa | Rattus norvegicus |
| CAVN2_RAT | Cavin2 | 46 kDa | Rattus norvegicus |
| H2AY_RAT | H2afy | 40 kDa | Rattus norvegicus |
| EMAL2_RAT | Eml2 | 71 kDa | Rattus norvegicus |
| TCPD_RAT | Cct4 | 58 kDa | Rattus norvegicus |
| CALM1_RAT (+2) | Calm1 | 17 kDa | Rattus norvegicus |
| FUBP2_RAT | Khsrp | 74 kDa | Rattus norvegicus |
| PP2AA_RAT | Ppp2ca | 36 kDa | Rattus norvegicus |
| KAP3_RAT | Prkar2b | 46 kDa | Rattus norvegicus |

| CTNB1_RAT | Ctnnb1 | 85 kDa | Rattus norvegicus |
| --- | --- | --- | --- |
| DLDH_RAT | Dld | 54 kDa | Rattus norvegicus |
| PDC6I_RAT | Pdcd6ip | 97 kDa | Rattus norvegicus |
| PIPNA_RAT | Pitpna | 32 kDa | Rattus norvegicus |
| ENTP1_RAT | Entpd1 | 57 kDa | Rattus norvegicus |
| ALDR_RAT | Akr1b1 | 36 kDa | Rattus norvegicus |
| ARC1B_RAT | Arpc1b | 41 kDa | Rattus norvegicus |
| TIF1B_RAT | Trim28 | 89 kDa | Rattus norvegicus |
| HPT_RAT | Hp | 39 kDa | Rattus norvegicus |
| RSSA_RAT | Rpsa | 33 kDa | Rattus norvegicus |
| EST1C_RAT | Ces1c | 60 kDa | Rattus norvegicus |
| IGHG1_RAT |  | 36 kDa | Rattus norvegicus |
| ROCK2_RAT | Rock2 | 160 kDa | Rattus norvegicus |
| USO1_RAT | Uso1 | 107 kDa | Rattus norvegicus |
| FLOT1_RAT | Flot1 | 47 kDa | Rattus norvegicus |
| HNRPQ_RAT | Syncrip | 60 kDa | Rattus norvegicus |
| ACACA_RAT | Acaca | 265 kDa | Rattus norvegicus |
| SODM_RAT | Sod2 | 25 kDa | Rattus norvegicus |
| LKHA4_RAT | Lta4h | 69 kDa | Rattus norvegicus |
| LYSC1_RAT | Lyz1 | 17 kDa | Rattus norvegicus |
| OAT_RAT | Oat | 48 kDa | Rattus norvegicus |
| ADA10_RAT | Adam10 | 84 kDa | Rattus norvegicus |
| COPG1_RAT | Copg1 | 98 kDa | Rattus norvegicus |
| KINH_RAT | Kif5b | 110 kDa | Rattus norvegicus |
| APOB_RAT | Apob | 536 kDa | Rattus norvegicus |
| HNRPK_RAT | Hnrnpk | 51 kDa | Rattus norvegicus |
| DIDH_RAT | Akr1c9 | 37 kDa | Rattus norvegicus |
| VDAC1_RAT | Vdac1 | 31 kDa | Rattus norvegicus |
| IF4A2_RAT | Eif4a2 | 46 kDa | Rattus norvegicus |
| TBA1A_RAT | Tuba1a | 50 kDa | Rattus norvegicus |
| BLMH_RAT | Blmh | 52 kDa | Rattus norvegicus |
| GNAS1_RAT | Gnas | 123 kDa | Rattus norvegicus |
| MPCP_RAT | Slc25a3 | 39 kDa | Rattus norvegicus |
| SC31A_RAT | Sec31a | 135 kDa | Rattus norvegicus |
| AMPB_RAT | Rnpep | 73 kDa | Rattus norvegicus |
| BCAT2_RAT | Bcat2 | 44 kDa | Rattus norvegicus |
| PARVA_RAT | Parva | 42 kDa | Rattus norvegicus |
| FABP5_RAT | Fabp5 | 15 kDa | Rattus norvegicus |
| ECHM_RAT | Echs1 | 32 kDa | Rattus norvegicus |
| RPN2_RAT | Rpn2 | 69 kDa | Rattus norvegicus |
| CD166_RAT | Alcam | 65 kDa | Rattus norvegicus |
| PA1B2_RAT | Pafah1b2 | 26 kDa | Rattus norvegicus |
| NDKA_RAT | Nme1 | 17 kDa | Rattus norvegicus |
| DDB1_RAT | Ddb1 | 127 kDa | Rattus norvegicus |
| CAH4_RAT | Ca4 | 35 kDa | Rattus norvegicus |
| LMNB1_RAT | Lmnb1 | 67 kDa | Rattus norvegicus |
| SYTC_RAT | Tars | 81 kDa | Rattus norvegicus |

| SYWC_RAT | Wars | 54 kDa | Rattus norvegicus |
| --- | --- | --- | --- |
| VIGLN_RAT | Hdlbp | 142 kDa | Rattus norvegicus |
| PECA1_RAT | Pecam1 | 76 kDa | Rattus norvegicus |
| CRYAB_RAT | Cryab | 20 kDa | Rattus norvegicus |
| AMPE_RAT | Enpep | 108 kDa | Rattus norvegicus |
| HA12_RAT |  | 42 kDa | Rattus norvegicus |
| RL10A_RAT | Rpl10a | 25 kDa | Rattus norvegicus |
| TPIS_RAT | Tpi1 | 27 kDa | Rattus norvegicus |
| STIP1_RAT | Stip1 | 63 kDa | Rattus norvegicus |
| RL5_RAT | Rpl5 | 34 kDa | Rattus norvegicus |
| GSLG1_RAT | Glg1 | 134 kDa | Rattus norvegicus |
| DX39B_RAT | Ddx39b | 49 kDa | Rattus norvegicus |
| FIBA_RAT | Fga | 87 kDa | Rattus norvegicus |
| ODP2_RAT | Dlat | 67 kDa | Rattus norvegicus |
| AL7A1_RAT | Aldh7a1 | 59 kDa | Rattus norvegicus |
| CAV2_RAT | Cav2 | 18 kDa | Rattus norvegicus |
| DMD_RAT | Dmd | 426 kDa | Rattus norvegicus |
| PDE5A_RAT | Pde5a | 95 kDa | Rattus norvegicus |
| PYGL_RAT | Pygl | 97 kDa | Rattus norvegicus |
| ETFA_RAT | Etfa | 35 kDa | Rattus norvegicus |
| AKA12_RAT | Akap12 | 181 kDa | Rattus norvegicus |
| ACOC_RAT | Aco1 | 98 kDa | Rattus norvegicus |
| NIBL1_RAT | Fam129b | 85 kDa | Rattus norvegicus |
| PRS6A_RAT | Psmc3 | 49 kDa | Rattus norvegicus |
| DHB4_RAT | Hsd17b4 | 79 kDa | Rattus norvegicus |
| MBB1A_RAT | Mybbp1a | 152 kDa | Rattus norvegicus |
| ARPC2_RAT | Arpc2 | 34 kDa | Rattus norvegicus |
| ECE1_RAT | Ece1 | 86 kDa | Rattus norvegicus |
| CAN1_RAT | Capn1 | 82 kDa | Rattus norvegicus |
| MTOR_RAT | Mtor | 289 kDa | Rattus norvegicus |
| DOPD_RAT | Ddt | 13 kDa | Rattus norvegicus |
| G6PD_RAT | G6pdx | 59 kDa | Rattus norvegicus |
| COR1A_RAT | Coro1a | 51 kDa | Rattus norvegicus |
| ACSL5_RAT | Acsl5 | 76 kDa | Rattus norvegicus |
| AL3A2_RAT | Aldh3a2 | 54 kDa | Rattus norvegicus |
| CD9_RAT | Cd9 | 25 kDa | Rattus norvegicus |
| NDKB_RAT | Nme2 | 17 kDa | Rattus norvegicus |
| HXK1_RAT | Hk1 | 102 kDa | Rattus norvegicus |
| NEP_RAT | Mme | 86 kDa | Rattus norvegicus |
| IMDH2_RAT | Impdh2 | 56 kDa | Rattus norvegicus |
| VDAC2_RAT | Vdac2 | 32 kDa | Rattus norvegicus |
| LUM_RAT | Lum | 38 kDa | Rattus norvegicus |
| CBG_RAT | Serpina6 | 45 kDa | Rattus norvegicus |
| EIF3A_RAT | Eif3a | 163 kDa | Rattus norvegicus |
| FMO1_RAT | Fmo1 | 60 kDa | Rattus norvegicus |
| AL1A2_RAT | Aldh1a2 | 57 kDa | Rattus norvegicus |
| DPYL3_RAT | Dpysl3 | 62 kDa | Rattus norvegicus |

| GPX1_RAT | Gpx1 | 22 kDa | Rattus norvegicus |
| --- | --- | --- | --- |
| CLUS_RAT | Clu | 51 kDa | Rattus norvegicus |
| ILEUA_RAT | Serpinb1a | 43 kDa | Rattus norvegicus |
| UBP7_RAT | Usp7 | 128 kDa | Rattus norvegicus |
| CATC_RAT | Ctsc | 52 kDa | Rattus norvegicus |
| KANK2_RAT | Kank2 | 91 kDa | Rattus norvegicus |
| BIEA_RAT | Blvra | 34 kDa | Rattus norvegicus |
| MAP1B_RAT | Map1b | 270 kDa | Rattus norvegicus |
| PGRC1_RAT | Pgrmc1 | 22 kDa | Rattus norvegicus |
| ASSY_RAT | Ass1 | 46 kDa | Rattus norvegicus |
| HCD2_RAT | Hsd17b10 | 27 kDa | Rattus norvegicus |
| PRP19_RAT | Prpf19 | 55 kDa | Rattus norvegicus |
| PLCD1_RAT | Plcd1 | 86 kDa | Rattus norvegicus |
| FETUB_RAT | Fetub | 42 kDa | Rattus norvegicus |
| ODPB_RAT | Pdhb | 39 kDa | Rattus norvegicus |
| APOA4_RAT | Apoa4 | 44 kDa | Rattus norvegicus |
| SMCA4_RAT | Smarca4 | 181 kDa | Rattus norvegicus |
| KAPCA_RAT | Prkaca | 41 kDa | Rattus norvegicus |
| DESM_RAT | Des | 53 kDa | Rattus norvegicus |
| ITPR1_RAT | Itpr1 | 313 kDa | Rattus norvegicus |
| ARRB1_RAT | Arrb1 | 47 kDa | Rattus norvegicus |
| EXOC4_RAT | Exoc4 | 111 kDa | Rattus norvegicus |
| RL4_RAT | Rpl4 | 47 kDa | Rattus norvegicus |
| ADDG_RAT | Add3 | 79 kDa | Rattus norvegicus |
| ANK3_RAT | Ank3 | 284 kDa | Rattus norvegicus |
| PSD13_RAT | Psmd13 | 43 kDa | Rattus norvegicus |
| PARP1_RAT | Parp1 | 113 kDa | Rattus norvegicus |
| COMT_RAT | Comt | 30 kDa | Rattus norvegicus |
| ARHG1_RAT | Arhgef1 | 103 kDa | Rattus norvegicus |
| DDX1_RAT | Ddx1 | 82 kDa | Rattus norvegicus |
| MIC60_RAT | Immt | 67 kDa | Rattus norvegicus |
| CLIP1_RAT | Clip1 | 148 kDa | Rattus norvegicus |
| CO8B_RAT | C8b | 67 kDa | Rattus norvegicus |
| TES_RAT | Tes | 48 kDa | Rattus norvegicus |
| PLVAP_RAT | Plvap | 50 kDa | Rattus norvegicus |
| RS7_RAT | Rps7 | 22 kDa | Rattus norvegicus |
| CR1L_RAT | Cr1l | 62 kDa | Rattus norvegicus |
| AOXC_RAT | Aox3 | 147 kDa | Rattus norvegicus |
| TXNL1_RAT | Txnl1 | 32 kDa | Rattus norvegicus |
| CBPQ_RAT | Cpq | 52 kDa | Rattus norvegicus |
| SYVC_RAT | Vars | 140 kDa | Rattus norvegicus |
| MRCKB_RAT | Cdc42bpb | 195 kDa | Rattus norvegicus |
| TGFI1_RAT | Tgfb1i1 | 50 kDa | Rattus norvegicus |
| GPDM_RAT | Gpd2 | 81 kDa | Rattus norvegicus |
| NUCL_RAT | Ncl | 77 kDa | Rattus norvegicus |
| AEBP1_RAT | Aebp1 | 128 kDa | Rattus norvegicus |
| SHAN3_RAT | Shank3 | 186 kDa | Rattus norvegicus |

| RTN4_RAT | Rtn4 | 126 kDa | Rattus norvegicus |
| --- | --- | --- | --- |
| GBP2_RAT | Gbp2 | 67 kDa | Rattus norvegicus |
| RRAS_RAT | Rras | 24 kDa | Rattus norvegicus |
| HCDH_RAT | Hadh | 34 kDa | Rattus norvegicus |
| RLA0_RAT | Rplp0 | 34 kDa | Rattus norvegicus |
| VPS29_RAT | Vps29 | 20 kDa | Rattus norvegicus |
| ARP2_RAT | Actr2 | 45 kDa | Rattus norvegicus |
| PGS2_RAT | Dcn | 40 kDa | Rattus norvegicus |
| FRIH_RAT | Fth1 | 21 kDa | Rattus norvegicus |
| PTN11_RAT | Ptpn11 | 68 kDa | Rattus norvegicus |
| GCYA1_RAT | Gucy1a1 | 78 kDa | Rattus norvegicus |
| RL3_RAT | Rpl3 | 46 kDa | Rattus norvegicus |
| HNRPD_RAT | Hnrnpd | 38 kDa | Rattus norvegicus |
| IDH3B_RAT | Idh3B | 42 kDa | Rattus norvegicus |
| LEG3_RAT | Lgals3 | 27 kDa | Rattus norvegicus |
| PSME2_RAT | Psme2 | 27 kDa | Rattus norvegicus |
| ANGT_RAT | Agt | 52 kDa | Rattus norvegicus |
| RAB14_RAT | Rab14 | 24 kDa | Rattus norvegicus |
| S10A6_RAT | S100a6 | 10 kDa | Rattus norvegicus |
| PLCB4_RAT | Plcb4 | 135 kDa | Rattus norvegicus |
| TWF1_RAT | Twf1 | 40 kDa | Rattus norvegicus |
| RTCB_RAT | Rtcb | 55 kDa | Rattus norvegicus |
| ACADL_RAT | Acadl | 48 kDa | Rattus norvegicus |
| SFTPA_RAT | Sftpa1 | 26 kDa | Rattus norvegicus |
| 2AAB_RAT | Ppp2r1b | 66 kDa | Rattus norvegicus |
| PGM1_RAT | Pgm1 | 61 kDa | Rattus norvegicus |
| RS3A_RAT | Rps3a | 30 kDa | Rattus norvegicus |
| ERP29_RAT | Erp29 | 29 kDa | Rattus norvegicus |
| HYEP_RAT | Ephx1 | 53 kDa | Rattus norvegicus |
| LRP2_RAT | Lrp2 | 519 kDa | Rattus norvegicus |
| PLAK_RAT | Jup | 82 kDa | Rattus norvegicus |
| MA2A1_RAT | Man2a1 | 131 kDa | Rattus norvegicus |
| PTPRC_RAT | Ptprc | 143 kDa | Rattus norvegicus |
| CLIC1_RAT | Clic1 | 27 kDa | Rattus norvegicus |
| COX2_RAT | Mtco2 | 26 kDa | Rattus norvegicus |
| NHRF2_RAT | Slc9a3r2 | 37 kDa | Rattus norvegicus |
| PHB_RAT | Phb | 30 kDa | Rattus norvegicus |
| CDC42_RAT | Cdc42 | 21 kDa | Rattus norvegicus |
| EIF3C_RAT | Eif3c | 105 kDa | Rattus norvegicus |
| IVD_RAT | Ivd | 46 kDa | Rattus norvegicus |
| LAMB2_RAT | Lamb2 | 196 kDa | Rattus norvegicus |
| MOGS_RAT | Mogs | 92 kDa | Rattus norvegicus |
| PPCE_RAT | Prep | 81 kDa | Rattus norvegicus |
| CD36_RAT | Cd36 | 53 kDa | Rattus norvegicus |
| AATM_RAT | Got2 | 47 kDa | Rattus norvegicus |
| SYAC_RAT | Aars | 107 kDa | Rattus norvegicus |
| SYRC_RAT | Rars | 76 kDa | Rattus norvegicus |

| BIG1_RAT | Arfgef1 | 208 kDa | Rattus norvegicus |
| --- | --- | --- | --- |
| XPP1_RAT | Xpnpep1 | 70 kDa | Rattus norvegicus |
| CSK21_RAT | Csnk2a1 | 45 kDa | Rattus norvegicus |
| EMAL1_RAT | Eml1 | 90 kDa | Rattus norvegicus |
| PYGM_RAT | Pygm | 97 kDa | Rattus norvegicus |
| THIM_RAT | Acaa2 | 42 kDa | Rattus norvegicus |
| ARL8B_RAT | Arl8b | 22 kDa | Rattus norvegicus |
| CATB_RAT | Ctsb | 37 kDa | Rattus norvegicus |
| FA9_RAT | F9 | 52 kDa | Rattus norvegicus |
| PDXK_RAT | Pdxk | 35 kDa | Rattus norvegicus |
| UN13D_RAT | Unc13d | 123 kDa | Rattus norvegicus |
| NSF_RAT | Nsf | 83 kDa | Rattus norvegicus |
| SAC1_RAT | Sacm1l | 67 kDa | Rattus norvegicus |
| RL7_RAT | Rpl7 | 30 kDa | Rattus norvegicus |
| RALB_RAT | Ralb | 23 kDa | Rattus norvegicus |
| TMM43_RAT | Tmem43 | 45 kDa | Rattus norvegicus |
| DNM1L_RAT | Dnm1l | 84 kDa | Rattus norvegicus |
| TALDO_RAT | Taldo1 | 37 kDa | Rattus norvegicus |
| FARP1_RAT | Farp1 | 119 kDa | Rattus norvegicus |
| MFGM_RAT | Mfge8 | 47 kDa | Rattus norvegicus |
| TOP1_RAT | Top1 | 91 kDa | Rattus norvegicus |
| PPM1G_RAT | Ppm1g | 59 kDa | Rattus norvegicus |
| PSA5_RAT | Psma5 | 26 kDa | Rattus norvegicus |
| ADDA_RAT | Add1 | 80 kDa | Rattus norvegicus |
| PSB2_RAT | Psmb2 | 23 kDa | Rattus norvegicus |
| KAP0_RAT | Prkar1a | 43 kDa | Rattus norvegicus |
| FUMH_RAT | Fh | 54 kDa | Rattus norvegicus |
| PDS5B_RAT | Pds5b | 164 kDa | Rattus norvegicus |
| SYQ_RAT | Qars | 88 kDa | Rattus norvegicus |
| KCC2D_RAT | Camk2d | 60 kDa | Rattus norvegicus |
| LMAN1_RAT | Lman1 | 58 kDa | Rattus norvegicus |
| GLYR1_RAT | Glyr1 | 60 kDa | Rattus norvegicus |
| E41L5_RAT | Epb41l5 | 82 kDa | Rattus norvegicus |
| SEPT9_RAT | 9-Sep | 64 kDa | Rattus norvegicus |
| IDH3A_RAT | Idh3a | 40 kDa | Rattus norvegicus |
| ECHB_RAT | Hadhb | 51 kDa | Rattus norvegicus |
| AP2M1_RAT | Ap2m1 | 50 kDa | Rattus norvegicus |
| KNG1_RAT | Kng1 | 71 kDa | Rattus norvegicus |
| MCCB_RAT | Mccc2 | 62 kDa | Rattus norvegicus |
| NEDD4_RAT | Nedd4 | 102 kDa | Rattus norvegicus |
| SYDC_RAT | Dars | 57 kDa | Rattus norvegicus |
| DAB2_RAT | Dab2 | 82 kDa | Rattus norvegicus |
| S39A4_RAT | Slc39a4 | 71 kDa | Rattus norvegicus |
| AT5F1_RAT | Atp5pb | 29 kDa | Rattus norvegicus |
| LONM_RAT | Lonp1 | 106 kDa | Rattus norvegicus |
| NPM_RAT | Npm1 | 33 kDa | Rattus norvegicus |
| ARC1A_RAT | Arpc1a | 42 kDa | Rattus norvegicus |

| EFHD2_RAT | Efhd2 | 27 kDa | Rattus norvegicus |
| --- | --- | --- | --- |
| ST1A1_RAT | Sult1a1 | 34 kDa | Rattus norvegicus |
| DDAH1_RAT | Ddah1 | 31 kDa | Rattus norvegicus |
| MK01_RAT | Mapk1 | 41 kDa | Rattus norvegicus |
| PRS4_RAT | Psmc1 | 49 kDa | Rattus norvegicus |
| NIBAN_RAT | Fam129a | 103 kDa | Rattus norvegicus |
| IF4A3_RAT | Eif4a3 | 47 kDa | Rattus norvegicus |
| IF2A_RAT | Eif2s1 | 36 kDa | Rattus norvegicus |
| MDHC_RAT | Mdh1 | 36 kDa | Rattus norvegicus |
| NUCB2_RAT | Nucb2 | 50 kDa | Rattus norvegicus |
| PTN6_RAT | Ptpn6 | 70 kDa | Rattus norvegicus |
| SUCA_RAT | Suclg1 | 36 kDa | Rattus norvegicus |
| DECR_RAT | Decr1 | 36 kDa | Rattus norvegicus |
| MYPT1_RAT | Ppp1r12a | 115 kDa | Rattus norvegicus |
| GSH1_RAT | Gclc | 73 kDa | Rattus norvegicus |
| CUL5_RAT | Cul5 | 91 kDa | Rattus norvegicus |
| NLTP_RAT | Scp2 | 59 kDa | Rattus norvegicus |
| CNN1_RAT | Cnn1 | 33 kDa | Rattus norvegicus |
| ECI1_RAT | Eci1 | 32 kDa | Rattus norvegicus |
| NAMPT_RAT | Nampt | 55 kDa | Rattus norvegicus |
| NUCB1_RAT | Nucb1 | 54 kDa | Rattus norvegicus |
| ASAH1_RAT | Asah1 | 44 kDa | Rattus norvegicus |
| CAPG_RAT | Capg | 39 kDa | Rattus norvegicus |
| PABP1_RAT | Pabpc1 | 71 kDa | Rattus norvegicus |
| QCR1_RAT | Uqcrc1 | 53 kDa | Rattus norvegicus |
| IF5_RAT | Eif5 | 49 kDa | Rattus norvegicus |
| PI4KA_RAT | Pi4ka | 237 kDa | Rattus norvegicus |
| SMC1A_RAT | Smc1a | 143 kDa | Rattus norvegicus |
| TRIPC_RAT | Trip12 | 224 kDa | Rattus norvegicus |
| ICAM1_RAT | Icam1 | 60 kDa | Rattus norvegicus |
| NDRG2_RAT | Ndrg2 | 41 kDa | Rattus norvegicus |
| QCR2_RAT | Uqcrc2 | 48 kDa | Rattus norvegicus |
| STRAP_RAT | Strap | 38 kDa | Rattus norvegicus |
| RS15A_RAT | Rps15a | 15 kDa | Rattus norvegicus |
| PPM1A_RAT | Ppm1a | 42 kDa | Rattus norvegicus |
| OPLA_RAT | Oplah | 138 kDa | Rattus norvegicus |
| UB2V2_RAT | Ube2v2 | 16 kDa | Rattus norvegicus |
| CRYL1_RAT | Cryl1 | 35 kDa | Rattus norvegicus |
| KCY_RAT | Cmpk1 | 22 kDa | Rattus norvegicus |
| PSB8_RAT | Psmb8 | 31 kDa | Rattus norvegicus |
| GLOD4_RAT | Glod4 | 33 kDa | Rattus norvegicus |
| NTF2_RAT | Nutf2 | 14 kDa | Rattus norvegicus |
| EIF3H_RAT | Eif3h | 40 kDa | Rattus norvegicus |
| NP1L4_RAT | Nap1l4 | 44 kDa | Rattus norvegicus |
| OST48_RAT | Ddost | 49 kDa | Rattus norvegicus |
| PLCB1_RAT | Plcb1 | 138 kDa | Rattus norvegicus |
| PRDX5_RAT | Prdx5 | 22 kDa | Rattus norvegicus |

| PSB3_RAT | Psmb3 | 23 kDa | Rattus norvegicus |
| --- | --- | --- | --- |
| BST1_RAT | Bst1 | 35 kDa | Rattus norvegicus |
| HS105_RAT | Hsph1 | 96 kDa | Rattus norvegicus |
| LXN_RAT | Lxn | 26 kDa | Rattus norvegicus |
| MYO1E_RAT | Myo1e | 127 kDa | Rattus norvegicus |
| TPP2_RAT | Tpp2 | 138 kDa | Rattus norvegicus |
| GIMA4_RAT | Gimap4 | 36 kDa | Rattus norvegicus |
| CSN4_RAT | Cops4 | 46 kDa | Rattus norvegicus |
| LANC1_RAT | Lancl1 | 45 kDa | Rattus norvegicus |
| PODXL_RAT | Podxl | 52 kDa | Rattus norvegicus |
| PSA6_RAT | Psma6 | 27 kDa | Rattus norvegicus |
| COR1B_RAT | Coro1b | 54 kDa | Rattus norvegicus |
| HDGF_RAT | Hdgf | 26 kDa | Rattus norvegicus |
| MCCA_RAT | Mccc1 | 79 kDa | Rattus norvegicus |
| GPX2_RAT | Gpx2 | 22 kDa | Rattus norvegicus |
| PYC_RAT | Pc | 130 kDa | Rattus norvegicus |
| C1QBP_RAT | C1qbp | 31 kDa | Rattus norvegicus |
| ERAP1_RAT | Erap1 | 106 kDa | Rattus norvegicus |
| GLSK_RAT | Gls | 74 kDa | Rattus norvegicus |
| TPR_RAT | Tpr | 267 kDa | Rattus norvegicus |
| APOE_RAT | Apoe | 36 kDa | Rattus norvegicus |
| STXB1_RAT | Stxbp1 | 68 kDa | Rattus norvegicus |
| TM9S2_RAT | Tm9sf2 | 76 kDa | Rattus norvegicus |
| CATH_RAT | Ctsh | 37 kDa | Rattus norvegicus |
| FAK1_RAT | Ptk2 | 120 kDa | Rattus norvegicus |
| KAP2_RAT | Prkar2a | 45 kDa | Rattus norvegicus |
| FKBP4_RAT | Fkbp4 | 51 kDa | Rattus norvegicus |
| LDHB_RAT | Ldhb | 37 kDa | Rattus norvegicus |
| ITPA_RAT | Itpa | 22 kDa | Rattus norvegicus |
| HINT1_RAT | Hint1 | 14 kDa | Rattus norvegicus |
| SNAA_RAT | Napa | 33 kDa | Rattus norvegicus |
| STXB2_RAT | Stxbp2 | 67 kDa | Rattus norvegicus |
| GPX3_RAT | Gpx3 | 25 kDa | Rattus norvegicus |
| ATP5H_RAT | Atp5pd | 19 kDa | Rattus norvegicus |
| IF2P_RAT | Eif5b | 138 kDa | Rattus norvegicus |
| PCCA_RAT | Pcca | 82 kDa | Rattus norvegicus |
| PCYOX_RAT | Pcyox1 | 56 kDa | Rattus norvegicus |
| RS3_RAT | Rps3 | 27 kDa | Rattus norvegicus |
| CALU_RAT | Calu | 37 kDa | Rattus norvegicus |
| 6PGL_RAT | Pgls | 27 kDa | Rattus norvegicus |
| PA2G4_RAT | Pa2g4 | 44 kDa | Rattus norvegicus |
| ALS_RAT | Igfals | 67 kDa | Rattus norvegicus |
| GARS_RAT | Gars | 72 kDa | Rattus norvegicus |
| PSA4_RAT | Psma4 | 29 kDa | Rattus norvegicus |
| RS24_RAT | Rps24 | 15 kDa | Rattus norvegicus |
| ODPA_RAT | Pdha1 | 43 kDa | Rattus norvegicus |
| RAB7A_RAT | Rab7a | 24 kDa | Rattus norvegicus |

| ANM1_RAT | Prmt1 | 41 kDa | Rattus norvegicus |
| --- | --- | --- | --- |
| SPA3N_RAT | Serpina3n | 47 kDa | Rattus norvegicus |
| THTR_RAT | Tst | 33 kDa | Rattus norvegicus |
| GOGA4_RAT | Golga4 | 260 kDa | Rattus norvegicus |
| 2ABA_RAT | Ppp2r2a | 52 kDa | Rattus norvegicus |
| CADM1_RAT | Cadm1 | 52 kDa | Rattus norvegicus |
| NCEH1_RAT | Nceh1 | 46 kDa | Rattus norvegicus |
| SYK_RAT | Kars | 72 kDa | Rattus norvegicus |
| MATR3_RAT | Matr3 | 94 kDa | Rattus norvegicus |
| ACSF2_RAT | Acsf2 | 68 kDa | Rattus norvegicus |
| ACADV_RAT | Acadvl | 71 kDa | Rattus norvegicus |
| AATC_RAT | Got1 | 46 kDa | Rattus norvegicus |
| PRS8_RAT | Psmc5 | 46 kDa | Rattus norvegicus |
| VWA5A_RAT | Vwa5a | 91 kDa | Rattus norvegicus |
| DC1L1_RAT | Dync1li1 | 57 kDa | Rattus norvegicus |
| PLCB3_RAT | Plcb3 | 139 kDa | Rattus norvegicus |
| AP1M1_RAT | Ap1m1 | 49 kDa | Rattus norvegicus |
| PSPB_RAT | Sftpb | 42 kDa | Rattus norvegicus |
| MA2C1_RAT | Man2c1 | 116 kDa | Rattus norvegicus |
| RASH_RAT | Hras | 21 kDa | Rattus norvegicus |
| SCFD1_RAT | Scfd1 | 72 kDa | Rattus norvegicus |
| STA5B_RAT | Stat5b | 90 kDa | Rattus norvegicus |
| RTN3_RAT | Rtn3 | 102 kDa | Rattus norvegicus |
| ARF6_RAT | Arf6 | 20 kDa | Rattus norvegicus |
| IF2G_RAT | Eif2s3 | 51 kDa | Rattus norvegicus |
| FPPS_RAT | Fdps | 41 kDa | Rattus norvegicus |
| CAPR1_RAT | Caprin1 | 78 kDa | Rattus norvegicus |
| MK03_RAT | Mapk3 | 43 kDa | Rattus norvegicus |
| MRC2_RAT | Mrc2 | 167 kDa | Rattus norvegicus |
| RHG17_RAT | Arhgap17 | 94 kDa | Rattus norvegicus |
| VP26A_RAT | Vps26a | 38 kDa | Rattus norvegicus |
| CK054_RAT |  | 35 kDa | Rattus norvegicus |
| VAPA_RAT | Vapa | 28 kDa | Rattus norvegicus |
| RL9_RAT | Rpl9 | 22 kDa | Rattus norvegicus |
| AL1A3_RAT | Aldh1a3 | 56 kDa | Rattus norvegicus |
| MAGI3_RAT | Magi3 | 161 kDa | Rattus norvegicus |
| PAXI_RAT | Pxn | 64 kDa | Rattus norvegicus |
| MTPN_RAT | Mtpn | 13 kDa | Rattus norvegicus |
| SLMAP_RAT | Slmap | 98 kDa | Rattus norvegicus |
| VDAC3_RAT | Vdac3 | 31 kDa | Rattus norvegicus |
| CALD1_RAT | Cald1 | 61 kDa | Rattus norvegicus |
| NMT1_RAT | Nmt1 | 57 kDa | Rattus norvegicus |
| ADH1_RAT | Adh1 | 40 kDa | Rattus norvegicus |
| ATLA3_RAT | Atl3 | 61 kDa | Rattus norvegicus |
| SPRC_RAT | Sparc | 34 kDa | Rattus norvegicus |
| PLCG1_RAT | Plcg1 | 149 kDa | Rattus norvegicus |
| CNN3_RAT | Cnn3 | 36 kDa | Rattus norvegicus |

| CSK_RAT | Csk | 51 kDa | Rattus norvegicus |
| --- | --- | --- | --- |
| SMC3_RAT | Smc3 | 138 kDa | Rattus norvegicus |
| RLA2_RAT | Rplp2 | 12 kDa | Rattus norvegicus |
| CAN5_RAT | Capn5 | 73 kDa | Rattus norvegicus |
| PP1R7_RAT | Ppp1r7 | 41 kDa | Rattus norvegicus |
| H15_RAT | Hist1h1b | 23 kDa | Rattus norvegicus |
| APEX1_RAT | Apex1 | 36 kDa | Rattus norvegicus |
| PON2_RAT | Pon2 | 40 kDa | Rattus norvegicus |
| RUVB1_RAT | Ruvbl1 | 50 kDa | Rattus norvegicus |
| DCTN2_RAT | Dctn2 | 44 kDa | Rattus norvegicus |
| KAD2_RAT | Ak2 | 26 kDa | Rattus norvegicus |
| NHRF1_RAT | Slc9a3r1 | 39 kDa | Rattus norvegicus |
| PCAT1_RAT | Lpcat1 | 60 kDa | Rattus norvegicus |
| GSTA4_RAT | Gsta4 | 26 kDa | Rattus norvegicus |
| HD_RAT | Htt | 344 kDa | Rattus norvegicus |
| HNRH2_RAT | Hnrnph2 | 49 kDa | Rattus norvegicus |
| CYB5_RAT | Cyb5a | 15 kDa | Rattus norvegicus |
| EIF3D_RAT | Eif3d | 64 kDa | Rattus norvegicus |
| OLA1_RAT | Ola1 | 45 kDa | Rattus norvegicus |
| PPIB_RAT | Ppib | 24 kDa | Rattus norvegicus |
| PSA2_RAT | Psma2 | 26 kDa | Rattus norvegicus |
| GSTT2_RAT | Gstt2 | 27 kDa | Rattus norvegicus |
| ROCK1_RAT | Rock1 | 160 kDa | Rattus norvegicus |
| SCOT1_RAT | Oxct1 | 56 kDa | Rattus norvegicus |
| GCP60_RAT | Acbd3 | 60 kDa | Rattus norvegicus |
| MGLL_RAT | Mgll | 33 kDa | Rattus norvegicus |
| ABCD3_RAT | Abcd3 | 75 kDa | Rattus norvegicus |
| ADA_RAT | Ada | 40 kDa | Rattus norvegicus |
| ATPO_RAT | Atp5po | 23 kDa | Rattus norvegicus |
| MECP2_RAT | Mecp2 | 53 kDa | Rattus norvegicus |
| FMO3_RAT | Fmo3 | 60 kDa | Rattus norvegicus |
| H11_RAT | Hist1h1a | 22 kDa | Rattus norvegicus |
| NIT1_RAT | Nit1 | 36 kDa | Rattus norvegicus |
| CANB1_RAT | Ppp3r1 | 19 kDa | Rattus norvegicus |
| RL8_RAT | Rpl8 | 28 kDa | Rattus norvegicus |
| DYL2_RAT | Dynll2 | 10 kDa | Rattus norvegicus |
| CIP4_RAT | Trip10 | 63 kDa | Rattus norvegicus |
| PRS6B_RAT | Psmc4 | 47 kDa | Rattus norvegicus |
| ESTD_RAT | Esd | 31 kDa | Rattus norvegicus |
| PAK2_RAT | Pak2 | 58 kDa | Rattus norvegicus |
| ARHG2_RAT | Arhgef2 | 112 kDa | Rattus norvegicus |
| DC1L2_RAT | Dync1li2 | 55 kDa | Rattus norvegicus |
| MUG2_RAT | Mug2 | 162 kDa | Rattus norvegicus |
| FBRL_RAT | Fbl | 34 kDa | Rattus norvegicus |
| NEXN_RAT | Nexn | 78 kDa | Rattus norvegicus |
| RL23_RAT | Rpl23 | 15 kDa | Rattus norvegicus |
| SCRB2_RAT | Scarb2 | 54 kDa | Rattus norvegicus |

| AGO2_RAT | Ago2 | 97 kDa | Rattus norvegicus |
| --- | --- | --- | --- |
| EFTU_RAT | Tufm | 50 kDa | Rattus norvegicus |
| PCCB_RAT | Pccb | 59 kDa | Rattus norvegicus |
| RAB8A_RAT | Rab8a | 24 kDa | Rattus norvegicus |
| KCC2G_RAT | Camk2g | 59 kDa | Rattus norvegicus |
| KLKB1_RAT | Klkb1 | 71 kDa | Rattus norvegicus |
| RBBP9_RAT | Rbbp9 | 21 kDa | Rattus norvegicus |
| SLK_RAT | Slk | 138 kDa | Rattus norvegicus |
| MYO5A_RAT | Myo5a | 212 kDa | Rattus norvegicus |
| GSK3B_RAT | Gsk3b | 47 kDa | Rattus norvegicus |
| SEPT8_RAT | 8-Sep | 51 kDa | Rattus norvegicus |
| YES_RAT | Yes1 | 61 kDa | Rattus norvegicus |
| RB11A_RAT | Rab11a | 24 kDa | Rattus norvegicus |
| THOP1_RAT | Thop1 | 78 kDa | Rattus norvegicus |
| OSTF1_RAT | Ostf1 | 24 kDa | Rattus norvegicus |
| PPID_RAT | Ppid | 41 kDa | Rattus norvegicus |
| TBB2A_RAT | Tubb2a | 50 kDa | Rattus norvegicus |
| HDAC1_RAT | Hdac1 | 55 kDa | Rattus norvegicus |
| IDHG1_RAT | Idh3g | 43 kDa | Rattus norvegicus |
| KAD1_RAT | Ak1 | 22 kDa | Rattus norvegicus |
| CATZ_RAT | Ctsz | 34 kDa | Rattus norvegicus |
| COPD_RAT | Arcn1 | 57 kDa | Rattus norvegicus |
| CYTSA_RAT | Specc1l | 124 kDa | Rattus norvegicus |
| GNA13_RAT | Gna13 | 44 kDa | Rattus norvegicus |
| PGH1_RAT | Ptgs1 | 69 kDa | Rattus norvegicus |
| TEBP_RAT | Ptges3 | 19 kDa | Rattus norvegicus |
| CX6A1_RAT | Cox6a1 | 12 kDa | Rattus norvegicus |
| ATX10_RAT | Atxn10 | 54 kDa | Rattus norvegicus |
| FUBP1_RAT | Fubp1 | 67 kDa | Rattus norvegicus |
| PRDX3_RAT | Prdx3 | 28 kDa | Rattus norvegicus |
| AP1B1_RAT | Ap1b1 | 105 kDa | Rattus norvegicus |
| HNRPC_RAT | Hnrnpc | 33 kDa | Rattus norvegicus |
| PIMT_RAT | Pcmt1 | 25 kDa | Rattus norvegicus |
| GUAA_RAT | Gmps | 77 kDa | Rattus norvegicus |
| PTGR1_RAT | Ptgr1 | 36 kDa | Rattus norvegicus |
| TPPP3_RAT | Tppp3 | 19 kDa | Rattus norvegicus |
| EXOC2_RAT | Exoc2 | 104 kDa | Rattus norvegicus |
| HERC4_RAT | Herc4 | 119 kDa | Rattus norvegicus |
| KLC1_RAT | Klc1 | 64 kDa | Rattus norvegicus |
| LAC2_RAT |  | 11 kDa | Rattus norvegicus |
| LPPRC_RAT | Lrpprc | 157 kDa | Rattus norvegicus |
| MP2K1_RAT | Map2k1 | 43 kDa | Rattus norvegicus |
| NAGAB_RAT | Naga | 47 kDa | Rattus norvegicus |
| NCBP1_RAT | Ncbp1 | 92 kDa | Rattus norvegicus |
| PPP5_RAT | Ppp5c | 57 kDa | Rattus norvegicus |
| PPM1B_RAT | Ppm1b | 43 kDa | Rattus norvegicus |
| AL4A1_RAT | Aldh4a1 | 62 kDa | Rattus norvegicus |

| HPRT_RAT | Hprt1 | 24 kDa | Rattus norvegicus |
| --- | --- | --- | --- |
| ESAM_RAT | Esam | 42 kDa | Rattus norvegicus |
| LAP2_RAT | Tmpo | 50 kDa | Rattus norvegicus |
| CO6_RAT | C6 | 105 kDa | Rattus norvegicus |
| HEP2_RAT | Serpind1 | 55 kDa | Rattus norvegicus |
| STK24_RAT | Stk24 | 48 kDa | Rattus norvegicus |
| ERMP1_RAT | Ermp1 | 100 kDa | Rattus norvegicus |
| AKAP5_RAT | Akap5 | 76 kDa | Rattus norvegicus |
| CRIP2_RAT | Crip2 | 23 kDa | Rattus norvegicus |
| 4F2_RAT | Slc3a2 | 58 kDa | Rattus norvegicus |
| CA2D1_RAT | Cacna2d1 | 124 kDa | Rattus norvegicus |
| GSHR_RAT | Gsr | 46 kDa | Rattus norvegicus |
| PLGT3_RAT | Poglut3 | 59 kDa | Rattus norvegicus |
| NP1L1_RAT | Nap1l1 | 45 kDa | Rattus norvegicus |
| PON1_RAT | Pon1 | 39 kDa | Rattus norvegicus |
| 1433B_RAT | Ywhab | 28 kDa | Rattus norvegicus |
| PSD11_RAT | Psmd11 | 47 kDa | Rattus norvegicus |
| PARD3_RAT | Pard3 | 149 kDa | Rattus norvegicus |
| DDAH2_RAT | Ddah2 | 30 kDa | Rattus norvegicus |
| DDX21_RAT | Ddx21 | 86 kDa | Rattus norvegicus |
| DHI1_RAT | Hsd11b1 | 32 kDa | Rattus norvegicus |
| BAG6_RAT | Bag6 | 120 kDa | Rattus norvegicus |
| EIF3I_RAT | Eif3i | 36 kDa | Rattus norvegicus |
| SNX5_RAT | Snx5 | 47 kDa | Rattus norvegicus |
| RL17_RAT | Rpl17 | 21 kDa | Rattus norvegicus |
| CUL3_RAT | Cul3 | 89 kDa | Rattus norvegicus |
| IC1_RAT | Serping1 | 56 kDa | Rattus norvegicus |
| THTM_RAT | Mpst | 33 kDa | Rattus norvegicus |
| ABCF1_RAT | Abcf1 | 95 kDa | Rattus norvegicus |
| EHD3_RAT | Ehd3 | 61 kDa | Rattus norvegicus |
| LG3BP_RAT | Lgals3bp | 64 kDa | Rattus norvegicus |
| NEUL_RAT | Nln | 80 kDa | Rattus norvegicus |
| PEPD_RAT | Pepd | 55 kDa | Rattus norvegicus |
| SPB10_RAT | Serpinb10 | 45 kDa | Rattus norvegicus |
| CCD22_RAT | Ccdc22 | 71 kDa | Rattus norvegicus |
| GSTM5_RAT | Gstm5 | 27 kDa | Rattus norvegicus |
| PRDX4_RAT | Prdx4 | 31 kDa | Rattus norvegicus |
| SYSC_RAT | Sars | 59 kDa | Rattus norvegicus |
| DCPS_RAT | Dcps | 39 kDa | Rattus norvegicus |
| GIMA8_RAT | Gimap8 | 77 kDa | Rattus norvegicus |
| RL7A_RAT | Rpl7a | 30 kDa | Rattus norvegicus |
| WDR61_RAT | Wdr61 | 34 kDa | Rattus norvegicus |
| PURA_RAT | Pura | 15 kDa | Rattus norvegicus |
| RAB21_RAT | Rab21 | 24 kDa | Rattus norvegicus |
| HDGR2_RAT | Hdgfl2 | 74 kDa | Rattus norvegicus |
| RS15_RAT | Rps15 | 17 kDa | Rattus norvegicus |
| A16A1_RAT | Aldh16a1 | 85 kDa | Rattus norvegicus |

| EIF3E_RAT | Eif3e | 52 kDa | Rattus norvegicus |
| --- | --- | --- | --- |
| EPCAM_RAT | Epcam | 35 kDa | Rattus norvegicus |
| HPCL1_RAT | Hpcal1 | 22 kDa | Rattus norvegicus |
| RBBP7_RAT | Rbbp7 | 48 kDa | Rattus norvegicus |
| VPS25_RAT | Vps25 | 21 kDa | Rattus norvegicus |
| PPT1_RAT | Ppt1 | 34 kDa | Rattus norvegicus |
| SEC13_RAT | Sec13 | 36 kDa | Rattus norvegicus |
| LEG5_RAT | Lgals5 | 16 kDa | Rattus norvegicus |
| AT2B4_RAT | Atp2b4 | 133 kDa | Rattus norvegicus |
| PDP1_RAT | Pdp1 | 61 kDa | Rattus norvegicus |
| C1QR1_RAT | Cd93 | 69 kDa | Rattus norvegicus |
| CTL1_RAT | Slc44a1 | 73 kDa | Rattus norvegicus |
| 1433G_RAT | Ywhag | 28 kDa | Rattus norvegicus |
| FA12_RAT | F12 | 66 kDa | Rattus norvegicus |
| RAB1B_RAT | Rab1b | 22 kDa | Rattus norvegicus |
| RS2_RAT | Rps2 | 31 kDa | Rattus norvegicus |
| EF1D_RAT | Eef1d | 31 kDa | Rattus norvegicus |
| GNAQ_RAT | Gnaq | 42 kDa | Rattus norvegicus |
| PSIP1_RAT | Psip1 | 60 kDa | Rattus norvegicus |
| AKP13_RAT | Akap13 | 301 kDa | Rattus norvegicus |
| ENPP1_RAT | Enpp1 | 103 kDa | Rattus norvegicus |
| RET4_RAT | Rbp4 | 23 kDa | Rattus norvegicus |
| FLOT2_RAT | Flot2 | 47 kDa | Rattus norvegicus |
| KPCI_RAT | Prkci | 68 kDa | Rattus norvegicus |
| PSA7_RAT | Psma7 | 28 kDa | Rattus norvegicus |
| CYC_RAT | Cycs | 12 kDa | Rattus norvegicus |
| MPI_RAT | Mpi | 46 kDa | Rattus norvegicus |
| HP1B3_RAT | Hp1bp3 | 61 kDa | Rattus norvegicus |
| ACY1A_RAT | Acy1a | 46 kDa | Rattus norvegicus |
| PSB10_RAT | Psmb10 | 29 kDa | Rattus norvegicus |
| HIBCH_RAT | Hibch | 43 kDa | Rattus norvegicus |
| APOH_RAT | Apoh | 33 kDa | Rattus norvegicus |
| PICAL_RAT | Picalm | 69 kDa | Rattus norvegicus |
| SRP54_RAT | Srp54 | 56 kDa | Rattus norvegicus |
| UFL1_RAT | Ufl1 | 90 kDa | Rattus norvegicus |
| A4_RAT | App | 87 kDa | Rattus norvegicus |
| AOFA_RAT | Maoa | 60 kDa | Rattus norvegicus |
| CRKL_RAT | Crkl | 34 kDa | Rattus norvegicus |
| MYO5B_RAT | Myo5b | 214 kDa | Rattus norvegicus |
| NDUS1_RAT | Ndufs1 | 79 kDa | Rattus norvegicus |
| SHLB1_RAT | Sh3glb1 | 41 kDa | Rattus norvegicus |
| AOFB_RAT | Maob | 58 kDa | Rattus norvegicus |
| LETM1_RAT | Letm1 | 83 kDa | Rattus norvegicus |
| PAFA2_RAT | Pafah2 | 43 kDa | Rattus norvegicus |
| PRS7_RAT | Psmc2 | 49 kDa | Rattus norvegicus |
| RAB2A_RAT | Rab2a | 24 kDa | Rattus norvegicus |
| PDS5A_RAT | Pds5a | 150 kDa | Rattus norvegicus |

| CPNE1_RAT | Cpne1 | 59 kDa | Rattus norvegicus |
| --- | --- | --- | --- |
| ERLN2_RAT | Erlin2 | 38 kDa | Rattus norvegicus |
| CP2F2_RAT | Cyp2f2 | 56 kDa | Rattus norvegicus |
| SKP1_RAT | Skp1 | 19 kDa | Rattus norvegicus |
| ASAP1_RAT | Asap1 | 127 kDa | Rattus norvegicus |
| CADH1_RAT | Cdh1 | 99 kDa | Rattus norvegicus |
| CISY_RAT | Cs | 52 kDa | Rattus norvegicus |
| K2C5_RAT | Krt5 | 62 kDa | Rattus norvegicus |
| CYBP_RAT | Cacybp | 27 kDa | Rattus norvegicus |
| SAE1_RAT | Sae1 | 39 kDa | Rattus norvegicus |
| SIR2_RAT | Sirt2 | 39 kDa | Rattus norvegicus |
| CSN2_RAT | Cops2 | 52 kDa | Rattus norvegicus |
| NIT2_RAT | Nit2 | 31 kDa | Rattus norvegicus |
| RENBP_RAT | Renbp | 50 kDa | Rattus norvegicus |
| GIPC2_RAT | Gipc2 | 34 kDa | Rattus norvegicus |
| RAN_RAT | Ran | 24 kDa | Rattus norvegicus |
| NPL4_RAT | Nploc4 | 68 kDa | Rattus norvegicus |
| BIN2_RAT | Bin2 | 55 kDa | Rattus norvegicus |
| ELAV1_RAT | Elavl1 | 36 kDa | Rattus norvegicus |
| GSTM1_RAT | Gstm1 | 26 kDa | Rattus norvegicus |
| GSTO1_RAT | Gsto1 | 28 kDa | Rattus norvegicus |
| LYPA2_RAT | Lypla2 | 25 kDa | Rattus norvegicus |
| TCTP_RAT | Tpt1 | 19 kDa | Rattus norvegicus |
| PAIRB_RAT | Serbp1 | 45 kDa | Rattus norvegicus |
| ITPR2_RAT | Itpr2 | 307 kDa | Rattus norvegicus |
| BAG5_RAT | Bag5 | 51 kDa | Rattus norvegicus |
| CN37_RAT | Cnp | 47 kDa | Rattus norvegicus |
| ERO1A_RAT | Ero1a | 54 kDa | Rattus norvegicus |
| ARK72_RAT | Akr7a2 | 41 kDa | Rattus norvegicus |
| TKFC_RAT | Tkfc | 59 kDa | Rattus norvegicus |
| UBE4A_RAT | Ube4a | 122 kDa | Rattus norvegicus |
| CSN1_RAT | Gps1 | 53 kDa | Rattus norvegicus |
| RL12_RAT | Rpl12 | 18 kDa | Rattus norvegicus |
| RL6_RAT | Rpl6 | 34 kDa | Rattus norvegicus |
| ACSL1_RAT | Acsl1 | 78 kDa | Rattus norvegicus |
| ADHX_RAT | Adh5 | 40 kDa | Rattus norvegicus |
| ARHG7_RAT | Arhgef7 | 73 kDa | Rattus norvegicus |
| PIPNB_RAT | Pitpnb | 31 kDa | Rattus norvegicus |
| RBGPR_RAT | Rab3gap2 | 154 kDa | Rattus norvegicus |
| LYPA1_RAT | Lypla1 | 25 kDa | Rattus norvegicus |
| NNRD_RAT | Naxd | 37 kDa | Rattus norvegicus |
| PLEK_RAT | Plek | 40 kDa | Rattus norvegicus |
| S29A1_RAT | Slc29a1 | 50 kDa | Rattus norvegicus |
| AP4A_RAT | Nudt2 | 17 kDa | Rattus norvegicus |
| SDHB_RAT | Sdhb | 32 kDa | Rattus norvegicus |
| SYYC_RAT | Yars | 59 kDa | Rattus norvegicus |
| TNR6_RAT | Fas | 37 kDa | Rattus norvegicus |

| CSN3_RAT | Cops3 | 48 kDa | Rattus norvegicus |
| --- | --- | --- | --- |
| DBNL_RAT | Dbnl | 49 kDa | Rattus norvegicus |
| MGN_RAT | Magoh | 17 kDa | Rattus norvegicus |
| PPBT_RAT | Alpl | 58 kDa | Rattus norvegicus |
| PSB1_RAT | Psmb1 | 26 kDa | Rattus norvegicus |
| SNX1_RAT | Snx1 | 59 kDa | Rattus norvegicus |
| 1433F_RAT | Ywhah | 28 kDa | Rattus norvegicus |
| VATE1_RAT | Atp6v1e1 | 26 kDa | Rattus norvegicus |
| FMOD_RAT | Fmod | 43 kDa | Rattus norvegicus |
| MAP4_RAT | Map4 | 110 kDa | Rattus norvegicus |
| TAP1_RAT | Tap1 | 79 kDa | Rattus norvegicus |
| NU155_RAT | Nup155 | 155 kDa | Rattus norvegicus |
| ABD12_RAT | Abhd12 | 45 kDa | Rattus norvegicus |
| CCD47_RAT | Ccdc47 | 56 kDa | Rattus norvegicus |
| EDC4_RAT | Edc4 | 153 kDa | Rattus norvegicus |
| OPA1_RAT | Opa1 | 111 kDa | Rattus norvegicus |
| PHB2_RAT | Phb2 | 33 kDa | Rattus norvegicus |
| C1TC_RAT | Mthfd1 | 101 kDa | Rattus norvegicus |
| FAHD2_RAT | Fahd2 | 35 kDa | Rattus norvegicus |
| UCHL3_RAT | Uchl3 | 26 kDa | Rattus norvegicus |
| RMD2_RAT | Rmdn2 | 47 kDa | Rattus norvegicus |
| CHD5_RAT | Chd5 | 222 kDa | Rattus norvegicus |
| YBOX1_RAT | Ybx1 | 36 kDa | Rattus norvegicus |
| ABI1_RAT | Abi1 | 52 kDa | Rattus norvegicus |
| ARL1_RAT | Arl1 | 20 kDa | Rattus norvegicus |
| BIN1_RAT | Bin1 | 65 kDa | Rattus norvegicus |
| CPT2_RAT | Cpt2 | 74 kDa | Rattus norvegicus |
| DNJA1_RAT | Dnaja1 | 45 kDa | Rattus norvegicus |
| GBB4_RAT | Gnb4 | 37 kDa | Rattus norvegicus |
| MARE1_RAT | Mapre1 | 30 kDa | Rattus norvegicus |
| FBLN3_RAT | Efemp1 | 55 kDa | Rattus norvegicus |
| ETFB_RAT | Etfb | 28 kDa | Rattus norvegicus |
| MAP2_RAT | Metap2 | 53 kDa | Rattus norvegicus |
| NUP98_RAT | Nup98 | 197 kDa | Rattus norvegicus |
| NID1_RAT | Nid1 | 36 kDa | Rattus norvegicus |
| PI3R4_RAT | Pik3r4 | 152 kDa | Rattus norvegicus |
| UBP15_RAT | Usp15 | 109 kDa | Rattus norvegicus |
| TR150_RAT | Thrap3 | 108 kDa | Rattus norvegicus |
| CLAP2_RAT | Clasp2 | 141 kDa | Rattus norvegicus |
| AAPK1_RAT | Prkaa1 | 64 kDa | Rattus norvegicus |
| IAH1_RAT | Iah1 | 28 kDa | Rattus norvegicus |
| ADPRH_RAT | Adprh | 40 kDa | Rattus norvegicus |
| COTL1_RAT | Cotl1 | 16 kDa | Rattus norvegicus |
| RAB35_RAT | Rab35 | 23 kDa | Rattus norvegicus |
| GPDA_RAT | Gpd1 | 37 kDa | Rattus norvegicus |
| HMGN5_RAT | Hmgn5 | 49 kDa | Rattus norvegicus |
| CP1A1_RAT | Cyp1a1 | 59 kDa | Rattus norvegicus |

| PRP6_RAT | Prpf6 | 107 kDa | Rattus norvegicus |
| --- | --- | --- | --- |
| THIKA_RAT | Acaa1a | 44 kDa | Rattus norvegicus |
| DDX46_RAT | Ddx46 | 117 kDa | Rattus norvegicus |
| UN45A_RAT | Unc45a | 103 kDa | Rattus norvegicus |
| DKC1_RAT | Dkc1 | 57 kDa | Rattus norvegicus |
| ADAS_RAT | Agps | 72 kDa | Rattus norvegicus |
| CBR1_RAT | Cbr1 | 31 kDa | Rattus norvegicus |
| PTBP3_RAT | Ptbp3 | 57 kDa | Rattus norvegicus |
| C4BPA_RAT | C4bpa | 62 kDa | Rattus norvegicus |
| TMEDA_RAT | Tmed10 | 25 kDa | Rattus norvegicus |
| RS11_RAT | Rps11 | 18 kDa | Rattus norvegicus |
| ACOX1_RAT | Acox1 | 75 kDa | Rattus norvegicus |
| ARL3_RAT | Arl3 | 20 kDa | Rattus norvegicus |
| NRP1_RAT | Nrp1 | 103 kDa | Rattus norvegicus |
| PALLD_RAT | Palld | 67 kDa | Rattus norvegicus |
| SYFA_RAT | Farsa | 58 kDa | Rattus norvegicus |
| CATS_RAT | Ctss | 37 kDa | Rattus norvegicus |
| DLG1_RAT | Dlg1 | 101 kDa | Rattus norvegicus |
| SGT1_RAT | Sugt1 | 38 kDa | Rattus norvegicus |
| CDC37_RAT | Cdc37 | 45 kDa | Rattus norvegicus |
| INO1_RAT | Isyna1 | 61 kDa | Rattus norvegicus |
| METK2_RAT | Mat2a | 44 kDa | Rattus norvegicus |
| MYZAP_RAT | Myzap | 54 kDa | Rattus norvegicus |
| SPON1_RAT | Spon1 | 91 kDa | Rattus norvegicus |
| CRK_RAT | Crk | 34 kDa | Rattus norvegicus |
| ACSL4_RAT | Acsl4 | 74 kDa | Rattus norvegicus |
| HNRDL_RAT | Hnrnpdl | 35 kDa | Rattus norvegicus |
| SORT_RAT | Sort1 | 91 kDa | Rattus norvegicus |
| PTN23_RAT | Ptpn23 | 163 kDa | Rattus norvegicus |
| AGRF5_RAT | Adgrf5 | 149 kDa | Rattus norvegicus |
| OSBL1_RAT | Osbpl1a | 108 kDa | Rattus norvegicus |
| ECI2_RAT | Eci2 | 43 kDa | Rattus norvegicus |
| K1C10_RAT | Krt10 | 57 kDa | Rattus norvegicus |
| LMF2_RAT | Lmf2 | 80 kDa | Rattus norvegicus |
| AT2A3_RAT | Atp2a3 | 116 kDa | Rattus norvegicus |
| IFM3_RAT | ifitm3 | 15 kDa | Rattus norvegicus |
| AT1B3_RAT | Atp1b3 | 32 kDa | Rattus norvegicus |
| CDS2_RAT | Cds2 | 51 kDa | Rattus norvegicus |
| GRZ2_RAT | Mcpt10 | 27 kDa | Rattus norvegicus |
| AN32A_RAT | Anp32a | 29 kDa | Rattus norvegicus |
| KPCA_RAT | Prkca | 77 kDa | Rattus norvegicus |
| RS27A_RAT | Rps27a | 18 kDa | Rattus norvegicus |
| FPRP_RAT | Ptgfrn | 99 kDa | Rattus norvegicus |
| KHDR1_RAT | Khdrbs1 | 48 kDa | Rattus norvegicus |
| M2OM_RAT | Slc25a11 | 34 kDa | Rattus norvegicus |
| IDE_RAT | Ide | 118 kDa | Rattus norvegicus |
| SYNP2_RAT | Synpo2 | 136 kDa | Rattus norvegicus |

| ACADM_RAT | Acadm | 47 kDa | Rattus norvegicus |
| --- | --- | --- | --- |
| RBM8A_RAT | Rbm8a | 20 kDa | Rattus norvegicus |
| RL10_RAT | Rpl10 | 25 kDa | Rattus norvegicus |
| TMED7_RAT | Tmed7 | 25 kDa | Rattus norvegicus |
| PEF1_RAT | Pef1 | 30 kDa | Rattus norvegicus |
| UBA3_RAT | Uba3 | 52 kDa | Rattus norvegicus |
| RL30_RAT | Rpl30 | 13 kDa | Rattus norvegicus |
| IGG2C_RAT |  | 37 kDa | Rattus norvegicus |
| AL3B1_RAT | Aldh3b1 | 52 kDa | Rattus norvegicus |
| DHTK1_RAT | Dhtkd1 | 103 kDa | Rattus norvegicus |
| P2RX4_RAT | P2rx4 | 44 kDa | Rattus norvegicus |
| DHPR_RAT | Qdpr | 26 kDa | Rattus norvegicus |
| S10AB_RAT | S100a11 | 11 kDa | Rattus norvegicus |
| MRP1_RAT | Abcc1 | 171 kDa | Rattus norvegicus |
| NDUA9_RAT | Ndufa9 | 43 kDa | Rattus norvegicus |
| PECR_RAT | Pecr | 32 kDa | Rattus norvegicus |
| GOLI4_RAT | Golim4 | 77 kDa | Rattus norvegicus |
| RS12_RAT | Rps12 | 15 kDa | Rattus norvegicus |
| CPSF5_RAT | Nudt21 | 26 kDa | Rattus norvegicus |
| NCLN_RAT | Ncln | 63 kDa | Rattus norvegicus |
| PP1A_RAT | Ppp1ca | 38 kDa | Rattus norvegicus |
| RAB18_RAT | Rab18 | 23 kDa | Rattus norvegicus |
| HGS_RAT | Hgs | 86 kDa | Rattus norvegicus |
| CSKP_RAT | Cask | 103 kDa | Rattus norvegicus |
| GSTM4_RAT | Gstm3 | 26 kDa | Rattus norvegicus |
| DDC_RAT | Ddc | 54 kDa | Rattus norvegicus |
| SOAT1_RAT | Soat1 | 64 kDa | Rattus norvegicus |
| ILF3_RAT | Ilf3 | 96 kDa | Rattus norvegicus |
| SMU1_RAT | Smu1 | 58 kDa | Rattus norvegicus |
| MIF_RAT | Mif | 12 kDa | Rattus norvegicus |
| OGA_RAT | Oga | 103 kDa | Rattus norvegicus |
| LRC59_RAT | Lrrc59 | 35 kDa | Rattus norvegicus |
| KPCZ_RAT | Prkcz | 68 kDa | Rattus norvegicus |
| AL1A7_RAT | Aldh1a7 | 55 kDa | Rattus norvegicus |
| RISC_RAT | Scpep1 | 51 kDa | Rattus norvegicus |
| SPA3M_RAT | Serpina3m | 46 kDa | Rattus norvegicus |
| ULA1_RAT | Nae1 | 60 kDa | Rattus norvegicus |
| F10A1_RAT | St13 | 41 kDa | Rattus norvegicus |
| NUDC_RAT | Nudc | 38 kDa | Rattus norvegicus |
| ABCF3_RAT | Abcf3 | 80 kDa | Rattus norvegicus |
| DYL1_RAT | Dynll1 | 10 kDa | Rattus norvegicus |
| MUP_RAT |  | 21 kDa | Rattus norvegicus |
| GALT_RAT | Galt | 43 kDa | Rattus norvegicus |
| PCNA_RAT | Pcna | 29 kDa | Rattus norvegicus |
| CD14_RAT | Cd14 | 40 kDa | Rattus norvegicus |
| PIR_RAT | Pir | 32 kDa | Rattus norvegicus |
| SHIP1_RAT | Inpp5d | 134 kDa | Rattus norvegicus |

| MLEC_RAT | Mlec | 32 kDa | Rattus norvegicus |
| --- | --- | --- | --- |
| FBP1L_RAT | Fnbp1l | 70 kDa | Rattus norvegicus |
| ASNS_RAT | Asns | 64 kDa | Rattus norvegicus |
| TIPRL_RAT | Tiprl | 31 kDa | Rattus norvegicus |
| ODO2_RAT | Dlst | 49 kDa | Rattus norvegicus |
| TRIO_RAT | Trio | 348 kDa | Rattus norvegicus |
| ATPG_RAT | Atp5f1c | 30 kDa | Rattus norvegicus |
| BASI_RAT | Bsg | 42 kDa | Rattus norvegicus |
| CORO7_RAT | Coro7 | 101 kDa | Rattus norvegicus |
| L2GL1_RAT | Llgl1 | 112 kDa | Rattus norvegicus |
| CAC1E_RAT | Cacna1e | 252 kDa | Rattus norvegicus |
| AQP1_RAT | Aqp1 | 29 kDa | Rattus norvegicus |
| APMAP_RAT | Apmap | 42 kDa | Rattus norvegicus |
| FBLN5_RAT | Fbln5 | 50 kDa | Rattus norvegicus |
| EIF3G_RAT | Eif3g | 36 kDa | Rattus norvegicus |
| RS13_RAT | Rps13 | 17 kDa | Rattus norvegicus |
| EXOC7_RAT | Exoc7 | 75 kDa | Rattus norvegicus |
| CYTB_RAT | Cstb | 11 kDa | Rattus norvegicus |
| PSB5_RAT | Psmb5 | 29 kDa | Rattus norvegicus |
| GAK_RAT | Gak | 144 kDa | Rattus norvegicus |
| CHIA_RAT | Chia | 52 kDa | Rattus norvegicus |
| TYB4_RAT | Tmsb4x | 5 kDa | Rattus norvegicus |
| RL15_RAT | Rpl15 | 24 kDa | Rattus norvegicus |
| ACOT2_RAT | Acot2 | 50 kDa | Rattus norvegicus |
| RAVR1_RAT | Raver1 | 79 kDa | Rattus norvegicus |
| SH3G1_RAT | Sh3gl1 | 41 kDa | Rattus norvegicus |
| PDE3A_RAT | Pde3a | 124 kDa | Rattus norvegicus |
| CEAM1_RAT | Ceacam1 | 57 kDa | Rattus norvegicus |
| TRXR1_RAT | Txnrd1 | 55 kDa | Rattus norvegicus |
| TOP2A_RAT | Top2a | 173 kDa | Rattus norvegicus |
| DJB11_RAT | Dnajb11 | 40 kDa | Rattus norvegicus |
| NOP58_RAT | Nop58 | 60 kDa | Rattus norvegicus |
| WDR26_RAT | Wdr26 | 58 kDa | Rattus norvegicus |
| TMOD1_RAT | Tmod1 | 40 kDa | Rattus norvegicus |
| GNAO_RAT | Gnao1 | 40 kDa | Rattus norvegicus |
| STIM1_RAT | Stim1 | 77 kDa | Rattus norvegicus |
| CASP6_RAT | Casp6 | 32 kDa | Rattus norvegicus |
| CAH3_RAT | Ca3 | 29 kDa | Rattus norvegicus |
| GRK5_RAT | Grk5 | 68 kDa | Rattus norvegicus |
| EXOC5_RAT | Exoc5 | 82 kDa | Rattus norvegicus |
| GTF2I_RAT | Gtf2i | 110 kDa | Rattus norvegicus |
| RSMN_RAT | Snrpn | 25 kDa | Rattus norvegicus |
| LIMA1_RAT | Lima1 | 84 kDa | Rattus norvegicus |
| KCRU_RAT | Ckmt1 | 47 kDa | Rattus norvegicus |
| UBR5_RAT | Ubr5 | 308 kDa | Rattus norvegicus |
| AN32B_RAT | Anp32b | 31 kDa | Rattus norvegicus |
| SNX27_RAT | Snx27 | 61 kDa | Rattus norvegicus |

| TAP2_RAT | Tap2 | 78 kDa | Rattus norvegicus |
| --- | --- | --- | --- |
| TRAP1_RAT | Trap1 | 80 kDa | Rattus norvegicus |
| DCTN4_RAT | Dctn4 | 53 kDa | Rattus norvegicus |
| ARHG6_RAT | Arhgef6 | 87 kDa | Rattus norvegicus |
| HXK3_RAT | Hk3 | 100 kDa | Rattus norvegicus |
| ATG7_RAT | Atg7 | 77 kDa | Rattus norvegicus |
| KS6A1_RAT | Rps6ka1 | 83 kDa | Rattus norvegicus |
| AIMP2_RAT | Aimp2 | 35 kDa | Rattus norvegicus |
| CTBP1_RAT | Ctbp1 | 47 kDa | Rattus norvegicus |
| MSRA_RAT | Msra | 26 kDa | Rattus norvegicus |
| PDCD4_RAT | Pdcd4 | 52 kDa | Rattus norvegicus |
| PPAC_RAT | Acp1 | 18 kDa | Rattus norvegicus |
| SCLY_RAT | Scly | 47 kDa | Rattus norvegicus |
| UBQL1_RAT | Ubqln1 | 62 kDa | Rattus norvegicus |
| RGPA2_RAT | Ralgapa2 | 210 kDa | Rattus norvegicus |
| PTPRF_RAT | Ptprf | 211 kDa | Rattus norvegicus |
| RRAGA_RAT | Rraga | 37 kDa | Rattus norvegicus |
| ACAP2_RAT | Acap2 | 87 kDa | Rattus norvegicus |
| HMOX2_RAT | Hmox2 | 36 kDa | Rattus norvegicus |
| RS10_RAT | Rps10 | 19 kDa | Rattus norvegicus |
| COQ9_RAT | Coq9 | 35 kDa | Rattus norvegicus |
| TNNT2_RAT | Tnnt2 | 36 kDa | Rattus norvegicus |
| PKN1_RAT | Pkn1 | 104 kDa | Rattus norvegicus |
| PSB4_RAT | Psmb4 | 29 kDa | Rattus norvegicus |
| HEPH_RAT | Heph | 130 kDa | Rattus norvegicus |
| GALK2_RAT | Galk2 | 50 kDa | Rattus norvegicus |
| GFPT1_RAT | Gfpt1 | 77 kDa | Rattus norvegicus |
| NEUA_RAT | Cmas | 48 kDa | Rattus norvegicus |
| CALRL_RAT | Calcrl | 53 kDa | Rattus norvegicus |
| CSDE1_RAT | Csde1 | 89 kDa | Rattus norvegicus |
| RS19_RAT | Rps19 | 16 kDa | Rattus norvegicus |
| SC22B_RAT | Sec22b | 25 kDa | Rattus norvegicus |
| PP2BA_RAT | Ppp3ca | 59 kDa | Rattus norvegicus |
| AMBP_RAT | Ambp | 39 kDa | Rattus norvegicus |
| NUMB_RAT | Numb | 71 kDa | Rattus norvegicus |
| NPT2B_RAT | Slc34a2 | 76 kDa | Rattus norvegicus |
| PRP4B_RAT | Prpf4b | 117 kDa | Rattus norvegicus |
| F13A_RAT | F13a1 | 83 kDa | Rattus norvegicus |
| F234A_RAT | Fam234a | 61 kDa | Rattus norvegicus |
| TAOK1_RAT | Taok1 | 116 kDa | Rattus norvegicus |
| PUR6_RAT | Paics | 47 kDa | Rattus norvegicus |
| ZPI_RAT | Serpina10 | 50 kDa | Rattus norvegicus |
| GRB2_RAT | Grb2 | 25 kDa | Rattus norvegicus |
| RASA3_RAT | Rasa3 | 96 kDa | Rattus norvegicus |
| FXR1_RAT | Fxr1 | 64 kDa | Rattus norvegicus |
| SCRN2_RAT | Scrn2 | 47 kDa | Rattus norvegicus |
| GMPPA_RAT | Gmppa | 46 kDa | Rattus norvegicus |

| NID2_RAT | Nid2 | 153 kDa | Rattus norvegicus |
| --- | --- | --- | --- |
| H31_RAT |  | 15 kDa | Rattus norvegicus |
| EST5_RAT |  | 62 kDa | Rattus norvegicus |
| SET_RAT | Set | 33 kDa | Rattus norvegicus |
| B3A2_RAT | Slc4a2 | 137 kDa | Rattus norvegicus |
| PDZD2_RAT | Pdzd2 | 294 kDa | Rattus norvegicus |
| SE1L1_RAT | Sel1l | 89 kDa | Rattus norvegicus |
| RAB10_RAT | Rab10 | 23 kDa | Rattus norvegicus |
| RABE1_RAT | Rabep1 | 99 kDa | Rattus norvegicus |
| GSTT1_RAT | Gstt1 | 27 kDa | Rattus norvegicus |
| MTNA_RAT | Mri1 | 40 kDa | Rattus norvegicus |
| KNT1_RAT | Map1 | 48 kDa | Rattus norvegicus |
| GOGA2_RAT | Golga2 | 113 kDa | Rattus norvegicus |
| RHG29_RAT | Arhgap29 | 142 kDa | Rattus norvegicus |
| HSDL2_RAT | Hsdl2 | 58 kDa | Rattus norvegicus |
| NDRG1_RAT | Ndrg1 | 43 kDa | Rattus norvegicus |
| PURB_RAT | Purb | 33 kDa | Rattus norvegicus |
| MEMO1_RAT | Memo1 | 34 kDa | Rattus norvegicus |
| ALDOC_RAT | Aldoc | 39 kDa | Rattus norvegicus |
| ECH1_RAT | Ech1 | 36 kDa | Rattus norvegicus |
| GCKR_RAT | Gckr | 69 kDa | Rattus norvegicus |
| GNAI3_RAT | Gnai3 | 41 kDa | Rattus norvegicus |
| SNP23_RAT | Snap23 | 23 kDa | Rattus norvegicus |
| GIT1_RAT | Git1 | 85 kDa | Rattus norvegicus |
| MRCKA_RAT | Cdc42bpa | 197 kDa | Rattus norvegicus |
| TINAL_RAT | Tinagl1 | 53 kDa | Rattus norvegicus |
| ITSN1_RAT | Itsn1 | 194 kDa | Rattus norvegicus |
| PACN2_RAT | Pacsin2 | 56 kDa | Rattus norvegicus |
| AP2S1_RAT | Ap2s1 | 17 kDa | Rattus norvegicus |
| OLFL1_RAT | Olfml1 | 46 kDa | Rattus norvegicus |
| RS17_RAT | Rps17 | 16 kDa | Rattus norvegicus |
| ITPR3_RAT | Itpr3 | 304 kDa | Rattus norvegicus |
| LCAP_RAT | Lnpep | 117 kDa | Rattus norvegicus |
| SGMR1_RAT | Sigmar1 | 25 kDa | Rattus norvegicus |
| FSCN1_RAT | Fscn1 | 54 kDa | Rattus norvegicus |
| DNJA2_RAT | Dnaja2 | 46 kDa | Rattus norvegicus |
| ERF1_RAT | Etf1 | 49 kDa | Rattus norvegicus |
| UBC9_RAT | Ube2i | 18 kDa | Rattus norvegicus |
| SRPRB_RAT | Srprb | 30 kDa | Rattus norvegicus |
| PCY1A_RAT | Pcyt1a | 42 kDa | Rattus norvegicus |
| MPDZ_RAT | Mpdz | 219 kDa | Rattus norvegicus |
| S4A4_RAT | Slc4a4 | 121 kDa | Rattus norvegicus |
| GTPB1_RAT | Gtpbp1 | 72 kDa | Rattus norvegicus |
| NAAA_RAT | Naaa | 40 kDa | Rattus norvegicus |
| ARPC5_RAT | Arpc5 | 16 kDa | Rattus norvegicus |
| SFXN3_RAT | Sfxn3 | 35 kDa | Rattus norvegicus |
| APAF_RAT | Apaf1 | 141 kDa | Rattus norvegicus |

| PUF60_RAT | Puf60 | 60 kDa | Rattus norvegicus |
| --- | --- | --- | --- |
| VWF_RAT | Vwf | 48 kDa | Rattus norvegicus |
| PIEZ1_RAT | Piezo1 | 290 kDa | Rattus norvegicus |
| ADA17_RAT | Adam17 | 93 kDa | Rattus norvegicus |
| GCYA2_RAT | Gucy1a2 | 82 kDa | Rattus norvegicus |
| RL21_RAT | Rpl21 | 18 kDa | Rattus norvegicus |
| UBE2N_RAT | Ube2n | 17 kDa | Rattus norvegicus |
| UBF1_RAT | Ubtf | 89 kDa | Rattus norvegicus |
| SMRCD_RAT | Smarcad1 | 117 kDa | Rattus norvegicus |
| GMFB_RAT | Gmfb | 17 kDa | Rattus norvegicus |
| GNA11_RAT | Gna11 | 42 kDa | Rattus norvegicus |
| STAM2_RAT | Stam2 | 57 kDa | Rattus norvegicus |
| PSB7_RAT | Psmb7 | 30 kDa | Rattus norvegicus |
| BGLR_RAT | Gusb | 75 kDa | Rattus norvegicus |
| PK3C3_RAT | Pik3c3 | 102 kDa | Rattus norvegicus |
| RAD50_RAT | Rad50 | 154 kDa | Rattus norvegicus |
| CFAD_RAT | Cfd | 28 kDa | Rattus norvegicus |
| PI42A_RAT | Pip4k2a | 46 kDa | Rattus norvegicus |
| UBP4_RAT | Usp4 | 108 kDa | Rattus norvegicus |
| RB6I2_RAT | Erc1 | 109 kDa | Rattus norvegicus |
| ODBB_RAT | Bckdhb | 43 kDa | Rattus norvegicus |
| VPS45_RAT | Vps45 | 65 kDa | Rattus norvegicus |
| ZFR_RAT | Zfr | 117 kDa | Rattus norvegicus |
| AAKG1_RAT | Prkag1 | 37 kDa | Rattus norvegicus |
| SAHH2_RAT | Ahcyl1 | 54 kDa | Rattus norvegicus |
| NNRE_RAT | Naxe | 31 kDa | Rattus norvegicus |
| QKI_RAT | Qki | 38 kDa | Rattus norvegicus |
| NSF1C_RAT | Nsfl1c | 41 kDa | Rattus norvegicus |
| TAOK3_RAT | Taok3 | 105 kDa | Rattus norvegicus |
| CLIC2_RAT | Clic2 | 28 kDa | Rattus norvegicus |
| TIM44_RAT | Timm44 | 51 kDa | Rattus norvegicus |
| AN32E_RAT | Anp32e | 29 kDa | Rattus norvegicus |
| PDCD6_RAT | Pdcd6 | 22 kDa | Rattus norvegicus |
| DHB11_RAT | Hsd17b11 | 33 kDa | Rattus norvegicus |
| CD48_RAT | Cd48 | 28 kDa | Rattus norvegicus |
| NQO1_RAT | Nqo1 | 31 kDa | Rattus norvegicus |
| MAOX_RAT | Me1 | 64 kDa | Rattus norvegicus |
| RBM3_RAT | Rbm3 | 17 kDa | Rattus norvegicus |
| CSPG2_RAT | Vcan | 300 kDa | Rattus norvegicus |
| COG7_RAT | Cog7 | 86 kDa | Rattus norvegicus |
| RS5_RAT | Rps5 | 23 kDa | Rattus norvegicus |
| AOXA_RAT | Aox1 | 147 kDa | Rattus norvegicus |
| KPRA_RAT | Prpsap1 | 39 kDa | Rattus norvegicus |
| CX6C2_RAT | Cox6c2 | 8 kDa | Rattus norvegicus |
| MK14_RAT | Mapk14 | 41 kDa | Rattus norvegicus |
| VP35L_RAT | Vps35l | 106 kDa | Rattus norvegicus |
| ZA2G_RAT | Azgp1 | 34 kDa | Rattus norvegicus |

| PEA15_RAT | Pea15 | 15 kDa | Rattus norvegicus |
| --- | --- | --- | --- |
| STRN_RAT | Strn | 86 kDa | Rattus norvegicus |
| ABHEB_RAT | Abhd14b | 23 kDa | Rattus norvegicus |
| KAPCB_RAT | Prkacb | 41 kDa | Rattus norvegicus |
| PTGR2_RAT | Ptgr2 | 38 kDa | Rattus norvegicus |
| MPP7_RAT | Mpp7 | 66 kDa | Rattus norvegicus |
| RL22_RAT | Rpl22 | 15 kDa | Rattus norvegicus |
| PFKAM_RAT | Pfkm | 86 kDa | Rattus norvegicus |
| KPCD_RAT | Prkcd | 78 kDa | Rattus norvegicus |
| RL11_RAT | Rpl11 | 20 kDa | Rattus norvegicus |
| ODBA_RAT | Bckdha | 50 kDa | Rattus norvegicus |
| DTNB_RAT | Dtnb | 74 kDa | Rattus norvegicus |
| E41L1_RAT | Epb41l1 | 98 kDa | Rattus norvegicus |
| ARLY_RAT | Asl | 52 kDa | Rattus norvegicus |
| BZW1_RAT | Bzw1 | 48 kDa | Rattus norvegicus |
| ELP1_RAT | Elp1 | 149 kDa | Rattus norvegicus |
| PDLI1_RAT | Pdlim1 | 36 kDa | Rattus norvegicus |
| PLAP_RAT | Plaa | 87 kDa | Rattus norvegicus |
| ADT2_RAT | Slc25a5 | 33 kDa | Rattus norvegicus |
| PGFRB_RAT | Pdgfrb | 123 kDa | Rattus norvegicus |
| MYO9B_RAT | Myo9b | 225 kDa | Rattus norvegicus |
| HEM3_RAT | Hmbs | 39 kDa | Rattus norvegicus |
| SRSF2_RAT | Srsf2 | 25 kDa | Rattus norvegicus |
| TRYB1_RAT | Tpsab1 | 30 kDa | Rattus norvegicus |
| SDCB1_RAT | Sdcbp | 32 kDa | Rattus norvegicus |
| GALM_RAT | Galm | 38 kDa | Rattus norvegicus |
| HCK_RAT | Hck | 59 kDa | Rattus norvegicus |
| SGPL1_RAT | Sgpl1 | 64 kDa | Rattus norvegicus |
| GLYG_RAT | Gyg1 | 37 kDa | Rattus norvegicus |
| CPPED_RAT | Cpped1 | 35 kDa | Rattus norvegicus |
| PSA3_RAT | Psma3 | 28 kDa | Rattus norvegicus |
| VP33A_RAT | Vps33a | 68 kDa | Rattus norvegicus |
| ZCCHV_RAT | Zc3hav1 | 87 kDa | Rattus norvegicus |
| PSPC1_RAT | Pspc1 | 59 kDa | Rattus norvegicus |
| CASP1_RAT | Casp1 | 46 kDa | Rattus norvegicus |
| TOM22_RAT | Tomm22 | 15 kDa | Rattus norvegicus |
| STRN3_RAT | Strn3 | 87 kDa | Rattus norvegicus |
| AMPD3_RAT | Ampd3 | 88 kDa | Rattus norvegicus |
| STML2_RAT | Stoml2 | 38 kDa | Rattus norvegicus |
| PRRC1_RAT | Prrc1 | 46 kDa | Rattus norvegicus |
| RLA1_RAT | Rplp1 | 11 kDa | Rattus norvegicus |
| ICAL_RAT | Cast | 77 kDa | Rattus norvegicus |
| IL6RB_RAT | Il6st | 102 kDa | Rattus norvegicus |
| WNK1_RAT | Wnk1 | 225 kDa | Rattus norvegicus |
| CHRD1_RAT | Chordc1 | 37 kDa | Rattus norvegicus |
| NAGK_RAT | Nagk | 37 kDa | Rattus norvegicus |
| TS101_RAT | Tsg101 | 44 kDa | Rattus norvegicus |

| VAC14_RAT | Vac14 | 88 kDa | Rattus norvegicus |
| --- | --- | --- | --- |
| AGRL3_RAT | Adgrl3 | 172 kDa | Rattus norvegicus |
| NPTN_RAT | Nptn | 44 kDa | Rattus norvegicus |
| RB3GP_RAT | Rab3gap1 | 87 kDa | Rattus norvegicus |
| 5NTD_RAT | Nt5e | 64 kDa | Rattus norvegicus |
| SSRD_RAT | Ssr4 | 19 kDa | Rattus norvegicus |
| UBCP1_RAT | Ublcp1 | 37 kDa | Rattus norvegicus |
| RS20_RAT | Rps20 | 13 kDa | Rattus norvegicus |
| SO2A1_RAT | Slco2a1 | 70 kDa | Rattus norvegicus |
| TBG1_RAT | Tubg1 | 51 kDa | Rattus norvegicus |
| GRPE1_RAT | Grpel1 | 24 kDa | Rattus norvegicus |
| EXOC3_RAT | Exoc3 | 86 kDa | Rattus norvegicus |
| LAMP3_RAT | Lamp3 | 44 kDa | Rattus norvegicus |
| EF1A2_RAT | Eef1a2 | 50 kDa | Rattus norvegicus |
| ELOC_RAT | Eloc | 12 kDa | Rattus norvegicus |
| PRKRA_RAT | Prkra | 34 kDa | Rattus norvegicus |
| BROX_RAT | Brox | 46 kDa | Rattus norvegicus |
| LMOD1_RAT | Lmod1 | 66 kDa | Rattus norvegicus |
| VATC1_RAT | Atp6v1c1 | 44 kDa | Rattus norvegicus |
| RS16_RAT | Rps16 | 16 kDa | Rattus norvegicus |
| RCN2_RAT | Rcn2 | 37 kDa | Rattus norvegicus |
| SNUT1_RAT | Sart1 | 91 kDa | Rattus norvegicus |
| TPP1_RAT | Tpp1 | 61 kDa | Rattus norvegicus |
| DPP2_RAT | Dpp7 | 55 kDa | Rattus norvegicus |
| NISCH_RAT | Nisch | 167 kDa | Rattus norvegicus |
| CD1D_RAT | Cd1d | 39 kDa | Rattus norvegicus |
| ARF5_RAT | Arf5 | 21 kDa | Rattus norvegicus |
| IRGM_RAT | Irgm | 46 kDa | Rattus norvegicus |
| UBA5_RAT | Uba5 | 45 kDa | Rattus norvegicus |
| VIPR1_RAT | Vipr1 | 52 kDa | Rattus norvegicus |
| RASA1_RAT | Rasa1 | 115 kDa | Rattus norvegicus |
| K2C1_RAT | Krt1 | 65 kDa | Rattus norvegicus |
| CBPA3_RAT | Cpa3 | 48 kDa | Rattus norvegicus |
| NMRL1_RAT | Nmral1 | 17 kDa | Rattus norvegicus |
| S14L2_RAT | Sec14l2 | 46 kDa | Rattus norvegicus |
| TOLIP_RAT | Tollip | 30 kDa | Rattus norvegicus |
| LAMP1_RAT | Lamp1 | 44 kDa | Rattus norvegicus |
| TEP1_RAT | Tep1 | 292 kDa | Rattus norvegicus |
| ENOPH_RAT | Enoph1 | 29 kDa | Rattus norvegicus |
| CH10_RAT | Hspe1 | 11 kDa | Rattus norvegicus |
| TBA3_RAT | Tuba3a | 50 kDa | Rattus norvegicus |
| MAGI1_RAT | Magi1 | 136 kDa | Rattus norvegicus |
| DYHC2_RAT | Dync2h1 | 492 kDa | Rattus norvegicus |
| MPP2_RAT | Mpp2 | 62 kDa | Rattus norvegicus |
| ARK73_RAT | Akr7a3 | 37 kDa | Rattus norvegicus |
| NRDC_RAT | Nrdc | 133 kDa | Rattus norvegicus |
| CIRBP_RAT | Cirbp | 19 kDa | Rattus norvegicus |

| GNPAT_RAT | Gnpat | 77 kDa | Rattus norvegicus |
| --- | --- | --- | --- |
| S10A8_RAT | S100a8 | 10 kDa | Rattus norvegicus |
| 3BP1_RAT | Sh3bp1 | 75 kDa | Rattus norvegicus |
| RER1_RAT | Rer1 | 23 kDa | Rattus norvegicus |
| WDR91_RAT | Wdr91 | 83 kDa | Rattus norvegicus |
| WASL_RAT | Wasl | 54 kDa | Rattus norvegicus |
| BASP1_RAT | Basp1 | 22 kDa | Rattus norvegicus |
| RHG35_RAT | Arhgap35 | 170 kDa | Rattus norvegicus |
| HDGR3_RAT | Hdgfl3 | 22 kDa | Rattus norvegicus |
| HMGCL_RAT | Hmgcl | 34 kDa | Rattus norvegicus |
| PPCEL_RAT | Prepl | 83 kDa | Rattus norvegicus |
| GALNS_RAT | Galns | 58 kDa | Rattus norvegicus |
| GPX4_RAT | Gpx4 | 22 kDa | Rattus norvegicus |
| PLBL1_RAT | Plbd1 | 63 kDa | Rattus norvegicus |
| CSN8_RAT | Cops8 | 23 kDa | Rattus norvegicus |
| ACDSB_RAT | Acadsb | 48 kDa | Rattus norvegicus |
| MARE2_RAT | Mapre2 | 37 kDa | Rattus norvegicus |
| THIC_RAT | Acat2 | 41 kDa | Rattus norvegicus |
| DAB2P_RAT | Dab2ip | 110 kDa | Rattus norvegicus |
| CUX1_RAT | Cux1 | 165 kDa | Rattus norvegicus |
| CAND2_RAT | Cand2 | 140 kDa | Rattus norvegicus |
| RHG44_RAT | Arhgap44 | 89 kDa | Rattus norvegicus |
| MTMR6_RAT | Mtmr6 | 76 kDa | Rattus norvegicus |
| AMPD2_RAT | Ampd2 | 95 kDa | Rattus norvegicus |
| BAF_RAT | Banf1 | 10 kDa | Rattus norvegicus |
| TXD12_RAT | Txndc12 | 19 kDa | Rattus norvegicus |
| PLXA3_RAT | Plxna3 | 208 kDa | Rattus norvegicus |
| ABCA5_RAT | Abca5 | 186 kDa | Rattus norvegicus |
| DHB12_RAT | Hsd17b12 | 35 kDa | Rattus norvegicus |
| RS4X_RAT | Rps4x | 30 kDa | Rattus norvegicus |
| CD47_RAT | Cd47 | 33 kDa | Rattus norvegicus |
| DNMT1_RAT | Dnmt1 | 183 kDa | Rattus norvegicus |
| FA98A_RAT | Fam98a | 55 kDa | Rattus norvegicus |
| ETFD_RAT | Etfdh | 68 kDa | Rattus norvegicus |
| RIPR1_RAT | Ripor1 | 132 kDa | Rattus norvegicus |
| PDLI7_RAT | Pdlim7 | 50 kDa | Rattus norvegicus |
| S10A9_RAT | S100a9 | 13 kDa | Rattus norvegicus |
| PDIA5_RAT | Pdia5 | 59 kDa | Rattus norvegicus |
| GILT_RAT | Ifi30 | 28 kDa | Rattus norvegicus |
| ACADS_RAT | Acads | 45 kDa | Rattus norvegicus |
| TSNAX_RAT | Tsnax | 33 kDa | Rattus norvegicus |
| PBIP1_RAT | Pbxip1 | 80 kDa | Rattus norvegicus |
| CYH3_RAT | Cyth3 | 46 kDa | Rattus norvegicus |
| KACA_RAT |  | 12 kDa | Rattus norvegicus |
| LYRIC_RAT | Mtdh | 64 kDa | Rattus norvegicus |
| CD38_RAT | Cd38 | 34 kDa | Rattus norvegicus |
| GATM_RAT | Gatm | 48 kDa | Rattus norvegicus |

| VAMP7_RAT | Vamp7 | 25 kDa | Rattus norvegicus |
| --- | --- | --- | --- |
| SF3A2_RAT | Sf3a2 | 50 kDa | Rattus norvegicus |
| CRNL1_RAT | Crnkl1 | 83 kDa | Rattus norvegicus |
| MK12_RAT | Mapk12 | 42 kDa | Rattus norvegicus |
| FABP4_RAT | Fabp4 | 15 kDa | Rattus norvegicus |
| MYH7_RAT | Myh7 | 223 kDa | Rattus norvegicus |
| HEXB_RAT | Hexb | 62 kDa | Rattus norvegicus |
| RMD3_RAT | Rmdn3 | 52 kDa | Rattus norvegicus |
| PDLI5_RAT | Pdlim5 | 63 kDa | Rattus norvegicus |
| SC11A_RAT | Sec11a | 21 kDa | Rattus norvegicus |
| AKT1_RAT | Akt1 | 56 kDa | Rattus norvegicus |
| MRE11_RAT | Mre11 | 80 kDa | Rattus norvegicus |
| NDUS2_RAT | Ndufs2 | 53 kDa | Rattus norvegicus |
| AAKB1_RAT | Prkab1 | 30 kDa | Rattus norvegicus |
| TXTP_RAT | Slc25a1 | 34 kDa | Rattus norvegicus |
| H10_RAT | H1f0 | 21 kDa | Rattus norvegicus |
| KC1A_RAT | Csnk1a1 | 37 kDa | Rattus norvegicus |
| E2AK2_RAT | Eif2ak2 | 58 kDa | Rattus norvegicus |
| RLGPB_RAT | Ralgapb | 165 kDa | Rattus norvegicus |
| EXOC8_RAT | Exoc8 | 81 kDa | Rattus norvegicus |
| ATRN_RAT | Atrn | 159 kDa | Rattus norvegicus |
| CHID1_RAT | Chid1 | 45 kDa | Rattus norvegicus |
| VAPB_RAT | Vapb | 27 kDa | Rattus norvegicus |
| H2AZ_RAT | H2afz | 14 kDa | Rattus norvegicus |
| SPRE_RAT | Spr | 28 kDa | Rattus norvegicus |
| CD81_RAT | Cd81 | 26 kDa | Rattus norvegicus |
| LGUL_RAT | Glo1 | 21 kDa | Rattus norvegicus |
| ARHGB_RAT | Arhgef11 | 169 kDa | Rattus norvegicus |
| SARNP_RAT | Sarnp | 24 kDa | Rattus norvegicus |
| AVIL_RAT | Avil | 93 kDa | Rattus norvegicus |
| PRPS2_RAT | Prps2 | 35 kDa | Rattus norvegicus |
| TBA4A_RAT | Tuba4a | 50 kDa | Rattus norvegicus |
| CELF2_RAT | Celf2 | 54 kDa | Rattus norvegicus |
| MARK3_RAT | Mark3 | 89 kDa | Rattus norvegicus |
| DEK_RAT | Dek | 43 kDa | Rattus norvegicus |
| RS8_RAT | Rps8 | 24 kDa | Rattus norvegicus |
| RAC1_RAT | Rac1 | 21 kDa | Rattus norvegicus |
| TOIP1_RAT | Tor1aip1 | 66 kDa | Rattus norvegicus |
| BAIP2_RAT | Baiap2 | 59 kDa | Rattus norvegicus |
| NDUAA_RAT | Ndufa10 | 40 kDa | Rattus norvegicus |
| MARK2_RAT | Mark2 | 81 kDa | Rattus norvegicus |
| SRC_RAT | Src | 60 kDa | Rattus norvegicus |
| EI2BE_RAT | Eif2b5 | 80 kDa | Rattus norvegicus |
| CD2AP_RAT | Cd2ap | 70 kDa | Rattus norvegicus |
| STK10_RAT | Stk10 | 112 kDa | Rattus norvegicus |
| LIPS_RAT | Lipe | 117 kDa | Rattus norvegicus |
| CL2DB_RAT | Clec2d11 | 24 kDa | Rattus norvegicus |

| PRAF3_RAT | Arl6ip5 | 22 kDa | Rattus norvegicus |
| --- | --- | --- | --- |
| PR38B_RAT | Prpf38b | 64 kDa | Rattus norvegicus |
| METH_RAT | Mtr | 139 kDa | Rattus norvegicus |
| AT2A1_RAT | Atp2a1 | 109 kDa | Rattus norvegicus |
| CP2D4_RAT | Cyp2d4 | 57 kDa | Rattus norvegicus |
| NLRX1_RAT | Nlrx1 | 108 kDa | Rattus norvegicus |
| SBDS_RAT | Sbds | 29 kDa | Rattus norvegicus |
| EIPR1_RAT | Eipr1 | 43 kDa | Rattus norvegicus |
| LRP4_RAT | Lrp4 | 212 kDa | Rattus norvegicus |
| SUOX_RAT | Suox | 61 kDa | Rattus norvegicus |
| VPS4A_RAT | Vps4a | 49 kDa | Rattus norvegicus |
| AIP_RAT | Aip | 38 kDa | Rattus norvegicus |
| CISD1_RAT | Cisd1 | 12 kDa | Rattus norvegicus |
| GMPR1_RAT | Gmpr | 37 kDa | Rattus norvegicus |
| RS9_RAT | Rps9 | 23 kDa | Rattus norvegicus |
| EPN2_RAT | Epn2 | 62 kDa | Rattus norvegicus |
| INP4A_RAT | Inpp4a | 106 kDa | Rattus norvegicus |
| FCHO2_RAT | Fcho2 | 89 kDa | Rattus norvegicus |
| CDC5L_RAT | Cdc5l | 92 kDa | Rattus norvegicus |
| ALD1_RAT | Akr1b7 | 36 kDa | Rattus norvegicus |
| IMA5_RAT | Kpna1 | 60 kDa | Rattus norvegicus |
| MINY1_RAT | Mindy1 | 53 kDa | Rattus norvegicus |
| GNL1_RAT | Gnl1 | 69 kDa | Rattus norvegicus |
| VPS36_RAT | Vps36 | 44 kDa | Rattus norvegicus |
| COPG2_RAT | Copg2 | 80 kDa | Rattus norvegicus |
| HPBP1_RAT | Hspbp1 | 39 kDa | Rattus norvegicus |
| EMB_RAT | Emb | 37 kDa | Rattus norvegicus |
| GSTA6_RAT | Gsta6 | 26 kDa | Rattus norvegicus |
| SPTC2_RAT | Sptlc2 | 63 kDa | Rattus norvegicus |
| PPP6_RAT | Ppp6c | 35 kDa | Rattus norvegicus |
| ARFP1_RAT | Arfip1 | 41 kDa | Rattus norvegicus |
| THNS2_RAT | Thnsl2 | 54 kDa | Rattus norvegicus |
| CD151_RAT | Cd151 | 28 kDa | Rattus norvegicus |
| RAB8B_RAT | Rab8b | 24 kDa | Rattus norvegicus |
| TRA2B_RAT | Tra2b | 34 kDa | Rattus norvegicus |
| TNNI3_RAT | Tnni3 | 24 kDa | Rattus norvegicus |
| BRCC3_RAT | Brcc3 | 33 kDa | Rattus norvegicus |
| DOCK9_RAT | Dock9 | 82 kDa | Rattus norvegicus |
| EXOC6_RAT | Exoc6 | 93 kDa | Rattus norvegicus |
| GRAM_RAT | Gzmm | 28 kDa | Rattus norvegicus |
| ZC3HF_RAT | Zc3h15 | 48 kDa | Rattus norvegicus |
| AAK1_RAT | Aak1 | 104 kDa | Rattus norvegicus |
| UBXN4_RAT | Ubxn4 | 56 kDa | Rattus norvegicus |
| RS26_RAT | Rps26 | 13 kDa | Rattus norvegicus |
| RAP1A_RAT | Rap1a | 21 kDa | Rattus norvegicus |
| CPT1A_RAT | Cpt1a | 88 kDa | Rattus norvegicus |
| VGFR2_RAT | Kdr | 150 kDa | Rattus norvegicus |

| CBPD_RAT | Cpd | 153 kDa | Rattus norvegicus |
| --- | --- | --- | --- |
| FNBP1_RAT | Fnbp1 | 71 kDa | Rattus norvegicus |
| GLTP_RAT | GLTP | 24 kDa | Rattus norvegicus |
| MAP1S_RAT | Map1s | 103 kDa | Rattus norvegicus |
| MACD1_RAT | Macrod1 | 29 kDa | Rattus norvegicus |
| PALM_RAT | Palm | 42 kDa | Rattus norvegicus |
| AQP5_RAT | Aqp5 | 28 kDa | Rattus norvegicus |
| ERG7_RAT | Lss | 83 kDa | Rattus norvegicus |
| PI42B_RAT | Pip4k2b | 47 kDa | Rattus norvegicus |
| FMR1_RAT | Fmr1 | 67 kDa | Rattus norvegicus |
| CDIPT_RAT | Cdipt | 24 kDa | Rattus norvegicus |
| MINP1_RAT | Minpp1 | 55 kDa | Rattus norvegicus |
| PANK4_RAT | Pank4 | 86 kDa | Rattus norvegicus |
| CK5P3_RAT | Cdk5rap3 | 57 kDa | Rattus norvegicus |
| CATL1_RAT | Ctsl | 38 kDa | Rattus norvegicus |
| NONO_RAT | Nono | 55 kDa | Rattus norvegicus |
| MAT2B_RAT | Mat2b | 37 kDa | Rattus norvegicus |
| UD16_RAT | Ugt1a6 | 60 kDa | Rattus norvegicus |
| STX4_RAT | Stx4 | 34 kDa | Rattus norvegicus |
| PSB9_RAT | Psmb9 | 23 kDa | Rattus norvegicus |
| LOX15_RAT | Alox15 | 75 kDa | Rattus norvegicus |
| MX3_RAT | Mx3 | 75 kDa | Rattus norvegicus |
| GALE_RAT | Gale | 38 kDa | Rattus norvegicus |
| IMPA1_RAT | Impa1 | 31 kDa | Rattus norvegicus |
| PTPRE_RAT | Ptpre | 81 kDa | Rattus norvegicus |
| MTA1_RAT | Mta1 | 79 kDa | Rattus norvegicus |
| NOSTN_RAT | Nostrin | 57 kDa | Rattus norvegicus |
| CP2A3_RAT | Cyp2a3 | 57 kDa | Rattus norvegicus |
| FNTA_RAT | Fnta | 44 kDa | Rattus norvegicus |
| PHOCN_RAT | Mob4 | 26 kDa | Rattus norvegicus |
| COMD3_RAT | Commd3 | 22 kDa | Rattus norvegicus |
| NT5D2_RAT | Nt5dc2 | 64 kDa | Rattus norvegicus |
| VPP1_RAT | Atp6v0a1 | 96 kDa | Rattus norvegicus |
| NUDT5_RAT | Nudt5 | 24 kDa | Rattus norvegicus |
| SODE_RAT | Sod3 | 27 kDa | Rattus norvegicus |
| LOX12_RAT | Alox12 | 76 kDa | Rattus norvegicus |
| PI42C_RAT | Pip4k2c | 47 kDa | Rattus norvegicus |
| DNJC3_RAT | Dnajc3 | 58 kDa | Rattus norvegicus |
| CYGB_RAT | Cygb | 21 kDa | Rattus norvegicus |
| PTER_RAT | Pter | 39 kDa | Rattus norvegicus |
| ACOX3_RAT | Acox3 | 78 kDa | Rattus norvegicus |
| HMCS2_RAT | Hmgcs2 | 57 kDa | Rattus norvegicus |
| ADH7_RAT | Adh7 | 40 kDa | Rattus norvegicus |
| AT1A2_RAT | Atp1a2 | 112 kDa | Rattus norvegicus |
| A2MG_RAT | A2m | 164 kDa | Rattus norvegicus |
| BPNT1_RAT | Bpnt1 | 33 kDa | Rattus norvegicus |
| NSDHL_RAT | Nsdhl | 40 kDa | Rattus norvegicus |

| SSRP1_RAT | Ssrp1 | 81 kDa | Rattus norvegicus |
| --- | --- | --- | --- |
| TCPR1_RAT | Tecpr1 | 130 kDa | Rattus norvegicus |
| P85A_RAT | Pik3r1 | 84 kDa | Rattus norvegicus |
| SRGP2_RAT | Srgap2 | 121 kDa | Rattus norvegicus |
| TRPV2_RAT | Trpv2 | 87 kDa | Rattus norvegicus |
| RB27B_RAT | Rab27b | 25 kDa | Rattus norvegicus |
| DYN3_RAT | Dnm3 | 98 kDa | Rattus norvegicus |
| PTPRA_RAT | Ptpra | 90 kDa | Rattus norvegicus |
| LTOR1_RAT | Lamtor1 | 18 kDa | Rattus norvegicus |
| SARDH_RAT | Sardh | 101 kDa | Rattus norvegicus |
| PGRC2_RAT | Pgrmc2 | 23 kDa | Rattus norvegicus |
| UB2D2_RAT | Ube2d2 | 17 kDa | Rattus norvegicus |
| BIG2_RAT | Arfgef2 | 202 kDa | Rattus norvegicus |
| MRP3_RAT | Abcc3 | 169 kDa | Rattus norvegicus |
| OGT1_RAT | Ogt | 116 kDa | Rattus norvegicus |
| GSH0_RAT | Gclm | 31 kDa | Rattus norvegicus |
| NCALD_RAT | Ncald | 22 kDa | Rattus norvegicus |
| TMED9_RAT | Tmed9 | 27 kDa | Rattus norvegicus |
| ARG28_RAT | Arhgef28 | 191 kDa | Rattus norvegicus |
| GSHB_RAT | Gss | 52 kDa | Rattus norvegicus |
| AT1B1_RAT | Atp1b1 | 35 kDa | Rattus norvegicus |
| NU153_RAT | Nup153 | 153 kDa | Rattus norvegicus |
| CCD93_RAT | Ccdc93 | 73 kDa | Rattus norvegicus |
| GAS6_RAT | Gas6 | 75 kDa | Rattus norvegicus |
| GRAP1_RAT | Gripap1 | 96 kDa | Rattus norvegicus |
| RL27_RAT | Rpl27 | 16 kDa | Rattus norvegicus |
| EOGT_RAT | Eogt | 62 kDa | Rattus norvegicus |
| ARBK1_RAT | Grk2 | 80 kDa | Rattus norvegicus |
| VPS52_RAT | Vps52 | 82 kDa | Rattus norvegicus |
| ATRX_RAT | Atrx | 59 kDa | Rattus norvegicus |
| A1BG_RAT | A1bg | 56 kDa | Rattus norvegicus |
| LARP7_RAT | Larp7 | 65 kDa | Rattus norvegicus |
| PTN1_RAT | Ptpn1 | 50 kDa | Rattus norvegicus |
| PXL2A_RAT | Prxl2a | 26 kDa | Rattus norvegicus |
| KAD3_RAT | Ak3 | 25 kDa | Rattus norvegicus |
| C1QB_RAT | C1qb | 27 kDa | Rattus norvegicus |
| P4HA1_RAT | P4ha1 | 61 kDa | Rattus norvegicus |
| TMED2_RAT | Tmed2 | 23 kDa | Rattus norvegicus |
| OFUT1_RAT | Pofut1 | 45 kDa | Rattus norvegicus |
| GTR3_RAT | Slc2a3 | 54 kDa | Rattus norvegicus |
| TOM70_RAT | Tomm70 | 67 kDa | Rattus norvegicus |
| SMCE1_RAT | Smarce1 | 43 kDa | Rattus norvegicus |
| CMA1_RAT | Cma1 | 28 kDa | Rattus norvegicus |
| MESD_RAT | Mesd | 25 kDa | Rattus norvegicus |
| ZCH18_RAT | Zc3h18 | 106 kDa | Rattus norvegicus |
| LEG9_RAT | Lgals9 | 40 kDa | Rattus norvegicus |
| CSK2B_RAT | Csnk2b | 25 kDa | Rattus norvegicus |

| GSK3A_RAT | Gsk3a | 51 kDa | Rattus norvegicus |
| --- | --- | --- | --- |
| PHLD_RAT | Gpld1 | 94 kDa | Rattus norvegicus |
| LRRF2_RAT | Lrrfip2 | 50 kDa | Rattus norvegicus |
| RN123_RAT | Rnf123 | 149 kDa | Rattus norvegicus |
| MERL_RAT | Nf2 | 69 kDa | Rattus norvegicus |
| ASCC3_RAT | Ascc3 | 250 kDa | Rattus norvegicus |
| PLOD3_RAT | Plod3 | 85 kDa | Rattus norvegicus |
| CP20A_RAT | Cyp20a1 | 52 kDa | Rattus norvegicus |
| VCIP1_RAT | Vcpip1 | 135 kDa | Rattus norvegicus |
| NOG1_RAT | Gtpbp4 | 74 kDa | Rattus norvegicus |
| APC5_RAT | Anapc5 | 82 kDa | Rattus norvegicus |
| RD23B_RAT | Rad23b | 43 kDa | Rattus norvegicus |
| WASC2_RAT | Washc2 | 145 kDa | Rattus norvegicus |
| GYS1_RAT | Gys1 | 84 kDa | Rattus norvegicus |
| AF1L1_RAT | Afap1l1 | 87 kDa | Rattus norvegicus |
| FLRT3_RAT | Flrt3 | 73 kDa | Rattus norvegicus |
| CBPB2_RAT | Cpb2 | 49 kDa | Rattus norvegicus |
| STX2_RAT | Stx2 | 33 kDa | Rattus norvegicus |
| FAK2_RAT | Ptk2b | 116 kDa | Rattus norvegicus |
| QOR_RAT | Cryz | 35 kDa | Rattus norvegicus |
| SYCP1_RAT | Sycp1 | 117 kDa | Rattus norvegicus |
| PLS3_RAT | Plscr3 | 32 kDa | Rattus norvegicus |
| BABA2_RAT | Babam2 | 44 kDa | Rattus norvegicus |
| MEP50_RAT | Wdr77 | 37 kDa | Rattus norvegicus |
| UFC1_RAT | Ufc1 | 19 kDa | Rattus norvegicus |
| RAF1_RAT | Raf1 | 73 kDa | Rattus norvegicus |
| SH3B4_RAT | Sh3bp4 | 108 kDa | Rattus norvegicus |
| NEST_RAT | Nes | 209 kDa | Rattus norvegicus |
| ALAT1_RAT | Gpt | 55 kDa | Rattus norvegicus |
| FRMD8_RAT | Frmd8 | 52 kDa | Rattus norvegicus |
| PROS_RAT | Pros1 | 75 kDa | Rattus norvegicus |
| IF4E_RAT | Eif4e | 25 kDa | Rattus norvegicus |
| CC90B_RAT | Ccdc90b | 30 kDa | Rattus norvegicus |
| RBM47_RAT | Rbm47 | 64 kDa | Rattus norvegicus |
| HXK2_RAT | Hk2 | 103 kDa | Rattus norvegicus |
| TPC1_RAT | Tpcn1 | 94 kDa | Rattus norvegicus |
| SMRD2_RAT | Smarcd2 | 59 kDa | Rattus norvegicus |
| KPCE_RAT | Prkce | 83 kDa | Rattus norvegicus |
| TGON3_RAT | Ttgn1 | 38 kDa | Rattus norvegicus |
| ERD21_RAT | Kdelr1 | 25 kDa | Rattus norvegicus |
| HG2A_RAT | Cd74 | 32 kDa | Rattus norvegicus |
| TMLH_RAT | Tmlhe | 50 kDa | Rattus norvegicus |
| FIS1_RAT | Fis1 | 17 kDa | Rattus norvegicus |
| SSBP_RAT | Ssbp1 | 17 kDa | Rattus norvegicus |
| PLBL2_RAT | Plbd2 | 65 kDa | Rattus norvegicus |
| CYBC1_RAT | Cybc1 | 21 kDa | Rattus norvegicus |
| NAA25_RAT | Naa25 | 111 kDa | Rattus norvegicus |

| NECP2_RAT | Necap2 | 28 kDa | Rattus norvegicus |
| --- | --- | --- | --- |
| FEN1_RAT | Fen1 | 43 kDa | Rattus norvegicus |
| CTBL1_RAT | Ctnnbl1 | 65 kDa | Rattus norvegicus |
| NDUAB_RAT | Ndufa11 | 15 kDa | Rattus norvegicus |
| TECR_RAT | Tecr | 36 kDa | Rattus norvegicus |
| AS3MT_RAT | As3mt | 41 kDa | Rattus norvegicus |
| SNX3_RAT | Snx3 | 19 kDa | Rattus norvegicus |
| PAG15_RAT | Pla2g15 | 47 kDa | Rattus norvegicus |
| S12A9_RAT | Slc12a9 | 96 kDa | Rattus norvegicus |
| HA11_RAT | RT1-Aw2 | 37 kDa | Rattus norvegicus |
| KCRM_RAT | Ckm | 43 kDa | Rattus norvegicus |
| KCC1A_RAT | Camk1 | 42 kDa | Rattus norvegicus |
| ACY3_RAT | Acy3 | 35 kDa | Rattus norvegicus |
| NDUV2_RAT | Ndufv2 | 27 kDa | Rattus norvegicus |
| ADRM1_RAT | Adrm1 | 42 kDa | Rattus norvegicus |
| RHG07_RAT | Dlc1 | 123 kDa | Rattus norvegicus |
| STPAP_RAT | Tut1 | 94 kDa | Rattus norvegicus |
| LIMD1_RAT | Limd1 | 71 kDa | Rattus norvegicus |
| UBP19_RAT | Usp19 | 150 kDa | Rattus norvegicus |
| CLIP2_RAT | Clip2 | 115 kDa | Rattus norvegicus |
| MBOA5_RAT | Lpcat3 | 56 kDa | Rattus norvegicus |
| AGRL2_RAT | Adgrl2 | 167 kDa | Rattus norvegicus |
| PSB6_RAT | Psmb6 | 25 kDa | Rattus norvegicus |
| NPL_RAT | Npl | 35 kDa | Rattus norvegicus |
| TBB3_RAT | Tubb3 | 50 kDa | Rattus norvegicus |
| RS18_RAT | Rps18 | 18 kDa | Rattus norvegicus |
| PITM1_RAT | Pitpnm1 | 135 kDa | Rattus norvegicus |
| VPS28_RAT | Vps28 | 26 kDa | Rattus norvegicus |
| RETST_RAT | Retsat | 68 kDa | Rattus norvegicus |
| GCR_RAT | Nr3c1 | 88 kDa | Rattus norvegicus |
| NUP93_RAT | Nup93 | 93 kDa | Rattus norvegicus |
| AASD1_RAT | Aarsd1 | 45 kDa | Rattus norvegicus |
| SLIT3_RAT | Slit3 | 168 kDa | Rattus norvegicus |
| CO1A1_RAT | Col1a1 | 138 kDa | Rattus norvegicus |
| GMFG_RAT | Gmfg | 17 kDa | Rattus norvegicus |
| PRG2_RAT | Prg2 | 25 kDa | Rattus norvegicus |
| PERF_RAT | Prf1 | 62 kDa | Rattus norvegicus |
| MPRD_RAT | M6pr | 31 kDa | Rattus norvegicus |
| ADDB_RAT | Add2 | 81 kDa | Rattus norvegicus |
| ACVL1_RAT | Acvrl1 | 57 kDa | Rattus norvegicus |
| RIDA_RAT | Rida | 14 kDa | Rattus norvegicus |
| KSYK_RAT | Syk | 72 kDa | Rattus norvegicus |
| IF6_RAT | Eif6 | 27 kDa | Rattus norvegicus |
| LACB2_RAT | Lactb2 | 32 kDa | Rattus norvegicus |
| SAM50_RAT | Samm50 | 52 kDa | Rattus norvegicus |
| ABCD1_RAT | Abcd1 | 82 kDa | Rattus norvegicus |
| CHSP1_RAT | Carhsp1 | 16 kDa | Rattus norvegicus |

| TANC1_RAT | Tanc1 | 201 kDa | Rattus norvegicus |
| --- | --- | --- | --- |
| ATG3_RAT | Atg3 | 36 kDa | Rattus norvegicus |
| AIFM1_RAT | Aifm1 | 67 kDa | Rattus norvegicus |
| TACD2_RAT | Tacstd2 | 36 kDa | Rattus norvegicus |
| ARY1_RAT | Nat1 | 33 kDa | Rattus norvegicus |
| F151A_RAT | Fam151a | 67 kDa | Rattus norvegicus |
| TGFR2_RAT | Tgfbr2 | 64 kDa | Rattus norvegicus |
| PLF4_RAT | Pf4 | 11 kDa | Rattus norvegicus |
| RWDD1_RAT | Rwdd1 | 28 kDa | Rattus norvegicus |
| S10A1_RAT | S100a1 | 11 kDa | Rattus norvegicus |
| PHAR2_RAT | Phactr2 | 62 kDa | Rattus norvegicus |
| OPTN_RAT | Optn | 67 kDa | Rattus norvegicus |
| MYG_RAT | Mb | 17 kDa | Rattus norvegicus |
| K1C14_RAT | Krt14 | 53 kDa | Rattus norvegicus |
| VISL1_RAT | Vsnl1 | 22 kDa | Rattus norvegicus |
| FAAA_RAT | Fah | 46 kDa | Rattus norvegicus |
| HEM6_RAT | Cpox | 49 kDa | Rattus norvegicus |
| DNJB6_RAT | Dnajb6 | 39 kDa | Rattus norvegicus |
| SCPDL_RAT | Sccpdh | 47 kDa | Rattus norvegicus |
| SSH3_RAT | Ssh3 | 72 kDa | Rattus norvegicus |
| UTER_RAT | Scgb1a1 | 10 kDa | Rattus norvegicus |
| HEXA_RAT | Hexa | 61 kDa | Rattus norvegicus |
| PEX14_RAT | Pex14 | 41 kDa | Rattus norvegicus |
| CMBL_RAT | Cmbl | 28 kDa | Rattus norvegicus |
| TP8L2_RAT | Tnfaip8l2 | 21 kDa | Rattus norvegicus |
| NFIA_RAT | Nfia | 56 kDa | Rattus norvegicus |
| LRRF1_RAT | Lrrfip1 | 80 kDa | Rattus norvegicus |
| STX12_RAT | Stx12 | 31 kDa | Rattus norvegicus |
| RHG27_RAT | Arhgap27 | 97 kDa | Rattus norvegicus |
| ANTR1_RAT | Antxr1 | 62 kDa | Rattus norvegicus |
| RS14_RAT | Rps14 | 16 kDa | Rattus norvegicus |
| MICU1_RAT | Micu1 | 54 kDa | Rattus norvegicus |
| STK3_RAT | Stk3 | 56 kDa | Rattus norvegicus |
| FUCO_RAT | Fuca1 | 53 kDa | Rattus norvegicus |
| RASK_RAT | Kras | 22 kDa | Rattus norvegicus |
| RING1_RAT | Ring1 | 43 kDa | Rattus norvegicus |
| KAT1_RAT | Kyat1 | 52 kDa | Rattus norvegicus |
| IMDH1_RAT | Impdh1 | 55 kDa | Rattus norvegicus |
| UCRI_RAT | Uqcrfs1 | 29 kDa | Rattus norvegicus |
| CGL_RAT | Cth | 44 kDa | Rattus norvegicus |
| TPMT_RAT | Tpmt | 28 kDa | Rattus norvegicus |
| CD44_RAT | Cd44 | 56 kDa | Rattus norvegicus |
| SFTPD_RAT | Sftpd | 38 kDa | Rattus norvegicus |
| OXR1_RAT | Oxr1 | 93 kDa | Rattus norvegicus |
| GPV_RAT | Gp5 | 63 kDa | Rattus norvegicus |
| ARMT1_RAT | Armt1 | 50 kDa | Rattus norvegicus |
| CBLB_RAT | Cblb | 105 kDa | Rattus norvegicus |

| MGST1_RAT | Mgst1 | 17 kDa | Rattus norvegicus |
| --- | --- | --- | --- |
| MARE3_RAT | Mapre3 | 32 kDa | Rattus norvegicus |
| COX1_RAT | Mtco1 | 57 kDa | Rattus norvegicus |
| SNX17_RAT | Snx17 | 53 kDa | Rattus norvegicus |
| CELF1_RAT | Celf1 | 52 kDa | Rattus norvegicus |
| C2D1B_RAT | Cc2d1b | 94 kDa | Rattus norvegicus |
| RECQ1_RAT | Recql | 70 kDa | Rattus norvegicus |
| MYL4_RAT | Myl4 | 21 kDa | Rattus norvegicus |
| CTBP2_RAT | Ctbp2 | 49 kDa | Rattus norvegicus |
| VAMP3_RAT | Vamp3 | 11 kDa | Rattus norvegicus |
| VPS50_RAT | Vps50 | 111 kDa | Rattus norvegicus |
| KIF2A_RAT | Kif2a | 80 kDa | Rattus norvegicus |
| IMPCT_RAT | Impact | 36 kDa | Rattus norvegicus |
| DGLB_RAT | Daglb | 74 kDa | Rattus norvegicus |
| LOX5_RAT | Alox5 | 78 kDa | Rattus norvegicus |
| NUP62_RAT | Nup62 | 53 kDa | Rattus norvegicus |
| STX18_RAT | Stx18 | 39 kDa | Rattus norvegicus |
| DCAF8_RAT | Dcaf8 | 66 kDa | Rattus norvegicus |
| ELP2_RAT | Elp2 | 92 kDa | Rattus norvegicus |
| THOC5_RAT | Thoc5 | 79 kDa | Rattus norvegicus |
| CHMP5_RAT | Chmp5 | 25 kDa | Rattus norvegicus |
| CAMP2_RAT | Camsap2 | 166 kDa | Rattus norvegicus |
| MTND_RAT | Adi1 | 21 kDa | Rattus norvegicus |
| ITM2B_RAT | Itm2b | 30 kDa | Rattus norvegicus |
| RAB3A_RAT | Rab3a | 25 kDa | Rattus norvegicus |
| SAMP_RAT | Apcs | 26 kDa | Rattus norvegicus |
| TPPC3_RAT | Trappc3 | 20 kDa | Rattus norvegicus |
| FYN_RAT | Fyn | 61 kDa | Rattus norvegicus |
| FUT11_RAT | Fut11 | 56 kDa | Rattus norvegicus |
| TDRD7_RAT | Tdrd7 | 125 kDa | Rattus norvegicus |
| EPN1_RAT | Epn1 | 60 kDa | Rattus norvegicus |
| GNB5_RAT | Gnb5 | 39 kDa | Rattus norvegicus |
| ABHGA_RAT | Abhd16a | 63 kDa | Rattus norvegicus |
| NEDD8_RAT | Nedd8 | 9 kDa | Rattus norvegicus |
| KPCD1_RAT | Prkd1 | 102 kDa | Rattus norvegicus |
| PAF1_RAT | Paf1 | 61 kDa | Rattus norvegicus |
| MPPB_RAT | Pmpcb | 54 kDa | Rattus norvegicus |
| PTN9_RAT | Ptpn9 | 68 kDa | Rattus norvegicus |
| PCY2_RAT | Pcyt2 | 45 kDa | Rattus norvegicus |
| CUTA_RAT | Cuta | 19 kDa | Rattus norvegicus |
| PLRG1_RAT | Plrg1 | 57 kDa | Rattus norvegicus |
| DPYD_RAT | Dpyd | 111 kDa | Rattus norvegicus |
| MAVS_RAT | Mavs | 54 kDa | Rattus norvegicus |
| PNPO_RAT | Pnpo | 30 kDa | Rattus norvegicus |
| TP4A2_RAT | Ptp4a2 | 19 kDa | Rattus norvegicus |
| RS25_RAT | Rps25 | 14 kDa | Rattus norvegicus |
| GLNA_RAT | Glul | 42 kDa | Rattus norvegicus |

| SFR1_RAT | Sfr1 | 29 kDa | Rattus norvegicus |
| --- | --- | --- | --- |
| SHOT1_RAT | Shtn1 | 71 kDa | Rattus norvegicus |
| MYO10_RAT | Myo10 | 237 kDa | Rattus norvegicus |
| LTOR3_RAT | Lamtor3 | 14 kDa | Rattus norvegicus |
| MANF_RAT | Manf | 20 kDa | Rattus norvegicus |
| TRXR2_RAT | Txnrd2 | 57 kDa | Rattus norvegicus |
| CASP8_RAT | Casp8 | 55 kDa | Rattus norvegicus |
| SNF8_RAT | Snf8 | 29 kDa | Rattus norvegicus |
| RAB31_RAT | Rab31 | 21 kDa | Rattus norvegicus |
| PACS1_RAT | Pacs1 | 105 kDa | Rattus norvegicus |
| MYG1_RAT | Myg1 | 43 kDa | Rattus norvegicus |
| RL13_RAT | Rpl13 | 24 kDa | Rattus norvegicus |
| MOT2_RAT | Slc16a7 | 53 kDa | Rattus norvegicus |
| SYNPO_RAT | Synpo | 100 kDa | Rattus norvegicus |
| PA1B3_RAT | Pafah1b3 | 26 kDa | Rattus norvegicus |
| DP13B_RAT | Appl2 | 74 kDa | Rattus norvegicus |
| NEK7_RAT | Nek7 | 35 kDa | Rattus norvegicus |
| NAA11_RAT | Naa11 | 28 kDa | Rattus norvegicus |
| JAM1_RAT | F11r | 32 kDa | Rattus norvegicus |
| DRS7B_RAT | Dhrs7b | 35 kDa | Rattus norvegicus |
| ATG5_RAT | Atg5 | 32 kDa | Rattus norvegicus |
| AMRP_RAT | Lrpap1 | 42 kDa | Rattus norvegicus |
| PSPC_RAT | Sftpc | 21 kDa | Rattus norvegicus |
| NEB2_RAT | Ppp1r9b | 90 kDa | Rattus norvegicus |
| SHRM2_RAT | Shroom2 | 158 kDa | Rattus norvegicus |
| S22AI_RAT | Slc22a18 | 43 kDa | Rattus norvegicus |
| MCAT_RAT | Slc25a20 | 33 kDa | Rattus norvegicus |
| NB5R1_RAT | Cyb5r1 | 34 kDa | Rattus norvegicus |
| NICA_RAT | Ncstn | 78 kDa | Rattus norvegicus |
| PRUN1_RAT | Prune1 | 50 kDa | Rattus norvegicus |
| LGMN_RAT | Lgmn | 49 kDa | Rattus norvegicus |
| PEX19_RAT | Pex19 | 32 kDa | Rattus norvegicus |
| CCD91_RAT | Ccdc91 | 50 kDa | Rattus norvegicus |
| CCD51_RAT | Ccdc51 | 46 kDa | Rattus norvegicus |
| ANPRA_RAT | Npr1 | 119 kDa | Rattus norvegicus |
| NASP_RAT | Nasp | 84 kDa | Rattus norvegicus |
| ANPRC_RAT | Npr3 | 60 kDa | Rattus norvegicus |
| LYAG_RAT | Gaa | 106 kDa | Rattus norvegicus |
| OSGEP_RAT | Osgep | 36 kDa | Rattus norvegicus |
| RPAP3_RAT | Rpap3 | 75 kDa | Rattus norvegicus |
| RPGF2_RAT | Rapgef2 | 166 kDa | Rattus norvegicus |
| NU5M_RAT | Mtnd5 | 69 kDa | Rattus norvegicus |
| SAP_RAT | Psap | 61 kDa | Rattus norvegicus |
| ARF4_RAT | Arf4 | 20 kDa | Rattus norvegicus |
| BCAR1_RAT | Bcar1 | 104 kDa | Rattus norvegicus |
| P5I11_RAT | Tp53i11 | 21 kDa | Rattus norvegicus |
| MO4L1_RAT | Morf4l1 | 37 kDa | Rattus norvegicus |

| PPME1_RAT | Ppme1 | 42 kDa | Rattus norvegicus |
| --- | --- | --- | --- |
| NCF1_RAT | Ncf1 | 45 kDa | Rattus norvegicus |
| METK1_RAT | Mat1a | 44 kDa | Rattus norvegicus |
| GLRX3_RAT | Glrx3 | 38 kDa | Rattus norvegicus |
| HYAL2_RAT | Hyal2 | 54 kDa | Rattus norvegicus |
| LIN7C_RAT | Lin7c | 22 kDa | Rattus norvegicus |
| SSRG_RAT | Ssr3 | 21 kDa | Rattus norvegicus |
| DREB_RAT | Dbn1 | 77 kDa | Rattus norvegicus |
| GIPC1_RAT | Gipc1 | 36 kDa | Rattus norvegicus |
| TPD54_RAT | Tpd52l2 | 24 kDa | Rattus norvegicus |
| NET1_RAT | Ntn1 | 68 kDa | Rattus norvegicus |
| PDLI2_RAT | Pdlim2 | 38 kDa | Rattus norvegicus |
| ARFG3_RAT | Arfgap3 | 58 kDa | Rattus norvegicus |
| RAB5A_RAT | Rab5a | 24 kDa | Rattus norvegicus |
| COX5A_RAT | Cox5a | 16 kDa | Rattus norvegicus |
| ATPK_RAT | Atp5mf | 10 kDa | Rattus norvegicus |
| SH24A_RAT | Sh2d4a | 49 kDa | Rattus norvegicus |
| S100B_RAT | S100b | 11 kDa | Rattus norvegicus |
| RL14_RAT | Rpl14 | 23 kDa | Rattus norvegicus |
| SYF1_RAT | Xab2 | 100 kDa | Rattus norvegicus |
| LIPL_RAT | Lpl | 53 kDa | Rattus norvegicus |
| VKORL_RAT | Vkorc1l1 | 20 kDa | Rattus norvegicus |
| NUBP1_RAT | Nubp1 | 34 kDa | Rattus norvegicus |
| GULP1_RAT | Gulp1 | 34 kDa | Rattus norvegicus |
| SYNJ1_RAT | Synj1 | 173 kDa | Rattus norvegicus |
| MAP6_RAT | Map6 | 100 kDa | Rattus norvegicus |
| OTU1_RAT | Yod1 | 37 kDa | Rattus norvegicus |
| RAP2B_RAT | Rap2b | 21 kDa | Rattus norvegicus |
| THIOM_RAT | Txn2 | 18 kDa | Rattus norvegicus |
| RAE1L_RAT | Rae1 | 41 kDa | Rattus norvegicus |
| HSP72_RAT | Hspa2 | 70 kDa | Rattus norvegicus |
| DHX36_RAT | Dhx36 | 114 kDa | Rattus norvegicus |
| FAF2_RAT | Faf2 | 41 kDa | Rattus norvegicus |
| SL9A1_RAT | Slc9a1 | 92 kDa | Rattus norvegicus |
| HOME3_RAT | Homer3 | 40 kDa | Rattus norvegicus |
| H2A1_RAT |  | 14 kDa | Rattus norvegicus |
| MMP2_RAT | Mmp2 | 74 kDa | Rattus norvegicus |
| PLCD_RAT | Agpat4 | 44 kDa | Rattus norvegicus |
| NDUA5_RAT | Ndufa5 | 13 kDa | Rattus norvegicus |
| ILKAP_RAT | Ilkap | 43 kDa | Rattus norvegicus |
| UBXN1_RAT | Ubxn1 | 34 kDa | Rattus norvegicus |
| NEO1_RAT | Neo1 | 151 kDa | Rattus norvegicus |
| CD59_RAT | Cd59 | 14 kDa | Rattus norvegicus |
| SODC_RAT | Sod1 | 16 kDa | Rattus norvegicus |
| RS21_RAT | Rps21 | 9 kDa | Rattus norvegicus |
| OGFR_RAT | Ogfr | 65 kDa | Rattus norvegicus |
| MXRA8_RAT | Mxra8 | 43 kDa | Rattus norvegicus |

| ACY2_RAT | Aspa | 35 kDa | Rattus norvegicus |
| --- | --- | --- | --- |
| NUP88_RAT | Nup88 | 84 kDa | Rattus norvegicus |
| MYPC_RAT | Mybpc3 | 141 kDa | Rattus norvegicus |
| RAB43_RAT | Rab43 | 23 kDa | Rattus norvegicus |
| CHP1_RAT | Chp1 | 22 kDa | Rattus norvegicus |
| SHLB2_RAT | Sh3glb2 | 45 kDa | Rattus norvegicus |
| ICLN_RAT | Clns1a | 26 kDa | Rattus norvegicus |
| CNDP1_RAT | Cndp1 | 55 kDa | Rattus norvegicus |
| KCRS_RAT | Ckmt2 | 47 kDa | Rattus norvegicus |
| G6PC3_RAT | G6pc3 | 39 kDa | Rattus norvegicus |
| DIAC_RAT | Ctbs | 42 kDa | Rattus norvegicus |
| TBA1C_RAT | Tuba1c | 50 kDa | Rattus norvegicus |
| S61A1_RAT | Sec61a1 | 52 kDa | Rattus norvegicus |
| DJC10_RAT | Dnajc10 | 91 kDa | Rattus norvegicus |
| MCES_RAT | Rnmt | 53 kDa | Rattus norvegicus |
| CNO10_RAT | Cnot10 | 82 kDa | Rattus norvegicus |
| IST1_RAT | Ist1 | 40 kDa | Rattus norvegicus |
| CNTN1_RAT | Cntn1 | 113 kDa | Rattus norvegicus |
| MSMO1_RAT | Msmo1 | 35 kDa | Rattus norvegicus |
| GORS2_RAT | Gorasp2 | 47 kDa | Rattus norvegicus |
| S12A4_RAT | Slc12a4 | 121 kDa | Rattus norvegicus |
| RHAG_RAT | Rhag | 49 kDa | Rattus norvegicus |
| PPAL_RAT | Acp2 | 48 kDa | Rattus norvegicus |
| EMC8_RAT | Emc8 | 23 kDa | Rattus norvegicus |
| AL1L1_RAT | Aldh1l1 | 99 kDa | Rattus norvegicus |
| TBCEL_RAT | Tbcel | 48 kDa | Rattus norvegicus |
| SRC8_RAT | Cttn | 57 kDa | Rattus norvegicus |
| UBP48_RAT | Usp48 | 119 kDa | Rattus norvegicus |
| RS6_RAT | Rps6 | 29 kDa | Rattus norvegicus |
| DPEP2_RAT | Dpep2 | 53 kDa | Rattus norvegicus |
| MA1B1_RAT | Man1b1 | 75 kDa | Rattus norvegicus |
| VAV_RAT | Vav1 | 98 kDa | Rattus norvegicus |
| PHF5A_RAT | Phf5a | 12 kDa | Rattus norvegicus |
| SAR1B_RAT | Sar1b | 22 kDa | Rattus norvegicus |
| PUR1_RAT | Ppat | 57 kDa | Rattus norvegicus |
| DNJC8_RAT | Dnajc8 | 30 kDa | Rattus norvegicus |
| NSMA_RAT | Smpd2 | 48 kDa | Rattus norvegicus |
| GRIA4_RAT | Gria4 | 101 kDa | Rattus norvegicus |
| PP4C_RAT | Ppp4c | 35 kDa | Rattus norvegicus |
| TOM34_RAT | Tomm34 | 34 kDa | Rattus norvegicus |
| HBS1L_RAT | Hbs1l | 75 kDa | Rattus norvegicus |
| GLPC_RAT | Gypc | 10 kDa | Rattus norvegicus |
| DCMC_RAT | Mlycd | 55 kDa | Rattus norvegicus |
| NAKD2_RAT | Nadk2 | 48 kDa | Rattus norvegicus |
| PP1RB_RAT | Ppp1r11 | 14 kDa | Rattus norvegicus |
| PP1G_RAT | Ppp1cc | 37 kDa | Rattus norvegicus |
| HB2B_RAT | RT1-Bb | 30 kDa | Rattus norvegicus |

| FBX7_RAT | Fbxo7 | 58 kDa | Rattus norvegicus |
| --- | --- | --- | --- |
| FMO5_RAT | Fmo5 | 60 kDa | Rattus norvegicus |
| MCPT2_RAT | Mcpt2 | 27 kDa | Rattus norvegicus |
| TM168_RAT | Tmem168 | 80 kDa | Rattus norvegicus |
| RGS7_RAT | Rgs7 | 56 kDa | Rattus norvegicus |
| PBLD_RAT | Pbld | 32 kDa | Rattus norvegicus |
| ORN_RAT | Rexo2 | 27 kDa | Rattus norvegicus |
| LAMP2_RAT | Lamp2 | 45 kDa | Rattus norvegicus |
| KPRB_RAT | Prpsap2 | 41 kDa | Rattus norvegicus |
| BI2L1_RAT | Baiap2l1 | 57 kDa | Rattus norvegicus |
| YKT6_RAT | Ykt6 | 22 kDa | Rattus norvegicus |
| RAB12_RAT | Rab12 | 27 kDa | Rattus norvegicus |
| COX41_RAT | Cox4i1 | 20 kDa | Rattus norvegicus |
| ANKL2_RAT | Ankle2 | 106 kDa | Rattus norvegicus |
| WDR44_RAT | Wdr44 | 101 kDa | Rattus norvegicus |
| CASQ2_RAT | Casq2 | 48 kDa | Rattus norvegicus |
| ZW10_RAT | Zw10 | 88 kDa | Rattus norvegicus |
| KAT3_RAT | Kyat3 | 51 kDa | Rattus norvegicus |
| ATOX1_RAT | Atox1 | 7 kDa | Rattus norvegicus |
| FLVC2_RAT | Flvcr2 | 60 kDa | Rattus norvegicus |
| PLD3_RAT | Pld3 | 54 kDa | Rattus norvegicus |
| TPPC1_RAT | Trappc1 | 17 kDa | Rattus norvegicus |
| ZRAB2_RAT | Zranb2 | 37 kDa | Rattus norvegicus |
| MASP2_RAT | Masp2 | 76 kDa | Rattus norvegicus |
| OCAD1_RAT | Ociad1 | 28 kDa | Rattus norvegicus |
| TPPC2_RAT | Trappc2 | 16 kDa | Rattus norvegicus |
| CH3L1_RAT | Chi3l1 | 42 kDa | Rattus norvegicus |
| DAPK3_RAT | Dapk3 | 51 kDa | Rattus norvegicus |
| STEA4_RAT | Steap4 | 53 kDa | Rattus norvegicus |
| LICH_RAT | Lipa | 45 kDa | Rattus norvegicus |
| SSDH_RAT | Aldh5a1 | 56 kDa | Rattus norvegicus |
| KLC4_RAT | Klc4 | 69 kDa | Rattus norvegicus |
| HSPB6_RAT | Hspb6 | 18 kDa | Rattus norvegicus |
| FMO2_RAT | Fmo2 | 61 kDa | Rattus norvegicus |
| CPSM_RAT | Cps1 | 165 kDa | Rattus norvegicus |
| PP14A_RAT | Ppp1r14a | 17 kDa | Rattus norvegicus |
| SEP15_RAT | Selenof | 18 kDa | Rattus norvegicus |
| COMD5_RAT | Commd5 | 24 kDa | Rattus norvegicus |
| KPCL_RAT | Prkch | 78 kDa | Rattus norvegicus |
| RT07_RAT | Mrps7 | 28 kDa | Rattus norvegicus |
| EF2K_RAT | Eef2k | 81 kDa | Rattus norvegicus |
| EAA3_RAT | Slc1a1 | 57 kDa | Rattus norvegicus |
| GLO2_RAT | Hagh | 34 kDa | Rattus norvegicus |
| SNP29_RAT | Snap29 | 29 kDa | Rattus norvegicus |
| RBM10_RAT | Rbm10 | 94 kDa | Rattus norvegicus |
| PLPP3_RAT | Plpp3 | 35 kDa | Rattus norvegicus |
| IF1A_RAT | Eif1a | 17 kDa | Rattus norvegicus |

| PDE12_RAT | Pde12 | 67 kDa | Rattus norvegicus |
| --- | --- | --- | --- |
| DUS3L_RAT | Dus3l | 72 kDa | Rattus norvegicus |
| PSMD9_RAT | Psmd9 | 25 kDa | Rattus norvegicus |
| CHMP3_RAT | Chmp3 | 25 kDa | Rattus norvegicus |
| ITB6_RAT | Itgb6 | 86 kDa | Rattus norvegicus |
| EFGM_RAT | Gfm1 | 83 kDa | Rattus norvegicus |
| NUP85_RAT | Nup85 | 75 kDa | Rattus norvegicus |
| DNJC5_RAT | Dnajc5 | 22 kDa | Rattus norvegicus |
| WDR7_RAT | Wdr7 | 163 kDa | Rattus norvegicus |
| DYN1_RAT | Dnm1 | 97 kDa | Rattus norvegicus |
| NU1M_RAT | Mtnd1 | 36 kDa | Rattus norvegicus |
| TACO1_RAT | Taco1 | 33 kDa | Rattus norvegicus |
| ARM10_RAT | Armc10 | 33 kDa | Rattus norvegicus |
| GGT5_RAT | Ggt5 | 62 kDa | Rattus norvegicus |
| TBCE_RAT | Tbce | 59 kDa | Rattus norvegicus |
| KCAB2_RAT | Kcnab2 | 41 kDa | Rattus norvegicus |
| PDCL3_RAT | Pdcl3 | 28 kDa | Rattus norvegicus |
| ACTC_RAT | Actc1 | 42 kDa | Rattus norvegicus |
| ATP7A_RAT | Atp7a | 162 kDa | Rattus norvegicus |
| RN114_RAT | Rnf114 | 26 kDa | Rattus norvegicus |
| TMED3_RAT | Tmed3 | 26 kDa | Rattus norvegicus |
| LIFR_RAT | Lifr | 122 kDa | Rattus norvegicus |
| SPG21_RAT | Spg21 | 30 kDa | Rattus norvegicus |
| BAX_RAT | Bax | 21 kDa | Rattus norvegicus |
| CC50A_RAT | Tmem30a | 37 kDa | Rattus norvegicus |
| ADNP_RAT | Adnp | 124 kDa | Rattus norvegicus |
| SGTA_RAT | Sgta | 34 kDa | Rattus norvegicus |
| LZIC_RAT | Lzic | 21 kDa | Rattus norvegicus |
| ENOG_RAT | Eno2 | 47 kDa | Rattus norvegicus |
| RL38_RAT | Rpl38 | 8 kDa | Rattus norvegicus |
| STABP_RAT | Stambp | 49 kDa | Rattus norvegicus |
| ARRB2_RAT | Arrb2 | 46 kDa | Rattus norvegicus |
| UBE2Z_RAT | Ube2z | 38 kDa | Rattus norvegicus |
| PK3CA_RAT | Pik3ca | 124 kDa | Rattus norvegicus |
| FGD4_RAT | Fgd4 | 86 kDa | Rattus norvegicus |
| MMP8_RAT | Mmp8 | 53 kDa | Rattus norvegicus |
| ACE2_RAT | Ace2 | 92 kDa | Rattus norvegicus |
| GEPH_RAT | Gphn | 83 kDa | Rattus norvegicus |
| MAAI_RAT | Gstz1 | 24 kDa | Rattus norvegicus |
| CZIB_RAT | Czib | 18 kDa | Rattus norvegicus |
| AGFG1_RAT | Agfg1 | 58 kDa | Rattus norvegicus |
| CAP2_RAT | Cap2 | 53 kDa | Rattus norvegicus |
| GLPK_RAT | Gk | 57 kDa | Rattus norvegicus |
| SRSF5_RAT | Srsf5 | 31 kDa | Rattus norvegicus |
| RL36_RAT | Rpl36 | 12 kDa | Rattus norvegicus |
| BAAT_RAT | Baat | 46 kDa | Rattus norvegicus |
| KPYR_RAT | Pklr | 62 kDa | Rattus norvegicus |

| BACH_RAT | Acot7 | 43 kDa | Rattus norvegicus |
| --- | --- | --- | --- |
| CPIN1_RAT | Ciapin1 | 33 kDa | Rattus norvegicus |
| PTSS1_RAT | Ptdss1 | 56 kDa | Rattus norvegicus |
| MP2K2_RAT | Map2k2 | 44 kDa | Rattus norvegicus |
| UGDH_RAT | Ugdh | 55 kDa | Rattus norvegicus |
| SH3G2_RAT | Sh3gl2 | 40 kDa | Rattus norvegicus |
| TCEA1_RAT | Tcea1 | 34 kDa | Rattus norvegicus |
| TCP4_RAT | Sub1 | 14 kDa | Rattus norvegicus |
| MED4_RAT | Med4 | 30 kDa | Rattus norvegicus |
| UBP46_RAT | Usp46 | 42 kDa | Rattus norvegicus |
| RAB4B_RAT | Rab4b | 24 kDa | Rattus norvegicus |
| MPC2_RAT | Mpc2 | 14 kDa | Rattus norvegicus |
| ISPD_RAT | Crppa | 49 kDa | Rattus norvegicus |
| BHMT1_RAT | Bhmt | 45 kDa | Rattus norvegicus |
| AUHM_RAT | Auh | 33 kDa | Rattus norvegicus |
| CPSF4_RAT | Cpsf4 | 27 kDa | Rattus norvegicus |
| SHPS1_RAT | Sirpa | 56 kDa | Rattus norvegicus |
| ECM1_RAT | Ecm1 | 63 kDa | Rattus norvegicus |
| LTBP2_RAT | Ltbp2 | 190 kDa | Rattus norvegicus |
| PRPS1_RAT | Prps1 | 35 kDa | Rattus norvegicus |
| MI4GD_RAT | Mif4gd | 25 kDa | Rattus norvegicus |
| FKBP8_RAT | Fkbp8 | 44 kDa | Rattus norvegicus |
| RALA_RAT | Rala | 24 kDa | Rattus norvegicus |
| RAB9A_RAT | Rab9a | 23 kDa | Rattus norvegicus |
| DEGS1_RAT | Degs1 | 38 kDa | Rattus norvegicus |
| RIPL1_RAT | Rilpl1 | 47 kDa | Rattus norvegicus |
| UBP10_RAT | Usp10 | 87 kDa | Rattus norvegicus |
| PGTA_RAT | Rabggta | 65 kDa | Rattus norvegicus |
| PDC10_RAT | Pdcd10 | 24 kDa | Rattus norvegicus |
| UFSP2_RAT | Ufsp2 | 52 kDa | Rattus norvegicus |
| FABPH_RAT | Fabp3 | 15 kDa | Rattus norvegicus |
| RAB3D_RAT | Rab3d | 24 kDa | Rattus norvegicus |
| NQO2_RAT | Nqo2 | 26 kDa | Rattus norvegicus |
| DNS2A_RAT | Dnase2 | 38 kDa | Rattus norvegicus |
| RCN3_RAT | Rcn3 | 38 kDa | Rattus norvegicus |
| MANBA_RAT | Manba | 101 kDa | Rattus norvegicus |
| OGRL1_RAT | Ogfrl1 | 53 kDa | Rattus norvegicus |
| NUBP2_RAT | Nubp2 | 29 kDa | Rattus norvegicus |
| GRP2_RAT | Rasgrp2 | 69 kDa | Rattus norvegicus |
| TM9S4_RAT | Tm9sf4 | 75 kDa | Rattus norvegicus |
| RGS10_RAT | Rgs10 | 21 kDa | Rattus norvegicus |
| USBP1_RAT | Ushbp1 | 75 kDa | Rattus norvegicus |
| ACD11_RAT | Acad11 | 87 kDa | Rattus norvegicus |
| REEP5_RAT | Reep5 | 21 kDa | Rattus norvegicus |
| SPRL1_RAT | Sparcl1 | 71 kDa | Rattus norvegicus |
| GPN3_RAT | Gpn3 | 33 kDa | Rattus norvegicus |
| EFTS_RAT | Tsfm | 35 kDa | Rattus norvegicus |

| SMAD4_RAT | Smad4 | 60 kDa | Rattus norvegicus |
| --- | --- | --- | --- |
| NTM1A_RAT | Ntmt1 | 25 kDa | Rattus norvegicus |
| ARH_RAT | Ldlrap1 | 34 kDa | Rattus norvegicus |
| CIB1_RAT | Cib1 | 22 kDa | Rattus norvegicus |
| ENTP5_RAT | Entpd5 | 47 kDa | Rattus norvegicus |
| NXF1_RAT | Nxf1 | 70 kDa | Rattus norvegicus |
| AP3M1_RAT | Ap3m1 | 47 kDa | Rattus norvegicus |
| NECP1_RAT | Necap1 | 30 kDa | Rattus norvegicus |
| MOB1A_RAT | Mob1a | 25 kDa | Rattus norvegicus |
| HYES_RAT | Ephx2 | 62 kDa | Rattus norvegicus |
| RL19_RAT | Rpl19 | 23 kDa | Rattus norvegicus |
| PHS_RAT | Pcbd1 | 12 kDa | Rattus norvegicus |
| RS23_RAT | Rps23 | 16 kDa | Rattus norvegicus |
| CAVN3_RAT | Cavin3 | 28 kDa | Rattus norvegicus |
| SIAE_RAT | Siae | 60 kDa | Rattus norvegicus |
| PDK2_RAT | Pdk2 | 46 kDa | Rattus norvegicus |
| RF1ML_RAT | Mtrf1l | 42 kDa | Rattus norvegicus |
| CFA20_RAT | Cfap20 | 19 kDa | Rattus norvegicus |
| EMD_RAT | Emd | 30 kDa | Rattus norvegicus |
| LHPP_RAT | Lhpp | 29 kDa | Rattus norvegicus |
| VTI1B_RAT | Vti1b | 27 kDa | Rattus norvegicus |
| AG10B_RAT | Alg10b | 56 kDa | Rattus norvegicus |
| CHKB_RAT | Chkb | 45 kDa | Rattus norvegicus |
| CACO1_RAT | Calcoco1 | 77 kDa | Rattus norvegicus |
| C1S_RAT | C1s | 77 kDa | Rattus norvegicus |
| CTCF_RAT | Ctcf | 84 kDa | Rattus norvegicus |
| STX8_RAT | Stx8 | 27 kDa | Rattus norvegicus |
| NOL3_RAT | Nol3 | 25 kDa | Rattus norvegicus |
| PP2BB_RAT | Ppp3cb | 59 kDa | Rattus norvegicus |
| ATAD1_RAT | Atad1 | 41 kDa | Rattus norvegicus |
| NGAL_RAT | Lcn2 | 22 kDa | Rattus norvegicus |
| TMX2_RAT | Tmx2 | 34 kDa | Rattus norvegicus |
| STX3_RAT | Stx3 | 33 kDa | Rattus norvegicus |
| SEPT5_RAT | 5-Sep | 43 kDa | Rattus norvegicus |
| DYLT1_RAT | Dynlt1 | 12 kDa | Rattus norvegicus |
| EMC2_RAT | Emc2 | 35 kDa | Rattus norvegicus |
| CYB5B_RAT | Cyb5b | 16 kDa | Rattus norvegicus |
| SEP10_RAT | 10-Sep | 53 kDa | Rattus norvegicus |
| K2C80_RAT | Krt80 | 51 kDa | Rattus norvegicus |
| CRYM_RAT | Crym | 34 kDa | Rattus norvegicus |
| CSRP3_RAT | Csrp3 | 21 kDa | Rattus norvegicus |
| RL24_RAT | Rpl24 | 18 kDa | Rattus norvegicus |
| SIL1_RAT | Sil1 | 52 kDa | Rattus norvegicus |
| UFD1_RAT | Ufd1 | 34 kDa | Rattus norvegicus |
| OLFL3_RAT | Olfml3 | 46 kDa | Rattus norvegicus |
| NCAM1_RAT | Ncam1 | 95 kDa | Rattus norvegicus |
| LZTL1_RAT | Lztfl1 | 35 kDa | Rattus norvegicus |

| PPIL3_RAT | Ppil3 | 18 kDa | Rattus norvegicus |
| --- | --- | --- | --- |
| TBL3_RAT | Tbl3 | 88 kDa | Rattus norvegicus |
| GBA2_RAT | Gba2 | 103 kDa | Rattus norvegicus |
| RL23A_RAT | Rpl23a | 18 kDa | Rattus norvegicus |
| APOM_RAT | Apom | 22 kDa | Rattus norvegicus |
| PLD2_RAT | Pld2 | 106 kDa | Rattus norvegicus |
| RED_RAT | Ik | 66 kDa | Rattus norvegicus |
| PI51A_RAT | Pip5k1a | 61 kDa | Rattus norvegicus |
| F16P1_RAT | Fbp1 | 40 kDa | Rattus norvegicus |
| GOLP3_RAT | Golph3 | 34 kDa | Rattus norvegicus |
| IL1AP_RAT | Il1rap | 66 kDa | Rattus norvegicus |
| TBCA_RAT | Tbca | 13 kDa | Rattus norvegicus |
| VGFR1_RAT | Flt1 | 150 kDa | Rattus norvegicus |
| IL18_RAT | Il18 | 22 kDa | Rattus norvegicus |
| MBPHL_RAT | Mybphl | 39 kDa | Rattus norvegicus |
| CREL2_RAT | Creld2 | 38 kDa | Rattus norvegicus |
| MARC2_RAT | 2-Mar | 38 kDa | Rattus norvegicus |
| SELS_RAT | Selenos | 21 kDa | Rattus norvegicus |
| AAGAB_RAT | Aagab | 34 kDa | Rattus norvegicus |
| STX7_RAT | Stx7 | 30 kDa | Rattus norvegicus |
| EI2BG_RAT | Eif2b3 | 50 kDa | Rattus norvegicus |
| SETD3_RAT | Setd3 | 67 kDa | Rattus norvegicus |
| RHEB_RAT | Rheb | 20 kDa | Rattus norvegicus |
| GNAI1_RAT | Gnai1 | 40 kDa | Rattus norvegicus |
| U2AF4_RAT | U2af1l4 | 26 kDa | Rattus norvegicus |
| CAH8_RAT | Ca8 | 33 kDa | Rattus norvegicus |
| EI2BB_RAT | Eif2b2 | 39 kDa | Rattus norvegicus |
| CML1_RAT | Cmklr1 | 42 kDa | Rattus norvegicus |
| BL1S6_RAT | Bloc1s6 | 20 kDa | Rattus norvegicus |
| P4K2A_RAT | Pi4k2a | 54 kDa | Rattus norvegicus |
| MGAT1_RAT | Mgat1 | 52 kDa | Rattus norvegicus |
| NIF3L_RAT | Nif3l1 | 42 kDa | Rattus norvegicus |
| QCR6_RAT | Uqcrh | 10 kDa | Rattus norvegicus |
| DUT_RAT | Dut | 22 kDa | Rattus norvegicus |
| PHYD1_RAT | Phyhd1 | 33 kDa | Rattus norvegicus |
| PLPP_RAT | Pdxp | 33 kDa | Rattus norvegicus |
| ACBP_RAT | Dbi | 10 kDa | Rattus norvegicus |
| RL13A_RAT | Rpl13a | 23 kDa | Rattus norvegicus |
| RPB7_RAT | Polr2g | 19 kDa | Rattus norvegicus |
| BUD31_RAT | Bud31 | 17 kDa | Rattus norvegicus |
| ARSB_RAT | Arsb | 59 kDa | Rattus norvegicus |
| EFNB1_RAT | Efnb1 | 38 kDa | Rattus norvegicus |
| RUS1_RAT |  | 51 kDa | Rattus norvegicus |
| TPC2L_RAT | Trappc2l | 16 kDa | Rattus norvegicus |
| FBX6_RAT | Fbxo6 | 33 kDa | Rattus norvegicus |
| LRC8C_RAT | Lrrc8c | 92 kDa | Rattus norvegicus |
| PLD1_RAT | Pld1 | 124 kDa | Rattus norvegicus |

| CASP3_RAT | Casp3 | 31 kDa | Rattus norvegicus |
| --- | --- | --- | --- |
| DNJC2_RAT | Dnajc2 | 72 kDa | Rattus norvegicus |
| KC1G3_RAT | Csnk1g3 | 51 kDa | Rattus norvegicus |
| RAB6A_RAT | Rab6a | 24 kDa | Rattus norvegicus |
| MB12A_RAT | Mvb12a | 29 kDa | Rattus norvegicus |
| IGHD_RAT |  | 22 kDa | Rattus norvegicus |
| PH11L_RAT | Phf11l | 33 kDa | Rattus norvegicus |
| ADRB2_RAT | Adrb2 | 47 kDa | Rattus norvegicus |
| HB2D_RAT | RT1-Db1 | 30 kDa | Rattus norvegicus |
| DX39A_RAT | Ddx39a | 49 kDa | Rattus norvegicus |
| SEPP1_RAT | Selenop | 43 kDa | Rattus norvegicus |
| COG6_RAT | Cog6 | 73 kDa | Rattus norvegicus |
| ENPP6_RAT | Enpp6 | 51 kDa | Rattus norvegicus |
| PP4R1_RAT | Ppp4r1 | 106 kDa | Rattus norvegicus |
| NXPE4_RAT | Nxpe4 | 62 kDa | Rattus norvegicus |
| PDLI3_RAT | Pdlim3 | 39 kDa | Rattus norvegicus |
| ARFG1_RAT | Arfgap1 | 45 kDa | Rattus norvegicus |
| SCRN1_RAT | Scrn1 | 46 kDa | Rattus norvegicus |
| P2RX7_RAT | P2rx7 | 68 kDa | Rattus norvegicus |
| PHF11_RAT | Phf11 | 37 kDa | Rattus norvegicus |
| NDRG3_RAT | Ndrg3 | 42 kDa | Rattus norvegicus |
| ILF2_RAT | Ilf2 | 51 kDa | Rattus norvegicus |
| B2MG_RAT | B2m | 14 kDa | Rattus norvegicus |
| THAS_RAT | Tbxas1 | 60 kDa | Rattus norvegicus |
| TOIP2_RAT | Tor1aip2 | 62 kDa | Rattus norvegicus |
| TM100_RAT | Tmem100 | 14 kDa | Rattus norvegicus |
| KPCD2_RAT | Prkd2 | 96 kDa | Rattus norvegicus |
| EIF3J_RAT | Eif3j | 29 kDa | Rattus norvegicus |
| RL37P_RAT | Rpl37a-ps1 | 10 kDa | Rattus norvegicus |
| RASF2_RAT | Rassf2 | 38 kDa | Rattus norvegicus |
| NFS1_RAT | Nfs1 | 50 kDa | Rattus norvegicus |
| ATG9A_RAT | Atg9a | 94 kDa | Rattus norvegicus |
| DCXR_RAT | Dcxr | 26 kDa | Rattus norvegicus |
| NADE_RAT | Nadsyn1 | 82 kDa | Rattus norvegicus |
| AK1D1_RAT | Akr1d1 | 37 kDa | Rattus norvegicus |
| RBP1_RAT | Ralbp1 | 75 kDa | Rattus norvegicus |
| MPPA_RAT | Pmpca | 59 kDa | Rattus norvegicus |
| FADS3_RAT | Fads3 | 51 kDa | Rattus norvegicus |
| GSTK1_RAT | Gstk1 | 25 kDa | Rattus norvegicus |
| CP2BC_RAT | Cyp2b12 | 56 kDa | Rattus norvegicus |
| TPRGL_RAT | Tprg1l | 30 kDa | Rattus norvegicus |
| MCTS1_RAT | Mcts1 | 21 kDa | Rattus norvegicus |
| AAKB2_RAT | Prkab2 | 30 kDa | Rattus norvegicus |
| TSPO_RAT | Tspo | 19 kDa | Rattus norvegicus |
| ARAF_RAT | Araf | 68 kDa | Rattus norvegicus |
| TIP_RAT | Itfg1 | 67 kDa | Rattus norvegicus |
| PGRP1_RAT | Pglyrp1 | 21 kDa | Rattus norvegicus |

| CY24A_RAT | Cyba | 21 kDa | Rattus norvegicus |
| --- | --- | --- | --- |
| VAMP8_RAT | Vamp8 | 11 kDa | Rattus norvegicus |
| SIGIR_RAT | Sigirr | 46 kDa | Rattus norvegicus |
| KC1D_RAT | Csnk1d | 47 kDa | Rattus norvegicus |
| CK068_RAT | Bles03 | 31 kDa | Rattus norvegicus |
| ENPP5_RAT | Enpp5 | 54 kDa | Rattus norvegicus |
| MICA1_RAT | Mical1 | 117 kDa | Rattus norvegicus |
| 5NT3B_RAT | Nt5c3b | 35 kDa | Rattus norvegicus |
| SHIP2_RAT | Inppl1 | 139 kDa | Rattus norvegicus |
| STX6_RAT | Stx6 | 29 kDa | Rattus norvegicus |
| BTD_RAT | Btd | 58 kDa | Rattus norvegicus |
| FHIT_RAT | Fhit | 17 kDa | Rattus norvegicus |
| FGR_RAT | Fgr | 59 kDa | Rattus norvegicus |
| HMOX1_RAT | Hmox1 | 33 kDa | Rattus norvegicus |
| ZC3HE_RAT | Zc3h14 | 83 kDa | Rattus norvegicus |
| CDK4_RAT | Cdk4 | 34 kDa | Rattus norvegicus |
| FA10_RAT | F10 | 54 kDa | Rattus norvegicus |
| KC1G1_RAT | Csnk1g1 | 45 kDa | Rattus norvegicus |
| PFD2_RAT | Pfdn2 | 17 kDa | Rattus norvegicus |
| IF2H_RAT | Eif2s3y | 51 kDa | Rattus norvegicus |
| HSBP1_RAT | Hsbp1 | 9 kDa | Rattus norvegicus |
| HDHD2_RAT | Hdhd2 | 29 kDa | Rattus norvegicus |
| LRC8A_RAT | Lrrc8a | 94 kDa | Rattus norvegicus |
| SPRY7_RAT | Spryd7 | 22 kDa | Rattus norvegicus |
| HMGB2_RAT | Hmgb2 | 24 kDa | Rattus norvegicus |
| RHOB_RAT | Rhob | 22 kDa | Rattus norvegicus |
| C1QA_RAT | C1qa | 26 kDa | Rattus norvegicus |
| RS27L_RAT | Rps27l | 9 kDa | Rattus norvegicus |
| CD37L_RAT | Cdc37l1 | 38 kDa | Rattus norvegicus |
| ALR_RAT | Gfer | 23 kDa | Rattus norvegicus |
| ELOB_RAT | Elob | 13 kDa | Rattus norvegicus |
| RBM45_RAT | Rbm45 | 53 kDa | Rattus norvegicus |
| LBP_RAT | Lbp | 54 kDa | Rattus norvegicus |
| CAH5B_RAT | Ca5b | 37 kDa | Rattus norvegicus |
| RAB4A_RAT | Rab4a | 24 kDa | Rattus norvegicus |
| UPK1B_RAT | Upk1b | 30 kDa | Rattus norvegicus |
| P2RX1_RAT | P2rx1 | 45 kDa | Rattus norvegicus |
| MYP0_RAT | Mpz | 28 kDa | Rattus norvegicus |
| FKBP9_RAT | Fkbp9 | 63 kDa | Rattus norvegicus |
| PCAT2_RAT | Lpcat2 | 60 kDa | Rattus norvegicus |
| PREB_RAT | Preb | 45 kDa | Rattus norvegicus |
| CNTFR_RAT | Cntfr | 41 kDa | Rattus norvegicus |
| HTRA1_RAT | Htra1 | 51 kDa | Rattus norvegicus |
| NDUS4_RAT | Ndufs4 | 20 kDa | Rattus norvegicus |
| ELMO3_RAT | Elmo3 | 82 kDa | Rattus norvegicus |
| GAMT_RAT | Gamt | 26 kDa | Rattus norvegicus |
| CO1A2_RAT | Col1a2 | 130 kDa | Rattus norvegicus |

| BET1_RAT | Bet1 | 13 kDa | Rattus norvegicus |
| --- | --- | --- | --- |
| A1AG_RAT | Orm1 | 24 kDa | Rattus norvegicus |
| XYLB_RAT | Xylb | 58 kDa | Rattus norvegicus |
| C4BPB_RAT | C4bpb | 29 kDa | Rattus norvegicus |
| CLYBL_RAT | Clybl | 37 kDa | Rattus norvegicus |
| HSP7E_RAT | Hspa14 | 54 kDa | Rattus norvegicus |
| PP14B_RAT | Ppp1r14b | 16 kDa | Rattus norvegicus |
| M1IP1_RAT | Mid1ip1 | 20 kDa | Rattus norvegicus |
| SPTC1_RAT | Sptlc1 | 53 kDa | Rattus norvegicus |
| UBL4A_RAT | Ubl4a | 18 kDa | Rattus norvegicus |
| PNCB_RAT | Naprt | 59 kDa | Rattus norvegicus |
| PGAM2_RAT | Pgam2 | 29 kDa | Rattus norvegicus |
| TCO2_RAT | Tcn2 | 47 kDa | Rattus norvegicus |
| PPA5_RAT | Acp5 | 37 kDa | Rattus norvegicus |
| FCN2_RAT | Fcn2 | 35 kDa | Rattus norvegicus |
| CH082_RAT |  | 24 kDa | Rattus norvegicus |
| HPPD_RAT | Hpd | 45 kDa | Rattus norvegicus |
| PGDH_RAT | Hpgd | 29 kDa | Rattus norvegicus |
| ITM2C_RAT | Itm2c | 30 kDa | Rattus norvegicus |
| ARGI1_RAT | Arg1 | 35 kDa | Rattus norvegicus |
| ICMT_RAT | Icmt | 27 kDa | Rattus norvegicus |
| HMCS1_RAT | Hmgcs1 | 57 kDa | Rattus norvegicus |
| FABPL_RAT | Fabp1 | 14 kDa | Rattus norvegicus |
|  |  |  |  |

**Table 2: Methylation of histone 3.1**

| **Current tab: PTM Spectrum Counts** | | | | | | | | | | | | | | | | | | | |
| --- | --- | --- | --- | --- | --- | --- | --- | --- | --- | --- | --- | --- | --- | --- | --- | --- | --- | --- | --- |
| **Current protein: (H31_RAT) Histone H3.1 OS=Rattus norvegicus OX=10116 PE=1 SV=3** | | | | | | | | | | | | | | | | | | | |
| **Site** | Modification | Best Ascore | Localization Probability | 133M | 148M | 23M | 37M | 123M | 124M | 131M | 132M | SD1 | SD2 | SD3 | SD4 | 147M | 149M | 150M | 151M |
| **K28** | Dimethyl | 1,000.00 | 1 | 0 | 1 | 0 | 0 | 1 | 0 | 0 | 0 | 2 | 1 | 5 | 2 | 0 | 0 | 0 | 0 |
| **K37** | Methyl | 1,000.00 | 1 | 0 | 0 | 0 | 0 | 0 | 0 | 0 | 0 | 0 | 0 | 1 | 0 | 0 | 0 | 0 | 0 |
| **K80** | Methyl | 99.36 | 1 | 8 | 8 | 10 | 9 | 10 | 7 | 10 | 13 | 6 | 14 | 14 | 12 | 8 | 12 | 14 | 14 |
| **K80** | Dimethyl | 104.64 | 1 | 6 | 7 | 6 | 7 | 6 | 7 | 5 | 8 | 9 | 6 | 10 | 10 | 5 | 6 | 8 | 7 |
| **END OF FILE** |  |  |  |  |  |  |  |  |  |  |  |  |  |  |  |  |  |  |  |

**Table 3: Acetylation of histone 3.1**

| **Current tab: PTM Spectrum Counts** | | | | | | | | | | | | | | | | | | | |
| --- | --- | --- | --- | --- | --- | --- | --- | --- | --- | --- | --- | --- | --- | --- | --- | --- | --- | --- | --- |
| **Current protein: (H31_RAT) Histone H3.1 OS=Rattus norvegicus OX=10116 PE=1 SV=3** | | | | | | | | | | | | | | | | | | | |
| **Site** | Modification | Best Ascore | Localization Probability | 133M | 148M | 23M | 37M | 123M | 124M | 131M | 132M | SD1 | SD2 | SD3 | SD4 | 147M | 149M | 150M | 151M |
| **K24** | Acetyl | 126.08 | 1 | 2 | 2 | 2 | 1 | 4 | 5 | 3 | 2 | 2 | 2 | 3 | 4 | 2 | 4 | 2 | 4 |
| **END OF FILE** |  |  |  |  |  |  |  |  |  |  |  |  |  |  |  |  |  |  |  |
